# Regioselective catalytic carbonylation and borylation of alkynes with aryldiazonium salts toward α-unsubstituted β-boryl ketones†

**DOI:** 10.1039/d2sc04867a

**Published:** 2022-09-30

**Authors:** Fengxiang Zhu, Pengpeng Yin, Xiao-Feng Wu

**Affiliations:** Department School of Chemistry and Chemical Engineering, Shanxi University Taiyuan 030006 China zfx201989@sxu.edu.cn; Dalian National Laboratory for Clean Energy, Dalian Institute of Chemical Physics, Chinese Academy of Sciences Dalian 116023 China xwu2020@dicp.ac.cn; Leibniz-Institut für Katalyse e.V. Rostock 18059 Germany xiao-feng.wu@catalysis.de

## Abstract

A new Pd/Cu-catalyzed carbonylation and borylation of alkynes with aryldiazonium salts toward α-unsubstituted β-boryl ketones with complete regioselectivity has been developed. This transformation shows broad substrate scope and excellent functional-group tolerance. Moreover, the obtained 1,2-carbonylboration products provide substantial opportunities for further transformations which cannot be obtained by known carbonylation procedures. Preliminary mechanistic studies indicate that the three hydrogen atoms of the products originated from ethyl acetate.

## Abstract 2

A multi-component procedure on the carbonylative and hydroborative synthesis of β-boryl ketones has been developed with alkynes, B_2_pin_2_ and aryldiazonium salts as the substrates and using ethyl acetate as the reagent and solvent.

## Graphical abstract

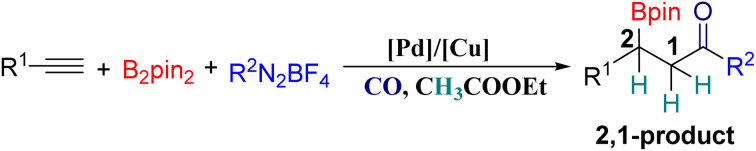

Construction of boro-containing organic molecules remains an important and hot research field due to their wide applications in materials science,^1^ pharmaceuticals^2^ and organic chemistry.^3^ A multitude of methods have been developed for the synthesis of organoboron compounds over the past decades.^4^ Among these methods, transition-metal-catalyzed borofunctionalization of alkynes is a powerful synthetic strategy due to its high selectivity and efficiency.^5^ For example, the use of copper as a precatalyst for the borylation of alkynes has generated renewed interest in the area. The β-borylalkenylcopper intermediates obtained *via syn* addition of borylcopper to alkynes can electrophilically trap various electrophiles to form different alkenylboronates (Scheme 1, 1). The classical approach of this type of transformation is alkyne hydroboration (Scheme 1, 1a).^6^ Subsequently, with vinylcopper species as the proposed key intermediates, their further reactions with halogen substitutes (Scheme 1, 1b),^7^ CO_2_ (Scheme 1, 1c),^8^ allyl phosphates (Scheme 1, 1d),^9^ and tin alkoxides (Scheme 1, 1e)^10^ to give the corresponding alkenylboronates were reported. More recently, Mankad and Cheng reported their achievements on the direct efficient synthesis of tetrasubstituted β-borylenones using a copper-catalyzed four-component coupling reaction of simple chemical feedstocks: internal alkynes, alkyl halides, bis(pinacolato)diboron (B_2_pin_2_) and CO (Scheme 1, 1f).^11^ Inspired by their achievements and considering the advantage of a multicomponent borocarbonylation reaction, we developed a new Pd/Cu-catalyzed multi-component carbonylation and borylation reaction of alkynes, aryldiazonium salts, B_2_Pin_2_, ethyl acetate and CO to obtain saturated β-boryl ketones (Scheme 1, 3). In addition, this new catalyst system can catalyze the regioselective functionalization of alkynes to obtain 2,1-carbonylboration products that are different from the 1,2-products by known transition-metal-catalyzed borylacylation (Scheme 1, 2a) and borocarbonylation (Scheme 1, 2b) of alkenes.^12^ Nevertheless, the carbonylative and hydroborative coupling of alkynes with aryldiazonium salts to obtain saturated β-boryl ketones is still a challenge and has never been reported.

**Scheme 1:**
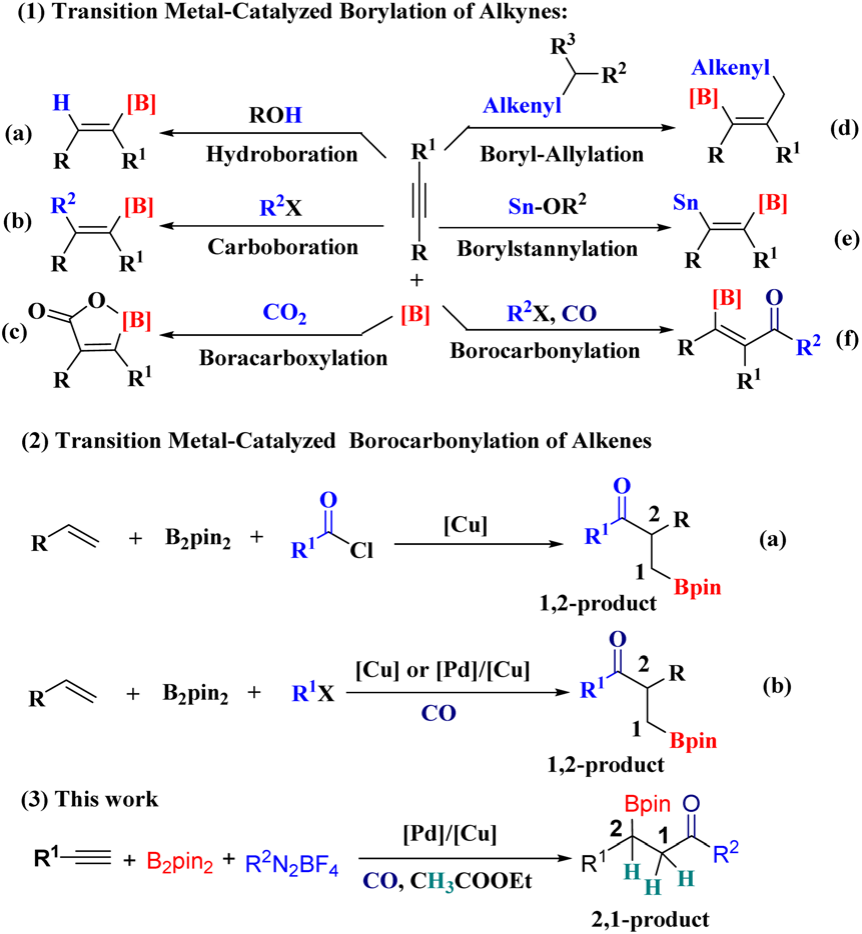
Strategies for borofunctionalization.

Initially, we tested various reaction conditions using phenyl acetylene (1a), 4-methoxybenzenediazonium tetrafluoroborate (2a) and bis(pinacolato)diboron as the reaction partners. To our delight, by using Pd(acac)_2_ and CuI as the cooperative precatalyst, PPh_3_ as the ligand, Na_2_CO_3_ as the base and ethyl acetate (EA) as the solvent at 110 °C under a CO atmosphere (20 bar) with 12 h reaction time, the desired borocarbonylative coupling product (3aa) was obtained in a good GC yield of 78% (Table 1, entry 1). When using Pd(OAc)_2_, IPrCuCl, IMesCuCl, CuCl or CuCl_2_ as the precatalyst, the reaction gave a reduced yield of the desired product (Table 1, entries 2–6). Similarly, reducing the pressure of CO (10 bar) led to a decreased yield of 3aa (Table 1, entry 7). Subsequently, ligands such as PCy_3_, DPPB, and DPEPhos and bases such as sodium *tert*-butoxide and cesium carbonate were found to be totally unsuitable for this transformation (Table 1, entries 8–12). With these results, we believe besides as a solvent, ethyl acetate (EA) also acts as a hydrogen source in this system. Then various solvents such as methanol, isopropanol and DMF which can provide a hydrogen source in many reduction reactions were tested but found to be ineffective for the reaction (Table 1, entries 13–15). Surprisingly, almost no reaction occurred using ethyl acetoacetate as the solvent which is more acidic than ethyl acetate (Table 1, entry 16). It is important to mention that by-products produced from hydroboration of alkyne can be detected during the optimization process.

**Table 1:** Optimization of the reaction conditions^a^

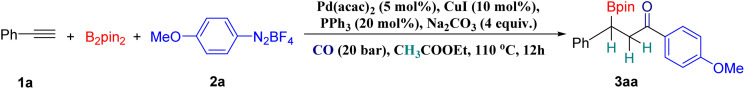		
Entry	Variation from the standard conditions	Yield (%)
1	—	78
2	Using Pd(OAc)_2_ instead of Pd(acac)_2_	44
3	Using IPrCuCl instead of CuI	41
4	Using IMesCuCl instead of CuI	38
5	Using CuCl instead of Cul	33
6	Using CuCl_2_ instead of CuI	31
7^b^	CO (10 bar) instead of CO (20 bar)	56
8	PCy_3_ instead of PPh_3_	Trace
9^c^	Using DPPB instead of PPh_3_	Trace
10^d^	Using DPEPhos instead of PPh_3_	Trace
11	Using ^*t*^BuONa instead of Na_2_CO_3_	—
12	Using Cs_2_CO_3_ instead of Na_2_CO_3_	—
13	Using MeOH instead of CH_3_COOEt	—
14	Using isopropanol instead of CH_3_COOEt	—
15	Using DMF instead of CH_3_COOEt	—
16	Using EAA instead of CH_3_COOEt	—

a Reaction conditions: 1a (0.1 mmol, 1 equiv.), B_2_pin_2_ (0.2 mmol, 2 equiv.), 2a (0.1 mmol, 1 equiv.), Pd(acac)_2_ (5 mol%), CuI (10 mol%), PPh_3_ (20 mol%), Na_2_CO_3_ (0.4 mmol, 4 equiv.), CO (20 bar), CH_3_COOEt (2 mL), stirred at 110 °C for 12 h, yields were determined by GC analysis using hexadecane as the internal standard.

b CO (10 bar).

c DPPB: 1,4-bis(diphenylphosphino)butane (10 mol%).

d DPEphos: bis[2-(diphenylphosphino)phenyl] ether (10 mol%). EAA: ethyl acetoacetate.

With the optimal reaction conditions in hand, we initially investigated the scope of alkynes for this reaction with 4-methoxybenzenediazonium tetrafluoroborate (2a) (Scheme 2). First, a variety of aryl alkynes with electron-rich and electron-deficient groups at the *para* position were successfully converted to the desired products 3aa–3ga in good to excellent yields. Similarly, *ortho*/*meta*-substituted aryl alkynes could also be converted into the corresponding products in moderate to good yields (Scheme 2, 3ha–3ka). Importantly, 3-ethynylthiophene, as an example of a heterocyclic alkyne, can be successfully reacted as well, and a good yield of the targeted product was obtained (Scheme 2, 3la). Notably, aliphatic alkynes can be effectively transformed with 4-methoxybenzenediazonium tetrafluoroborate and afforded the corresponding products in good to excellent yields (Scheme 2, 3ma–3oa). However, aromatic/aliphatic diynes, internal alkynes, 3-phenyl-1-propyne and 3-methyl-1-butyne were ineffective in our procedure.

**Scheme 2:**
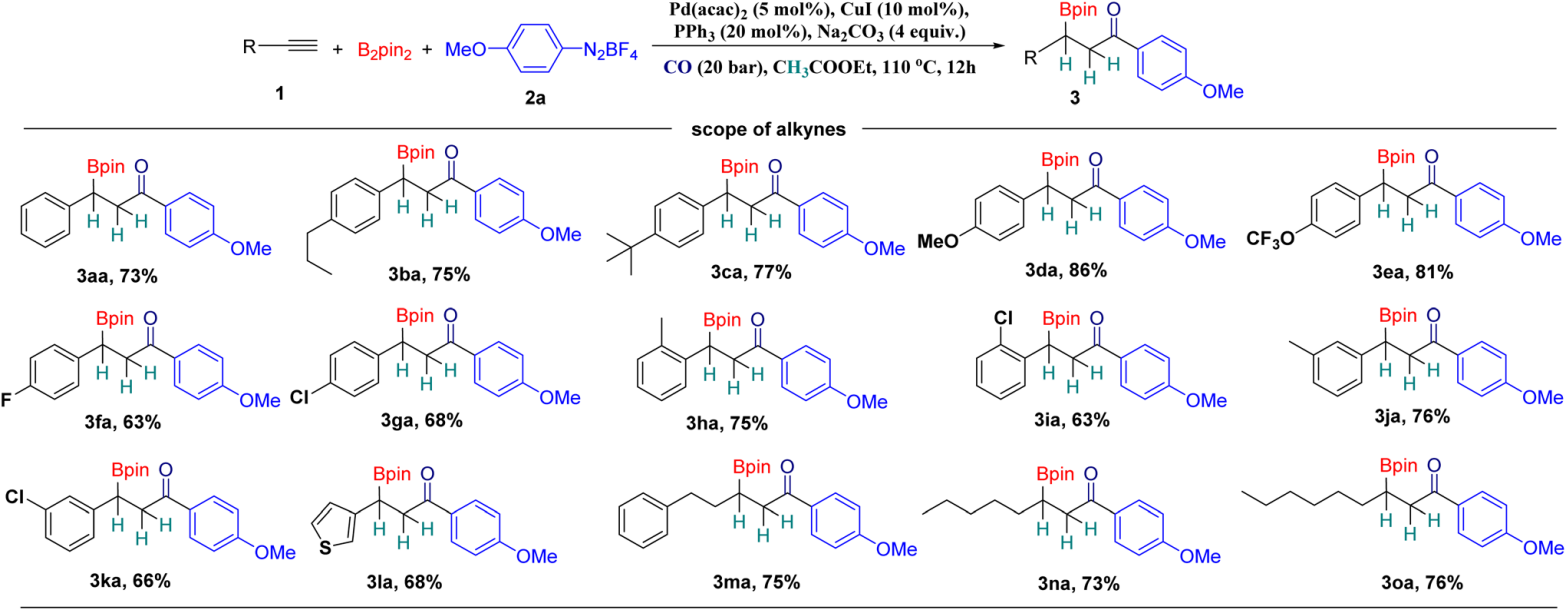
Scope of alkynes. Reaction conditions: 1 (0.1 mmol, 1 equiv.), B_2_pin_2_ (0.2 mmol, 2 equiv.), 2a (0.1 mmol, 1 equiv.), Pd(acac)_2_ (5 mol%), CuI (10 mol%), PPh_3_ (20 mol%), Na_2_CO_3_ (0.4 mmol, 4 equiv.), CO (20 bar), EA (with molecular sieves, water ≤ 50 ppm, 2 mL), stirred at 110 °C for 12 h, isolated yields.

Subsequently, with phenylacetylene (1a) as the model substrate, different aryl diazonium tetrafluoroborates were tested (Scheme 3). Aryl diazonium tetrafluoroborates with electronically neutral functional groups are all suitable substrates for this methodology and good yields can be achieved in all the tested cases (Scheme 3, 3ab–3ae). Methylthiol and phenyl groups were well tolerated under our conditions (Scheme 3, 3af–3ah). A good yield of the desired product can still be achieved with 1-naphthalenyl diazonium tetrafluoroborate (Scheme 3, 3ai). Halogen substituents can be tolerated as well, including fluoride and chloride, and good yields of the corresponding products can be obtained (Scheme 3, 3aj–3am). The bromide substituent, as an important functional group in cross-coupling transformations, can be tolerated and provide 59% of the desired product, which is ready for further functionalizations (Scheme 3, 3an).

**Scheme 3:**
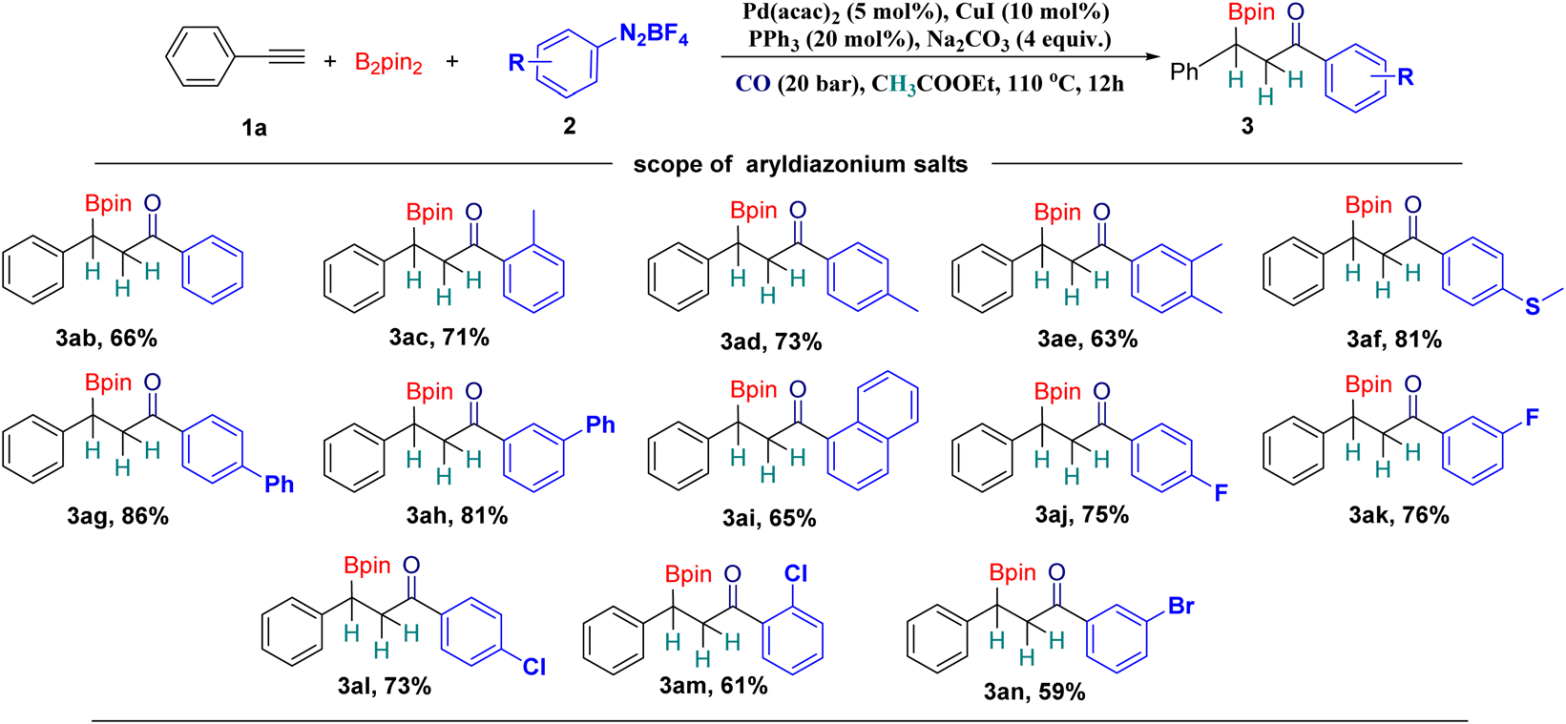
Scope of aryldiazonium salts. Reaction conditions: 1a (0.1 mmol, 1 equiv.), B_2_pin_2_ (0.2 mmol, 2 equiv.), 2 (0.1 mmol, 1 equiv.), Pd(acac)_2_ (5 mol%), CuI (10 mol%), PPh_3_ (20 mol%), Na_2_CO_3_ (0.4 mmol, 4 equiv.), CO (20 bar), EA (with molecular sieves, water ≤ 50 ppm, 2 mL), stirred at 110 °C for 12 h, isolated yields.

To understand the mechanism of this carbonylation process, a radical quenching experiment was designed to probe the mechanism of this reaction (Scheme 4). The reaction was fully inhibited when 3 equivalents of TEMPO were added to the model system (Scheme 4, a). The result shows that the radical intermediate may participate in the process. Next, we carried out the reaction in the absence of 4-methoxybenzenediazonium tetrafluoroborate (2a) and carbon monoxide, and alkenylboronic esters were obtained. Then 2a was added, and the reaction continued under the standard conditions but no corresponding product was produced (Scheme 4, b-1). Under identical reaction conditions, but in the absence of B_2_pin_2_, the carbonylative coupling product (4a) was obtained in an excellent GC yield of 95%. Surprisingly, the desired product 3aa could be obtained in 90% yield when B_2_pin_2_ was added (Scheme 4, b-2).

**Scheme 4:**
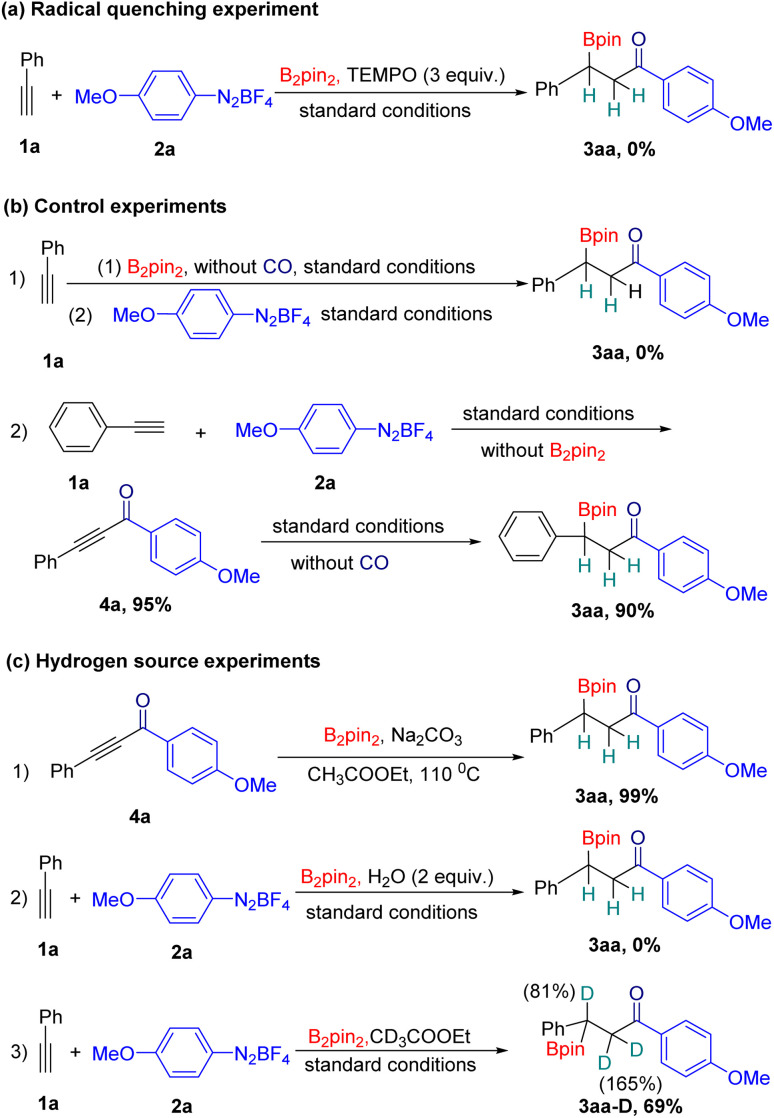
Mechanistic studies.

Finally, to gain insight into the hydrogen source of this reaction, alkynone (4a) was subjected to standard conditions without any catalyst and CO (Scheme 4, c-1). The results revealed that the hydrogen source cannot come from the terminal hydrogen of phenylacetylene. No reaction occurred when the experiment was performed in ultra-dry solvent and 2 equivalents of water under standard conditions (Scheme 4, c-2), which indicated that water should not be a hydrogen source for this reaction. Interestingly, when using CD_3_COOEt as the solvent, the deuterated product 3aa-D could be obtained in 69% yield (Scheme 4, c-3). According to the reaction results, we believe that the hydrogen came from ethyl acetate.

Based on the above control experiments and related literature,^13,14^ a possible reaction pathway is proposed (Scheme 5). Initially, Pd(0) precursor A will react with 2 to give the aryl Pd(ii) complex along with the release of N_2_. Subsequent CO insertion into the C–Pd bond affords palladium carbonyl intermediate B. Terminal alkynes 1 react with CuI to produce alkynyl Cu intermediate C, which will transmetalate with Pd(ii) species B. Then the produced palladium carbonyl intermediate D gives alkynone 4 and Pd(0) species by reductive elimination. Alkynone 4 together with B_2_pin_2_ in the presence of ethyl acetate will generate vinyl-boronate 5, and then another equivalent of B_2_pin_2_ will add to the carbon–carbon double bond allowing the formation of 1,1,2-tris(boronate) 6 which is not very stable under basic conditions.^14^ For this reason, compound 6 undergoes selective protodeboronation to generate 1,1-diboronate esters 7 which will undergo further protodeboronation to give the final product 3, and this part is most likely radical involved.

**Scheme 5:**
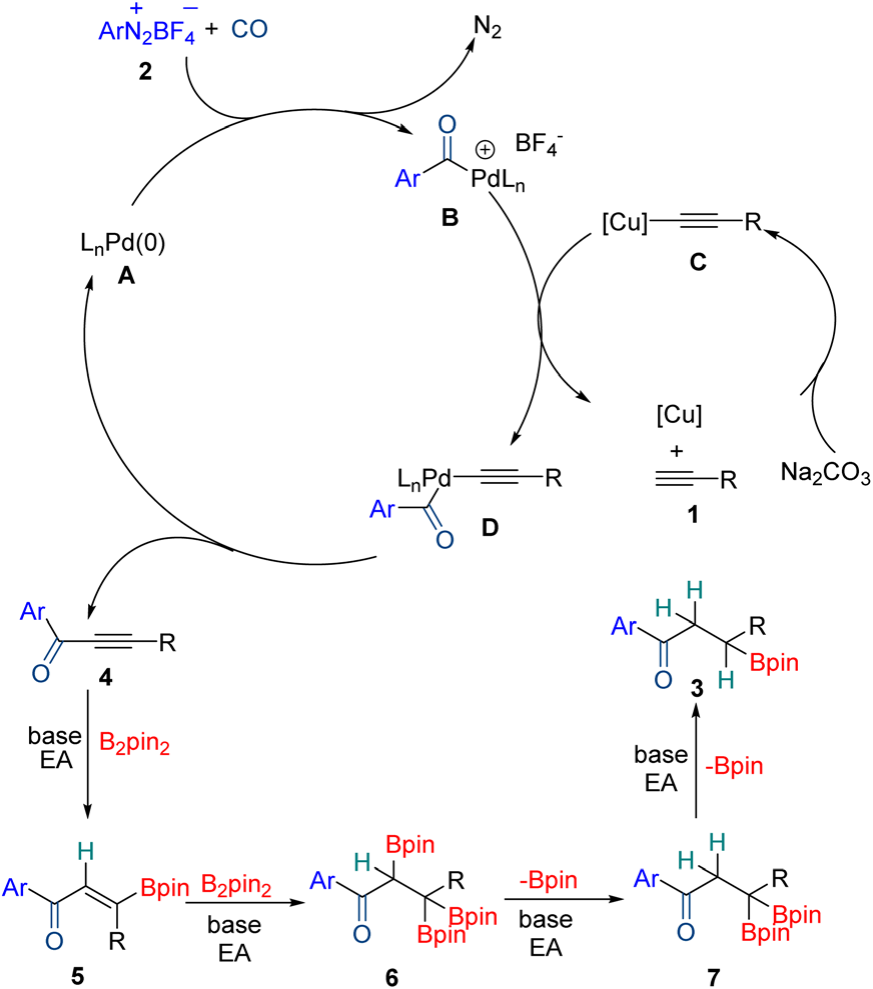
Proposed mechanism.

In summary, we have described a convenient procedure to synthesize saturated β-boryl ketones *via* cooperative Pd/Cu-catalyzed multi-component carbonylation and borylation reaction of alkynes, aryldiazonium salts, B_2_pin_2_, ethyl acetate and CO. In addition, this reaction proceeds with broad scope and functional group tolerance, and delivers β-boryl ketones in moderate to excellent yields. Mechanistic research shows that the three hydrogen atoms come from ethyl acetate.

## Author contributions

FZ and XFW directed this project, prepared and revised the manuscript. PY performed all the experiments.

## Conflicts of interest

There is no conflict of interests to declare.

## Supplementary Material

SC-013-D2SC04867A-s001
