# Supplementary material for: Regioselective catalytic carbonylation and borylation of alkynes with aryldiazonium salts toward α-unsubstituted β-boryl ketones
Source: Chem Sci. 2022 Sep 30;13(41):12122–6. doi: 10.1039/d2sc04867a (PMC9600224; doi:10.1039/d2sc04867a)

Electronic Supplementary Material (ESI) for Chemical Science.
This journal is © The Royal Society of Chemistry 2022

Electronic Supplementary Information For

Regioselective Catalytic Carbonylation and Borylation of Alkynes with Aryldiazonium Salts Toward α-Unsubstituted β-Boryl Ketones

Fengxiang Zhu,*[a] Pengpeng Yin[a] and Xiao-Feng Wu*[b]

[a] School of Chemistry and Chemical Engineering, Shanxi University, Taiyuan 030006 (China).

[b] Dalian National Laboratory for Clean Energy, Dalian Institute of Chemical Physics, Chinese Academy of Sciences, Dalian 116023 (China); Leibniz-Institut für Katalyse e.V., Rostock 18059 (Germany)

E-mail: zfx201989@sxu.edu.cn (Fengxiang Zhu); xwu2020@dicp.ac.cn; xiao-feng.wu@catalysis.de (Xiao-Feng Wu).

Table of Content

1. General Methods S2
2. Typical reaction procedure for the synthesis of β-boryl ketones S2
3. Characterization data for products S2
4. References S11
5. NMR Spectrum Copies S12

1. General Methods

NMR spectra were recorded on Bruker Avance NEO 600 M. Chemical shifts (ppm) are given relative to solvent: references for $CDCl_3$ were 7.26 ppm (^{1}H-NMR) and 77.0 ppm (^{13}C-NMR). ^{13}CNMR spectra were acquired on a broad band decoupled mode. Multiplets were assigned as s (singlet), d (doublet), t (triplet), dd (doublet of doublet), m (multiplet) and br. s (broad singlet). All measurements were carried out at room temperature unless otherwise stated. Gas chromatography analysis was performed on a Shimadzu 2014 instrument with an FID detector and HP-5 capillary column (polydimethylsiloxane with 5% phenyl groups, 30 m, 0.32 mm i.d., 0.25 μm film thickness) using N_2 as carrier gas. HRMS was obtained on a Bruker Daltonics Bio-TOF-Q mass spectrometer by the ESI method. The products were isolated from the reaction mixture by column chromatography on silica gel 60, 0.063-0.2 mm, 70-230 mesh. All reactions were carried out under air atmosphere. All the reagents were purchased from Heowns, Rhawn, infinity scientific, and Laajoo chemical company and used without further purification.

2. Typical reaction procedure for the synthesis of β-boryl ketones:

General procedure: A 4 ml screw-cap vial was charged with Pd(acac)$_2$ (5 mol%), CuI (10 mol%), PPh_3 (20 mol%), alkyne (0.1 mmol), aryldiazonium salt (0.1 mmol), B_2pin_2 (0.2 mmol), Na_2CO_3 (0.4 mmol), CH_3COOEt (2 mL) and a stirring bar. The vial was closed by a Teflon septum and a phenolic cap and connected to the atmosphere through a needle. Then the vial was fixed in an alloy plate and put into Paar 4560 series autoclave (300 mL). At room temperature, the autoclave is flushed with CO for three times and 20 bar of CO was charged. The autoclave was placed on a heating plate equipped with magnetic stirring and an aluminum block. The reaction was heated at 110 ^oC for 12 hours. Afterwards, the autoclave was cooled to room temperature and the pressure carefully released. Upon completion, the reaction mixture was concentrated under vacuum. The residue was purified by silica gel column chromatography using a petroleum ether/AcOEt (30:1) as the eluent to give the corresponding products.

3. Characterization data for products

1-(4-methoxyphenyl)-3-phenyl-3-(4,4,5,5-tetramethyl-1,3,2-dioxaborolan-2-yl)propan-1-one[1]

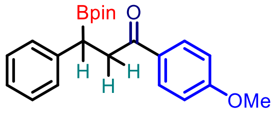

3aa

Purification by flash column chromatography (petroleum ether/EtOAc = 30:1). Colorless oil (26.7 mg, 73%). ^{1}H NMR (600 MHz, $CDCl_3$) δ 7.98 – 7.96 (m, 2H), 7.35 – 7.29 (m, 4H), 7.22 – 7.16 (m, 1H), 6.97 – 6.90 (m, 2H), 3.88 (s, 3H), 3.52 (dd, J = 18.0, 10.9 Hz, 1H), 3.41 (dd, J = 18.0, 5.1 Hz, 1H), 2.80 (dd, J = 10.9, 5.0 Hz, 1H), 1.27 (s, 6H), 1.19 (s, 6H). ^{13}C{^{1}H} NMR (151 MHz, $CDCl_3$) δ 198.25, 163.37, 142.14, 130.31, 129.89, 128.49, 128.41, 125.54, 113.62, 83.33, 55.46, 42.95, 24.58, 24.55. ^{11}B NMR (160 MHz, $CDCl_3$) δ 22.52. GC-MS (EI-70 eV): m/z (%) 366 (25), 308 (20), 283 (100), 266 (50), 239 (55), 135 (73).

1-(4-methoxyphenyl)-3-(4-propylphenyl)-3-(4,4,5,5-tetramethyl-1,3,2-dioxaborolan-2-yl) propan-1-one

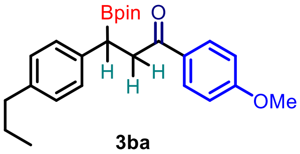

3ba

Purification by flash column chromatography (petroleum ether/EtOAc = 30:1). Colorless oil (30.6 mg, 75%). ^{1}H NMR (600 MHz, $CDCl_3$) δ 7.98 – 7.95 (m, 2H), 7.23 (d, *J* = 8.0 Hz, 2H), 7.11 (d, *J* = 8.0 Hz, 2H), 6.96 – 6.90 (m, 2H), 3.88 (s, 3H), 3.50 (dd, *J* = 18.0, 11.0 Hz, 1H), 3.39 (dd, *J* = 18.0, 5.0 Hz, 1H), 2.76 (dd, *J* = 11.0, 5.0 Hz, 1H), 2.60 – 2.54 (m, 2H), 1.65 (dd, *J* = 15.1, 7.5 Hz, 2H), 1.27 (s, 6H), 1.19 (s, 6H), 0.96 (t, *J* = 7.3 Hz, 3H). ^{13}C{^{1}H} NMR (151 MHz, $CDCl_3$) δ 198.37, 163.32, 139.78, 139.17, 130.30, 129.96, 128.58, 128.23, 113.59, 83.27, 55.45, 43.15, 37.66, 24.58, 24.56, 13.92. ^{11}B NMR (160 MHz, $CDCl_3$) δ 22.43. GC-MS (EI-70 eV): m/z (%) 408 (29) 350 (20), 325 (100), 281 (32), 264 (48). HRMS (ESI): calcd for $C_{25}H_{33}BO_4$ $[M+Na]^+$: 431.2364, found: 431.2367.

3-(4-(*tert*-butyl)phenyl)-1-(4-methoxyphenyl)-3-(4,4,5,5-tetramethyl-1,3,2-dioxaborolan-2yl)propan-1-one

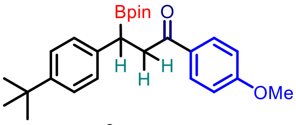

3ca

Purification by flash column chromatography (petroleum ether/EtOAc = 30:1). Colorless oil (32.4 mg, 77%). ^{1}H NMR (600 MHz, $CDCl_3$) δ 7.96 (dd, *J* = 9.3, 2.3 Hz, 2H), 7.31 (d, *J* = 8.3 Hz, 2H), 7.25 (d, *J* = 8.3 Hz, 2H), 6.94 – 6.91 (m, 2H), 3.88 (s, 3H), 3.50 (dd, *J* = 18.0, 11.0 Hz, 1H), 3.39 (dd, *J* = 18.0, 5.0 Hz, 1H), 2.77 (dd, *J* = 11.0, 4.9 Hz, 1H), 1.33 (s, 9H), 1.28 (s, 6H), 1.20 (s, 6H). ^{13}C{^{1}H} NMR (151 MHz, $CDCl_3$) δ 198.36, 163.31, 148.18, 138.89, 130.30, 129.98, 128.03, 125.39, 113.58, 83.28, 55.46, 43.23, 34.32, 31.43, 24.63, 24.60. ^{11}B NMR (160 MHz, $CDCl_3$) δ 22.47. GC-MS (EI-70 eV): m/z (%) 422(30), 365 (27), 339 (100), 322 (20), 278 (35), 221 (48). HRMS (ESI): calcd for $C_{26}H_{35}BO_4$ $[M+Na]^+$: 445.2521, found: 445.2513.

1-(4-methoxyphenyl)-3-(4,4,5,5-tetramethyl-1,3,2-dioxaborolan-2-yl)-3-(4 (trifluoromethoxy)phenyl)propan-1-one

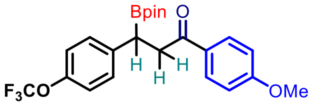

3da

Purification by flash column chromatography (petroleum ether/EtOAc = 30:1). Colorless oil (36.4 mg, 81%). ^{1}H NMR (600 MHz, $CDCl_3$) δ 7.98 – 7.95 (m, 2H), 7.35 – 7.32 (m, 2H), 7.14 (d, *J* = 8.0 Hz, 2H), 6.95 – 6.92 (m, 2H), 3.88 (s, 3H), 3.44 (ddd, *J* = 23.4, 18.0, 7.9 Hz, 2H), 2.81 (dd, *J* = 10.3, 5.4 Hz, 1H), 1.27 (s, 6H), 1.20 (s, 6H). ^{13}C{^{1}H} NMR (151 MHz, $CDCl_3$) δ 197.88, 163.49, 147.22, 141.01, 130.34, 129.70, 129.61, 121.00, 120.54 (q, *J* = 256.70Hz), 113.67, 83.50, 55.48, 42.77, 24.57, 24.56. ^{19}F NMR (471 MHz, $CDCl_3$) δ -57.88. ^{11}B NMR (160 MHz, $CDCl_3$) δ 22.52. GC-MS (EI-70 eV): m/z (%) 450 (28), 392 (20), 367 (100), 349 (25), 306 (48), 265 (22), 190 (23). HRMS (ESI): calcd for $C_{23}H_{26}BF_3O_5$ $[M+Na]^+$: 473.1718, found: 473.1718.

3-(4-fluorophenyl)-1-(4-methoxyphenyl)-3-(4,4,5,5-tetramethyl-1,3,2-dioxaborolan-2-yl)propan-1-one

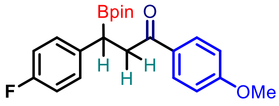

3ea

Purification by flash column chromatography (petroleum ether/EtOAc = 30:1). White solid (24.1 mg, 63%). ^{1}H NMR (600 MHz, $CDCl_3$) δ 7.98 – 7.95 (m, 2H), 7.27 (dd, *J* = 6.0, 2.6 Hz, 2H), 7.00 – 6.96 (m, 2H), 6.95 – 6.92 (m, 2H), 3.88 (s, 3H), 3.47 (dd, *J* = 17.9, 10.5 Hz, 1H), 3.39 (dd, *J* = 18.0, 5.4 Hz, 1H), 2.78 (dd, *J* = 10.4, 5.4 Hz, 1H), 1.26 (s, 6H), 1.19 (s, 6H). ^{13}C{^{1}H} NMR (151 MHz, $CDCl_3$) δ 198.05, 163.43, 161.14 (d, *J* = 243.11 Hz), 137.72 (d, *J* = 3.2 Hz), 130.32, 129.77 (d, *J* = 7.55 Hz), 129.69, 115.20 (d, *J* = 21.14 Hz), 113.64, 83.41, 55.47, 42.95, 24.58, 24.54. ^{19}F NMR (471 MHz, $CDCl_3$) δ -118.26. ^{11}B NMR (160 MHz, $CDCl_3$) δ 22.51. GC-MS (EI-70 eV): m/z (%) 384 (28), 326 (20), 301 (100), 257 (53), 135 (52). HRMS (ESI): calcd for $C_{22}H_{26}BFO_4$ $[M+Na]^+$: 407.1800, found: 407.1790.

3-(4-chlorophenyl)-1-(4-methoxyphenyl)-3-(4,4,5,5-tetramethyl-1,3,2-dioxaborolan-2-yl)propan-1-one

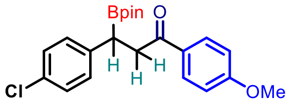

3fa

Purification by flash column chromatography (petroleum ether/EtOAc = 30:1). White solid (27.2 mg, 68%). ^{1}H NMR (600 MHz, $CDCl_3$) δ 7.96 (d, *J* = 8.9 Hz, 2H), 7.26 (s, 4H), 6.94 (d, *J* = 8.9 Hz, 2H), 3.88 (s, 3H), 3.47 (dd, *J* = 18.0, 10.4 Hz, 1H), 3.39 (dd, *J* = 17.9, 5.4 Hz, 1H), 2.77 (dd, *J* = 10.4, 5.4 Hz, 1H), 1.26 (s, 6H), 1.19 (s, 6H). ^{13}C{^{1}H} NMR (151 MHz, $CDCl_3$) δ 197.92, 163.47, 140.72, 131.24, 130.33, 129.74, 129.72, 128.55, 113.67, 83.46, 55.48, 42.65, 24.58, 24.55. ^{11}B NMR (160 MHz, $CDCl_3$) δ 22.55. GC-MS (EI-70 eV): m/z (%) 400 (28), 342 (20), 317 (100), 300 (45), 273 (49), 221 (30), 135 (55). HRMS (ESI): calcd for $C_{22}H_{26}BClO_4$ $[M+Na]^+$: 423.1505, found: 423.1503.

1-(4-methoxyphenyl)-3-(4,4,5,5-tetramethyl-1,3,2-dioxaborolan-2-yl)-3-(o-tolyl)propan-1-one

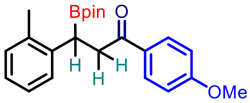

3ga

Purification by flash column chromatography (petroleum ether/EtOAc = 30:1). Colorless oil (28.5 mg, 75%). ^{1}H NMR (600 MHz, $CDCl_3$) δ 7.97 (d, *J* = 8.9 Hz, 2H), 7.33 (d, *J* = 7.5 Hz, 1H), 7.16 (dd, *J* = 11.8, 7.4 Hz, 2H), 7.09 (t, *J* = 7.1 Hz, 1H), 6.93 (d, *J* = 8.9 Hz, 2H), 3.88 (s, 3H), 3.49 (dd, *J* = 18.0, 10.8 Hz, 1H), 3.33 (dd, *J* = 18.0, 4.8 Hz, 1H), 3.03 (dd, *J* = 10.8, 4.8 Hz, 1H), 2.40 (s, 3H), 1.27 (s, 6H), 1.19 (s, 6H). ^{13}C{^{1}H} NMR (151 MHz, $CDCl_3$) δ 198.41, 163.34, 140.53, 136.43, 130.46, 130.30, 129.96, 127.77, 126.01, 125.44, 113.61, 83.27, 55.47, 42.30, 24.63, 24.56, 20.09. ^{11}B NMR (160 MHz, $CDCl_3$) δ 22.64. GC-MS (EI-70 eV): m/z (%) 380 (15), 297 (24), 236 (100), 221 (20), 121(23). HRMS (ESI): calcd for $C_{23}H_{29}BO_4$ $[M+Na]^+$: 403.2051, found: 403.2057.

3-(2-chlorophenyl)-1-(4-methoxyphenyl)-3-(4,4,5,5-tetramethyl-1,3,2-dioxaborolan-2-yl) propan-1-one

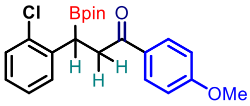

3ha

Purification by flash column chromatography (petroleum ether/EtOAc = 30:1). Colorless oil (25.2 mg, 63%). ^{1}H NMR (600 MHz, $CDCl_3$) δ 7.99 – 7.92 (m, 2H), 7.44 (dd, *J* = 7.7, 1.6 Hz, 1H), 7.36 (dd, *J* = 7.9, 1.3 Hz, 1H), 7.19 (td, *J* = 7.5, 1.3 Hz, 1H), 7.13 – 7.10 (m, 1H), 6.95 – 6.90 (m, 2H), 3.88 (s, 3H), 3.50 – 3.38 (m, 2H), 3.29 (t, *J* = 7.2 Hz, 1H), 1.30 (s, 6H), 1.25 (s, 6H). ^{13}C{^{1}H} NMR (151 MHz, $CDCl_3$) δ 197.86, 163.37, 140.29, 134.32, 130.57, 130.34, 129.88, 129.61, 126.98, 126.82, 113.61, 83.56, 55.46, 41.30, 24.76, 24.67. ^{11}B NMR (160 MHz, $CDCl_3$) δ 22.50. GC-MS (EI-70 eV): m/z (%) 400 (35), 365 (20), 317 (100), 300 (48), 273 (72), 221 (30), 135 (90). HRMS (ESI): calcd for $C_{22}H_{26}BClO_4$ $[M+Na]^+$: 423.1505, found: 423.1510.

1-(4-methoxyphenyl)-3-(4,4,5,5-tetramethyl-1,3,2-dioxaborolan-2-yl)-3-(m-tolyl)propan-1-one

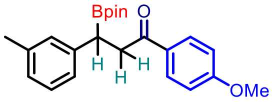

3ia

Purification by flash column chromatography (petroleum ether/EtOAc = 30:1). Colorless oil (28.8 mg, 76%). ^{1}H NMR (600 MHz, $CDCl_3$) δ 7.97 (dd, *J* = 9.2, 2.2 Hz, 2H), 7.20 (t, *J* = 7.5 Hz, 1H), 7.15 – 7.12 (m, 2H), 7.00 (d, *J* = 7.4 Hz, 1H), 6.95 – 6.91 (m, 2H), 3.88 (s, 3H), 3.51 (dd, *J* = 18.0, 11.1 Hz, 1H), 3.39 (dd, *J* = 18.0, 5.0 Hz, 1H), 2.76 (dd, *J* = 11.1, 4.9 Hz, 1H), 2.35 (s, 3H), 1.27 (s, 6H), 1.19 (s, 6H). ^{13}C{^{1}H} NMR (151 MHz, $CDCl_3$) δ 198.32, 163.35, 142.01, 138.01, 130.31, 129.92, 129.28, 128.37, 126.32, 125.35, 113.61, 83.30, 55.46, 43.06, 24.57, 24.55, 21.46. ^{11}B NMR (160 MHz, $CDCl_3$) δ 22.56. GC-MS (EI-70 eV): m/z (%) 380 (40), 322 (20), 297 (100), 236 (70), 135 (38). HRMS (ESI): calcd for $C_{23}H_{29}BO_4$ $[M+Na]^+$: 403.2051, found: 403.2049.

3-(3-chlorophenyl)-1-(4-methoxyphenyl)-3-(4,4,5,5-tetramethyl-1,3,2-dioxaborolan-2-yl) propan-1-one

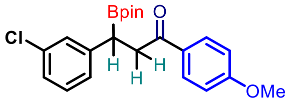

3ja

Purification by flash column chromatography (petroleum ether/EtOAc = 30:1). Colorless oil (26.4 mg, 66%). ^{1}H NMR (600 MHz, $CDCl_3$) δ 7.99 – 7.94 (m, 2H), 7.32 (s, 1H), 7.28 – 7.13 (m, 3H), 6.97 – 6.92 (m, 2H), 3.89 (s, 3H), 3.49 (dd, *J* = 18.0, 10.6 Hz, 1H), 3.40 (dd, *J* = 18.0, 5.2 Hz, 1H), 2.78 (dd, *J* = 10.6, 5.2 Hz, 1H), 1.27 (s, 6H), 1.20 (s, 6H). ^{13}C{^{1}H} NMR (151 MHz, $CDCl_3$) δ 197.85, 163.49, 144.33, 134.17, 130.35, 129.68, 129.66,128.43, 126.68, 125.75, 113.67, 83.51, 55.48, 42.55, 24.55. ^{11}B NMR (160 MHz, $CDCl_3$) δ 22.53. GC-MS (EI-70 eV): m/z (%) 400 (30), 342 (23), 317 (100), 300 (63), 273 (56), 135 (90). HRMS (ESI): calcd for $C_{22}H_{26}BClO_4$ $[M+Na]^+$: 423.1505, found: 423.1501.

1-(4-methoxyphenyl)-3-(4,4,5,5-tetramethyl-1,3,2-dioxaborolan-2-yl)-3-(thiophen-3-yl) propan-1-one

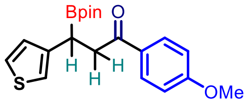

3ka

Purification by flash column chromatography (petroleum ether/EtOAc = 30:1). Yellow oil (25.2 mg, 68%). ^{1}HNMR (600 MHz, $CDCl_3$) δ 7.99 – 7.96 (m, 2H), 7.26 (dd, *J* = 4.9, 2.9 Hz, 1H), 7.06 (dd, *J* = 21.2, 3.7 Hz, 2H), 6.95 – 6.92 (m, 2H), 3.89 (s, 3H), 3.51 – 3.41 (m, 2H), 2.90 (dd, *J* = 10.1, 5.5 Hz, 1H), 1.27 (s, 6H), 1.21 (s, 6H). $^{13}C\{^1H\}$ NMR (151 MHz, $CDCl_3$) δ 198.13, 163.41, 141.82, 130.33, 129.84, 128.34, 125.13, 119.69, 113.64, 83.38, 55.47, 42.24, 24.63, 24.55. ^{11}B NMR (160 MHz, $CDCl_3$) δ 22.46. GC-MS (EI-70 eV): m/z (%) 372 (65), 314 (40), 289 (100), 272 (35), 245 (46), 228 (48). HRMS (ESI): calcd for $C_{20}H_{25}BO_4S$ $[M+Na]^+$: 395.1459, found: 395.1459.

1-(4-methoxyphenyl)-5-phenyl-3-(4,4,5,5-tetramethyl-1,3,2-dioxaborolan-2-yl)pentan-1-one

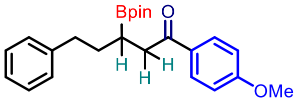

3la

Purification by flash column chromatography (petroleum ether/EtOAc = 30:1). Colorless oil (29.5 mg, 75%). ^{1}H NMR (600 MHz, $CDCl_3$) δ 8.01 (d, *J* = 8.8 Hz, 2H), 7.19 (t, *J* = 7.5 Hz, 2H), 7.10 (dd, *J* = 11.7, 7.3 Hz, 3H), 6.94 (d, *J* = 8.8 Hz, 2H), 3.89 (s, 3H), 3.42 (s, 2H), 2.54 – 2.47 (m, 2H), 2.08 – 1.96 (m, 2H), 1.68 (s, 1H), 1.31 (s, 6H), 1.28 (s, 6H). $^{13}C\{^1H\}$ NMR (151 MHz, $CDCl_3$) δ 198.56, 163.22, 143.27,130.43, 130.26, 128.46, 128.04, 125.35, 113.49, 83.11, 55.45, 39.96, 34.44, 32.68, 24.91, 24.76. ^{11}B NMR (160 MHz, $CDCl_3$) δ 22.22. GC-MS (EI-70 eV): m/z (%) 394 (13), 311 (21), 289 (20), 232 (23), 189 (30), 150 (100). HRMS (ESI): calcd for $C_{24}H_{31}BO_4$ $[M+Na]^+$: 417.2208, found: 417.2211.

1-(4-methoxyphenyl)-3-(4,4,5,5-tetramethyl-1,3,2-dioxaborolan-2-yl)octan-1-one

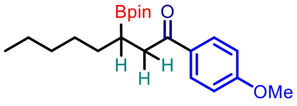

3ma

Purification by flash column chromatography (petroleum ether/EtOAc = 50:1). Colorless oil (26.2 mg, 73%). ^{1}H NMR (600 MHz, $CDCl_3$) δ 7.96 (d, *J* = 8.4 Hz, 2H), 6.93 (d, *J* = 8.5 Hz, 2H), 3.88 (s, 3H), 3.09 (qd, *J* = 17.8, 6.7 Hz, 2H), 1.42 – 1.35 (m, 4H), 1.34 – 1.26 (m, 11H), 1.25 (s, 6H), 0.89 (t, *J* = 6.8 Hz, 3H). $^{13}C\{^1H\}$ NMR (151 MHz, $CDCl_3$) δ 199.03, 163.20, 130.26, 113.54, 82.87, 55.44, 40.72, 32.08, 30.62, 28.74, 24.81, 24.71, 22.60, 14.08. ^{11}B NMR (160 MHz, $CDCl_3$) δ 22.58. GC-MS (EI-70 eV): m/z (%) 360 (1), 345 (10), 231 (18), 189 (10), 150 (100), 133 (28). HRMS (ESI): calcd for $C_{21}H_{33}BO_4$ $[M+Na]^+$: 383.2364, found: 383.2359.

1-(4-methoxyphenyl)-3-(4,4,5,5-tetramethyl-1,3,2-dioxaborolan-2-yl)nonan-1-one

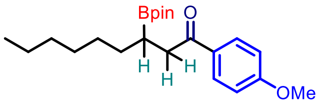

3na

Purification by flash column chromatography (petroleum ether/EtOAc = 50:1). Colorless oil (28.5 mg, 76%). ^{1}H NMR (600 MHz, $CDCl_3$) δ 8.04 – 7.97 (m, 2H), 6.95 – 6.89 (m, 2H), 3.88 (s, 3H), 3.34 (s, 2H), 1.27 (d, *J* = 12.0 Hz, 23H), 0.82 (t, *J* = 6.9 Hz, 3H). ^{13}C{^{1}H} NMR (151 MHz, $CDCl_3$) δ 198.73, 163.14, 130.40, 130.35, 113.44, 82.98, 55.42, 39.71, 31.86, 30.09, 29.89, 27.76, 24.83, 24.75, 22.60, 14.06. ^{11}B NMR (160 MHz, $CDCl_3$) δ 22.52. GC-MS (EI-70 eV): m/z (%) 374 (1), 289 (5), 231 (18), 189 (15), 150 (100). HRMS (ESI): calcd for $C_{22}H_{35}BO_4$ $[M+Na]^+$: 397.2521, found: 397.2517.

1,3-diphenyl-3-(4,4,5,5-tetramethyl-1,3,2-dioxaborolan-2-yl)propan-1-one[2]

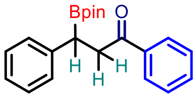

3ab

Purification by flash column chromatography (petroleum ether/EtOAc = 50:1). Colorless oil (22.0 mg, 66%). ^{1}H NMR (600 MHz, $CDCl_3$) δ 7.99 (dd, *J* = 8.3, 1.2 Hz, 2H), 7.60 – 7.54 (m, 1H), 7.48 – 7.45 (m, 2H), 7.34 – 7.29 (m, 4H), 7.24 – 7.15 (m, 1H), 3.58 (dd, *J* = 18.2, 10.9 Hz, 1H), 3.49 – 3.39 (m, 1H), 2.83 (dd, *J* = 10.9, 4.9 Hz, 1H), 1.27 (s, 6H), 1.19 (s, 6H). ^{13}C{^{1}H} NMR (151 MHz, $CDCl_3$) δ 199.72, 141.96, 136.80, 132.94, 128.53, 128.50, 128.40, 128.07, 125.61, 83.41, 43.28, 24.58, 24.54. ^{11}B NMR (160 MHz, $CDCl_3$) δ 22.53. GC-MS (EI-70 eV): m/z (%) 336 (2), 278 (20), 236 (75), 209 (62), 115 (42),103 (100).

3-phenyl-3-(4,4,5,5-tetramethyl-1,3,2-dioxaborolan-2-yl)-1-(o-tolyl)propan-1-one

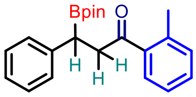

3ac

Purification by flash column chromatography (petroleum ether/EtOAc = 50:1). Colorless oil (24.8 mg, 71%). ^{1}H NMR (600 MHz, $CDCl_3$) δ 7.67 (dd, *J* = 8.0, 1.0 Hz, 1H), 7.37 (td, *J* = 7.5, 1.2 Hz, 1H), 7.31 – 7.28 (m, 4H), 7.26 – 7.23 (m, 2H), 7.18 (ddd, *J* = 8.6, 6.2, 2.3 Hz, 1H), 3.50 (dd, *J* = 18.3, 10.9 Hz, 1H), 3.36 (dd, *J* = 18.3, 5.1 Hz, 1H), 2.82 (dd, *J* = 10.9, 5.1 Hz, 1H), 2.51 (s, 3H), 1.28 (s, 6H), 1.21 (s, 6H). ^{13}C{^{1}H} NMR (151 MHz, $CDCl_3$) δ 203.86, 141.89, 138.01, 137.71, 131.81, 131.13, 128.52, 128.50,128.38, 125.58, 125.56, 83.41, 46.06, 24.61, 21.22. ^{11}B NMR (160 MHz, $CDCl_3$) δ 22.54. GC-MS (EI-70 eV): m/z (%) 350(6), 335 (100), 292 (23), 267 (32), 206 (50), 117 (56). HRMS (ESI): calcd for $C_{22}H_{27}BO_3$ $[M+Na]^+$: 373.1945, found: 373.1949.

3-phenyl-3-(4,4,5,5-tetramethyl-1,3,2-dioxaborolan-2-yl)-1-(p-tolyl)propan-1-one[3]

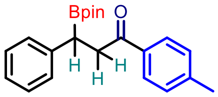

3ad

Purification by flash column chromatography (petroleum ether/EtOAc = 50:1). Colorless oil (25.5 mg, 73%). ^{1}H NMR (600 MHz, $CDCl_3$) δ 7.92 – 7.87 (m, 2H), 7.34 – 7.29 (m, 4H), 7.26 (d, *J* = 8.0 Hz, 2H), 7.21 – 7.16 (m,

1H), 3.55 (dd, J = 18.2, 11.0 Hz, 1H), 3.43 (dd, J = 18.2, 5.0 Hz, 1H), 2.81 (dd, J = 10.9, 5.0 Hz, 1H), 2.42 (s, 3H), 1.27 (s, 6H), 1.19 (s, 6H). $^{13}C\{^{1}H\}$ NMR (151 MHz, $CDCl_3$) δ 199.34, 143.66, 142.07, 134.32, 129.17, 128.50, 128.41, 128.19, 125.56, 83.36, 43.17, 24.58, 24.55, 21.65. ^{11}B NMR (160 MHz, $CDCl_3$) δ 22.50. GC-MS (EI-70 eV): m/z (%) 350 (5), 335 (51), 292 (24), 267 (82), 223 (78), 117 (100).

1-(3,4-dimethylphenyl)-3-phenyl-3-(4,4,5,5-tetramethyl-1,3,2-dioxaborolan-2-yl) propan-1-one

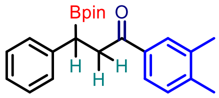

3ae

Purification by flash column chromatography (petroleum ether/EtOAc = 50:1). Colorless oil (22.9 mg, 63%). ^{1}H NMR (600 MHz, $CDCl_3$) δ 7.77 – 7.71 (m, 2H), 7.32 (ddd, J = 22.2, 10.8, 4.8 Hz, 4H), 7.19 (ddd, J = 8.6, 6.5, 4.9 Hz, 2H), 3.54 (dd, J = 18.2, 11.0 Hz, 1H), 3.42 (dd, J = 18.2, 5.0 Hz, 1H), 2.80 (dd, J = 11.0, 4.9 Hz, 1H), 2.32 (d, J = 4.9 Hz, 6H), 1.27 (s, 6H), 1.19 (s, 6H). $^{13}C\{^{1}H\}$ NMR (151 MHz, $CDCl_3$) δ 199.57, 142.38, 142.15, 136.78, 134.72, 129.71, 129.21, 128.49, 128.42, 125.80, 125.54, 83.33, 43.24, 24.58, 24.56, 20.02, 19.75. ^{11}B NMR (160 MHz, $CDCl_3$) δ 22.50. GC-MS (EI-70 eV): m/z (%) 364 (3), 349 (100), 306 (20), 281 (50), 237 (48), 131 (50). HRMS (ESI): calcd for $C_{23}H_{29}BO_3$ $[M+Na]^+$: 387.2102, found: 387.2107.

1-(4-(methylthio)phenyl)-3-phenyl-3-(4,4,5,5-tetramethyl-1,3,2-dioxaborolan-2-yl)propan-1-one

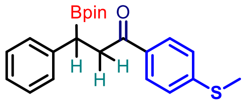

3af

Purification by flash column chromatography (petroleum ether/EtOAc = 50:1). Yellow oil (30.9 mg, 81%). ^{1}H NMR (600 MHz, $CDCl_3$) δ 7.91 – 7.88 (m, 2H), 7.34 – 7.28 (m, 4H), 7.28 – 7.25 (m, 2H), 7.21 – 7.17 (m, 1H), 3.53 (dd, J = 18.1, 10.9 Hz, 1H), 3.40 (dd, J = 18.1, 5.0 Hz, 1H), 2.81 (dd, J = 10.9, 5.0 Hz, 1H), 2.53 (s, 3H), 1.27 (s, 6H), 1.19 (s, 6H). $^{13}C\{^{1}H\}$ NMR (151 MHz, $CDCl_3$) δ 198.69, 145.58, 142.00, 133.13, 128.52, 128.49,128.40, 125.60, 124.96, 83.38, 43.06, 24.58, 24.55, 14.83. ^{11}B NMR (160 MHz, $CDCl_3$) δ 22.50. GC-MS (EI-70 eV): m/z (%) 382 (38), 335 (52), 324 (25), 299 (81), 238 (60), 191 (62),151 (100). HRMS (ESI): calcd for $C_{22}H_{27}BO_3S$ $[M+Na]^+$: 405.1666, found: 405.1661.

1-([1,1'-biphenyl]-4-yl)-3-phenyl-3-(4,4,5,5-tetramethyl-1,3,2-dioxaborolan-2-yl)propan-1-one

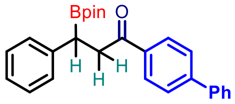

3ag

Purification by flash column chromatography (petroleum ether/EtOAc = 50:1). Colorless oil (35.4 mg, 86%). ^{1}H NMR (600 MHz, $CDCl_3$) δ 8.07 (d, J = 8.3 Hz, 2H), 7.70 – 7.64 (m, 4H), 7.49 (t, J = 7.7 Hz, 2H), 7.42 (t, J = 7.4 Hz, 1H), 7.33 (dt, J = 15.2, 7.7 Hz, 4H), 7.20 (t, J = 7.2 Hz, 1H), 3.61 (dd, J = 18.2, 10.9 Hz, 1H), 3.48 (dd, J = 18.2, 5.0 Hz, 1H), 2.85 (dd, J = 10.9, 4.9 Hz, 1H), 1.28 (s, 6H), 1.21 (s, 6H). $^{13}C\{^{1}H\}$ NMR (151 MHz, $CDCl_3$) δ 199.32, 145.63, 141.99, 139.99, 135.50, 128.95, 128.66, 128.55, 128.42, 128.18, 127.29, 127.17, 125.62, 83.42,

43.34, 24.60, 24.56. ^{11}B NMR (160 MHz, $CDCl_3$) δ 22.53. GC-MS (EI-70 eV): m/z (%) 412 (28), 354 (20), 329 (100), 312 (52), 285 (60), 268 (58), 152 (32). HRMS (ESI): calcd for $C_{27}H_{29}BO_3$ $[M+Na]^+$: 435.2102, found: 435.2096.

1-([1,1'-biphenyl]-3-yl)-3-phenyl-3-(4,4,5,5-tetramethyl-1,3,2-dioxaborolan-2-yl)propan-1-one

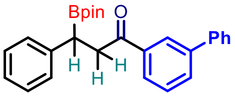

3ah

Purification by flash column chromatography (petroleum ether/EtOAc = 50:1). Colorless oil (33.3 mg, 81%). ^{1}H NMR (600 MHz, $CDCl_3$) δ 8.21 (t, J = 1.7 Hz, 1H), 7.99 – 7.95 (m, 1H), 7.80 (ddd, J = 7.7, 1.7, 1.1 Hz, 1H), 7.63 (dt, J = 8.1, 1.6 Hz, 2H), 7.54 (t, J = 7.7 Hz, 1H), 7.50 – 7.47 (m, 2H), 7.42 – 7.39 (m, 1H), 7.36 – 7.30 (m, 4H), 7.22 – 7.17 (m, 1H), 3.63 (dd, J = 18.2, 10.9 Hz, 1H), 3.50 (dd, J = 18.2, 4.9 Hz, 1H), 2.85 (dd, J = 10.9, 4.9 Hz, 1H), 1.28 (s, 6H), 1.20 (s, 6H). ^{13}C{^{1}H} NMR (151 MHz, $CDCl_3$) δ 199.64, 141.96, 141.65, 140.27, 137.30, 131.59, 128.99, 128.91,128.56, 128.44, 127.77, 127.23, 126.91, 126.80, 125.65, 83.44, 43.50, 24.60, 24.57. ^{11}B NMR (160 MHz, $CDCl_3$) δ 22.57. GC-MS (EI-70 eV): m/z (%) 412 (48), 329 (90), 312 (100), 285 (75), 268 (77), 179 (68), 152 (70). HRMS (ESI): calcd for $C_{27}H_{29}BO_3$ $[M+Na]^+$: 435.2102, found: 435.2100.

1-(naphthalen-1-yl)-3-phenyl-3-(4,4,5,5-tetramethyl-1,3,2-dioxaborolan-2-yl)propan-1-one

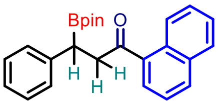

3ai

Purification by flash column chromatography (petroleum ether/EtOAc = 50:1). Colorless oil (25.0 mg, 65%). ^{1}H NMR (600 MHz, $CDCl_3$) δ 8.64 (d, J = 8.5 Hz, 1H), 7.99 (d, J = 8.2 Hz, 1H), 7.90 (t, J = 6.9 Hz, 2H), 7.61 – 7.53 (m, 2H), 7.50 (dd, J = 8.0, 7.3 Hz, 1H), 7.33 (ddd, J = 26.2, 10.8, 4.6 Hz, 4H), 7.23 – 7.18 (m, 1H), 3.70 (dd, J = 18.2, 10.9 Hz, 1H), 3.50 (dd, J = 18.2, 5.0 Hz, 1H), 2.95 (dd, J = 10.9, 5.0 Hz, 1H), 1.31 (s, 6H), 1.25 (s, 6H). ^{13}C{^{1}H} NMR (151 MHz, $CDCl_3$) δ 204.24, 141.83, 135.92, 133.92, 132.36, 130.12, 128.56, 128.43, 128.34, 127.70, 127.37, 126.37, 125.93, 125.64, 124.42, 83.53, 46.63, 24.67. ^{11}B NMR (160 MHz, $CDCl_3$) δ 22.52. GC-MS (EI-70 eV): m/z (%) 386 (76), 342 (100), 328 (23), 285(26), 259 (25), 153 (49), 127 (70). HRMS (ESI): calcd for $C_{25}H_{27}BO_3$ $[M+Na]^+$: 409.1945, found: 409.1940.

1-(4-fluorophenyl)-3-phenyl-3-(4,4,5,5-tetramethyl-1,3,2-dioxaborolan-2-yl)propan-1-one[3]

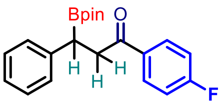

3aj

Purification by flash column chromatography (petroleum ether/EtOAc = 50:1). Colorless oil (26.5 mg, 75%). ^{1}H NMR (600 MHz, $CDCl_3$) δ 8.04 – 7.97 (m, 2H), 7.34 – 7.29 (m, 4H), 7.21 – 7.10 (m, 3H), 3.54 (dd, J = 18.2, 10.9 Hz, 1H), 3.41 (dd, J = 18.2, 5.0 Hz, 1H), 2.82 (dd, J = 10.9, 5.0 Hz, 1H), 1.27 (s, 6H), 1.19 (s, 6H). ^{13}C{^{1}H} NMR (151 MHz, $CDCl_3$) δ 198.14, 165.69 (d, J = 253.68 Hz), 141.81, 133.22 (d, J = 3.0 Hz), 130.67 (d, J = 9.3 Hz), 128.57, 128.38, 125.68, 115.58 (d, J = 22.65 Hz), 83.45, 43.16, 24.57, 24.53. ^{19}F NMR (471 MHz, $CDCl_3$) δ -

105.65. ^{11}B NMR (160 MHz, $CDCl_3$) δ 22.50. GC-MS (EI-70 eV): m/z (%) 354 (2), 339 (10), 296 (20), 254 (100), 210 (51), 123 (53).

1-(3-fluorophenyl)-3-phenyl-3-(4,4,5,5-tetramethyl-1,3,2-dioxaborolan-2-yl)propan-1-one

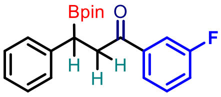

3ak

Purification by flash column chromatography (petroleum ether/EtOAc = 50:1). Colorless oil (26.9 mg, 76%). ^1H NMR (600 MHz, $CDCl_3$) δ 7.80 – 7.73 (m, 1H), 7.71 – 7.62 (m, 1H), 7.44 (td, J = 8.0, 5.5 Hz, 1H), 7.33 – 7.25 (m, 5H), 7.20 (ddd, J = 8.6, 5.8, 2.6 Hz, 1H), 3.55 (dd, J = 18.3, 10.9 Hz, 1H), 3.41 (dd, J = 18.3, 5.0 Hz, 1H), 2.83 (dd, J = 10.9, 4.9 Hz, 1H), 1.27 (s, 6H), 1.19 (s, 6H). $^{13}C\{^1H\}$ NMR (151 MHz, $CDCl_3$) δ 198.49 (d, J = 2.0 Hz), 162.84 (d, J = 247.64 Hz), 141.68, 138.88 (d, J = 6.1 Hz), 130.17 (d, J = 7.6 Hz), 128.59, 128.37, 125.72, 123.83 (d, J = 2.9 Hz), 119.95 (d, J = 21.14 Hz), 114.79 (d, J = 21.24 Hz), 83.50, 43.37, 24.57, 24.53. ^{19}F NMR (471 MHz, $CDCl_3$) δ -112.08. ^{11}B NMR (160 MHz, $CDCl_3$) δ 22.54. GC-MS (EI-70 eV): m/z (%) 354 (3), 296 (23), 254 (100), 210 (50), 123 (48). HRMS (ESI): calcd for $C_{21}H_{24}BFO_3$ $[M+Na]^+$: 377.1695, found: 377.1699.

1-(4-chlorophenyl)-3-phenyl-3-(4,4,5,5-tetramethyl-1,3,2-dioxaborolan-2-yl)propan-1-one[1]

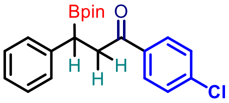

3al

Purification by flash column chromatography (petroleum ether/EtOAc = 50:1). White solid (27.0 mg, 73%). ^1H NMR (600 MHz, $CDCl_3$) δ 7.93 – 7.91 (m, 2H), 7.46 – 7.40 (m, 3H), 7.30 (dd, J = 5.9, 2.5 Hz, 3H), 7.19 (ddd, J = 8.6, 5.8, 2.6 Hz, 1H), 3.54 (dd, J = 18.2, 10.9 Hz, 1H), 3.41 – 3.35 (m, 1H), 2.82 (dd, J = 10.9, 5.0 Hz, 1H), 1.26 (s, 6H), 1.18 (s, 6H). $^{13}C\{^1H\}$ NMR (151 MHz, $CDCl_3$) δ 198.54, 141.72, 139.36, 135.09, 129.49, 128.82, 128.57, 128.37, 125.70, 83.48, 43.19, 24.56, 24.52. ^{11}B NMR (160 MHz, $CDCl_3$) δ 22.53. GC-MS (EI-70 eV): m/z (%) 370 (1), 312 (20), 287 (50), 270 (78), 243 (40), 139 (48), 84 (100).

1-(2-chlorophenyl)-3-phenyl-3-(4,4,5,5-tetramethyl-1,3,2-dioxaborolan-2-yl)propan-1-one

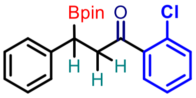

3am

Purification by flash column chromatography (petroleum ether/EtOAc = 50:1). Colorless oil (22.5 mg, 61%). ^1H NMR (600 MHz, $CDCl_3$) δ 7.96 (t, J = 1.8 Hz, 1H), 7.88 – 7.83 (m, 1H), 7.54 (ddd, J = 8.0, 2.1, 1.0 Hz, 1H), 7.41 (t, J = 7.9 Hz, 1H), 7.33 – 7.29 (m, 4H), 7.20 (ddd, J = 8.6, 5.8, 2.9 Hz, 1H), 3.55 (dd, J = 18.3, 10.9 Hz, 1H), 3.40 (dd, J = 18.3, 5.0 Hz, 1H), 2.83 (dd, J = 10.9, 4.9 Hz, 1H), 1.27 (s, 6H), 1.19 (s, 6H). $^{13}C\{^1H\}$ NMR (151 MHz, $CDCl_3$) δ 198.45, 141.67, 138.32, 134.86, 132.88, 129.86, 128.60, 128.38, 128.20, 126.15, 125.73, 83.51, 43.35, 24.57, 24.54. ^{11}B NMR (160 MHz, $CDCl_3$) δ 22.40. GC-MS (EI-70 eV): m/z (%) 370 (1), 312 (20), 287 (28), 243 (42), 139 (32), 84 (100). HRMS (ESI): calcd for $C_{21}H_{24}BClO_3$ $[M+Na]^+$: 393.1399, found: 393.1392.

1-(4-bromophenyl)-3-phenyl-3-(4,4,5,5-tetramethyl-1,3,2-dioxaborolan-2-yl)propan-1-one

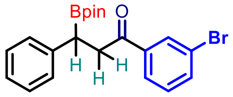

3an

Purification by flash column chromatography (petroleum ether/EtOAc = 50:1). Colorless oil (24.4 mg, 59%). ^{1}H NMR (600 MHz, $CDCl_3$) δ 8.11 (s, 1H), 7.90 (d, *J* = 7.8 Hz, 1H), 7.69 (d, *J* = 7.9 Hz, 1H), 7.36 – 7.27 (m, 6H), 3.54 (dd, *J* = 18.3, 10.8 Hz, 1H), 3.39 (dd, *J* = 18.3, 4.9 Hz, 1H), 2.82 (dd, *J* = 10.8, 4.8 Hz, 1H), 1.27 (s, 6H), 1.19 (s, 6H). $^{13}C\{^{1}H\}$ NMR (151 MHz, $CDCl_3$) δ 198.36, 141.65, 138.51, 135.79, 131.15, 130.13, 128.60, 128.37, 126.59, 125.73, 122.90, 83.51, 43.32, 24.56, 24.53. ^{11}B NMR (160 MHz, $CDCl_3$) δ 22.54. GC-MS (EI-70 eV): m/z (%) 414 (1), 358 (20), 314 (52), 287 (25), 191 (40), 84 (100). HRMS (ESI): calcd for $C_{21}H_{24}BBrO_3$ $[M+Na]^+$: 437.0894, found: 437.0892.

4. References

(1) X. Huang, J. Hu, M. Wu, J. Wang, Y. Peng, G. Song, *Green Chem.*, 2018, **20**, 255-260.

(2) M. L. Shegavi, S. Saini, R. Bhawar, M. D. Vishwantha, S. K. Bose, *Adv. Synth. Catal.*, 2021, **363**, 2408-2416.

(3) L. Zhao, Y. Ma, F. He, W. Duan, J. Chen, C. Song, *J. Org. Chem.*, 2013, **78**, 1677-1681.

5. NMR Spectrum Copies

^{1}H NMR (600 MHz) Spectrum of **3aa** in $CDCl_3$

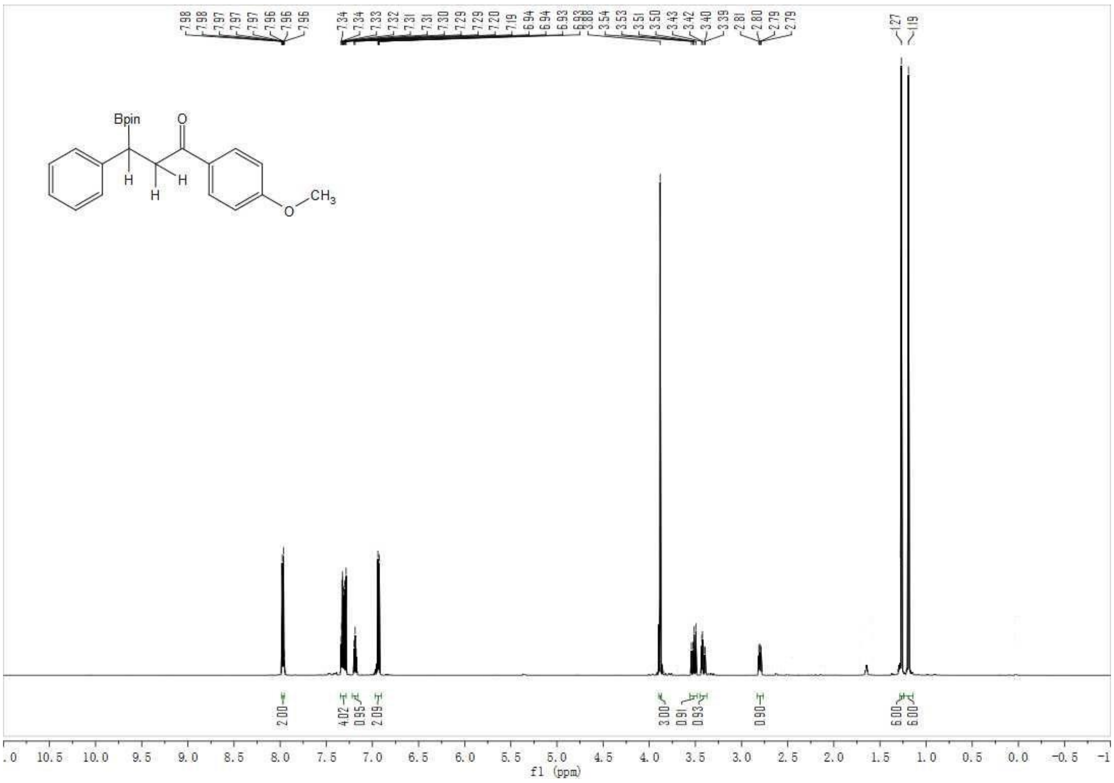

^{13}C{^{1}H} NMR (151 MHz) Spectrum of **3aa** in $CDCl_3$

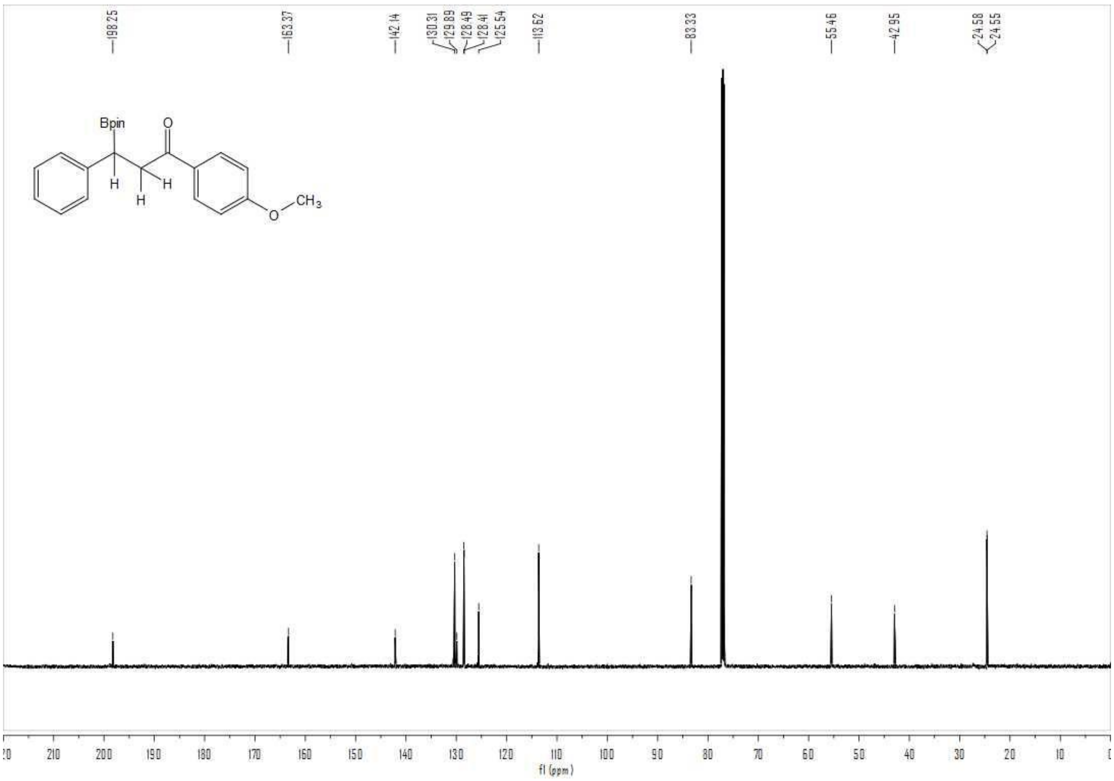

^{11}B NMR (160 MHz) Spectrum of **3aa** in $CDCl_3$

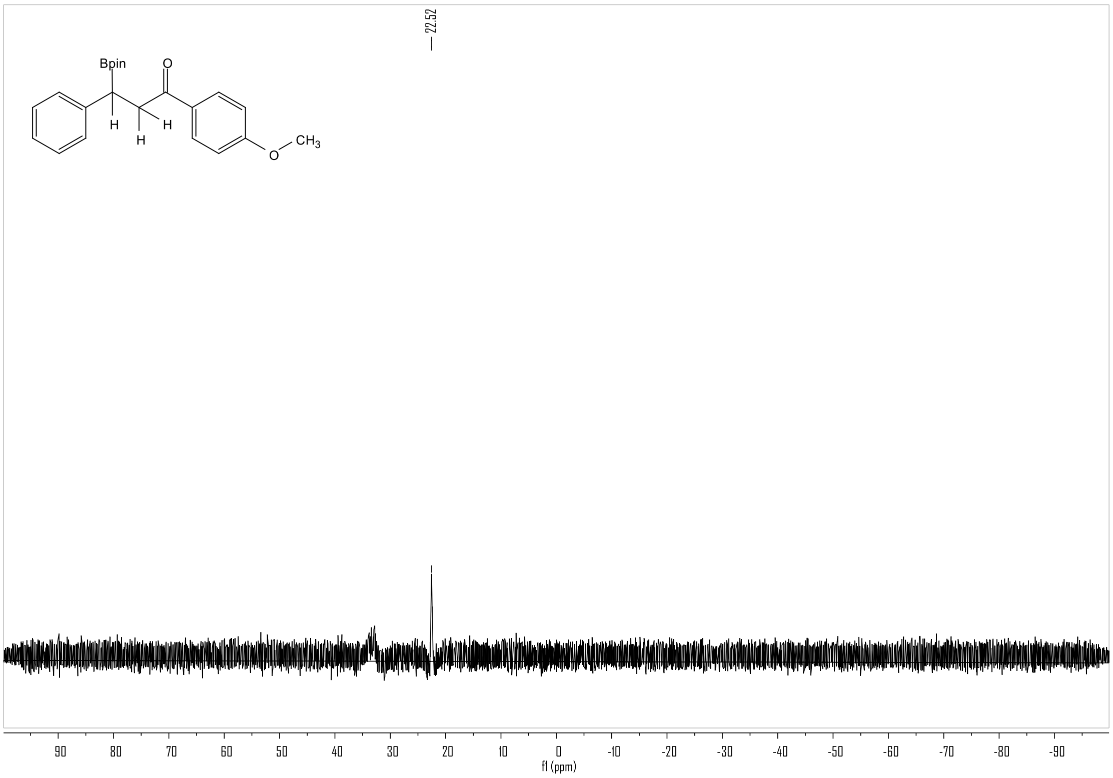

^{1}H NMR (600 MHz) Spectrum of **3ba** in $CDCl_3$

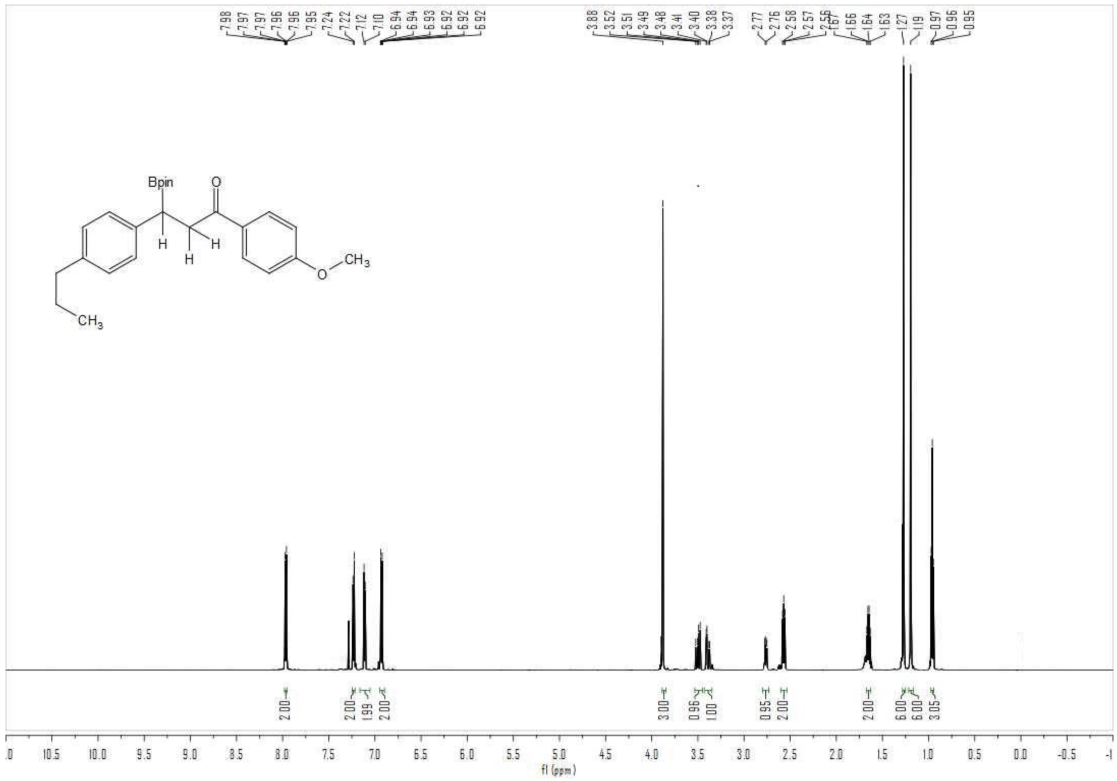

^{13}C{^{1}H} NMR (151 MHz) Spectrum of **3ba** in $CDCl_3$

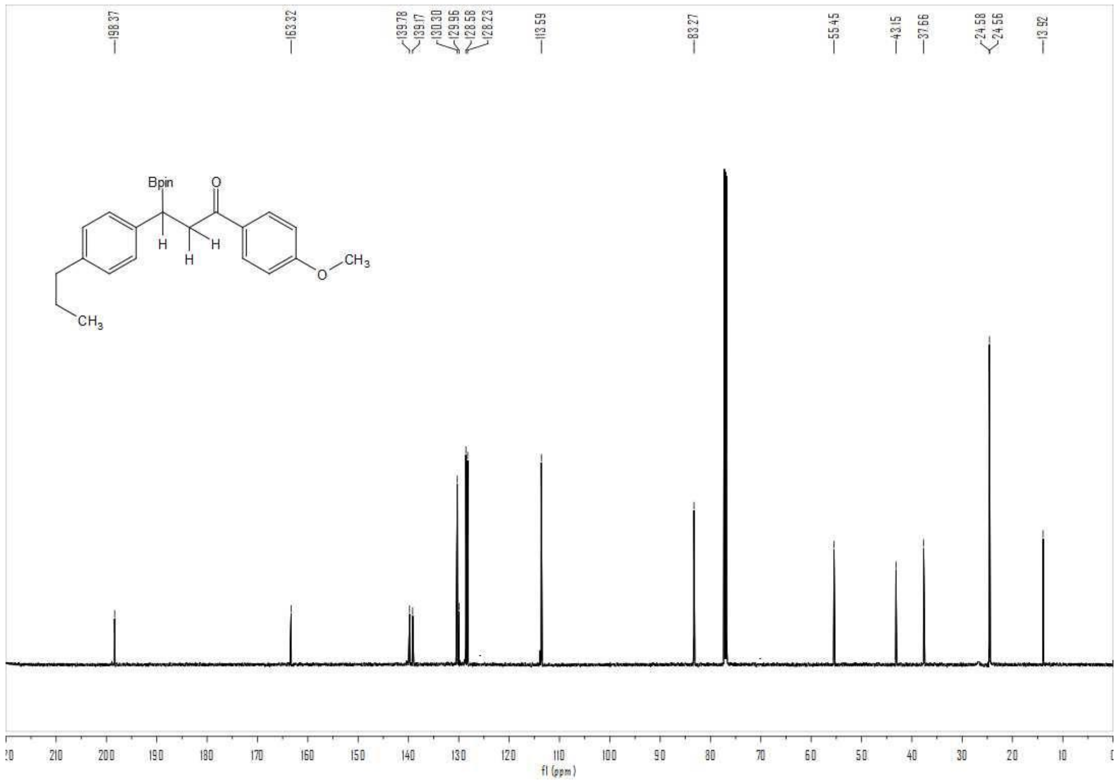

^{11}B NMR (160 MHz) Spectrum of **3ba** in $CDCl_3$

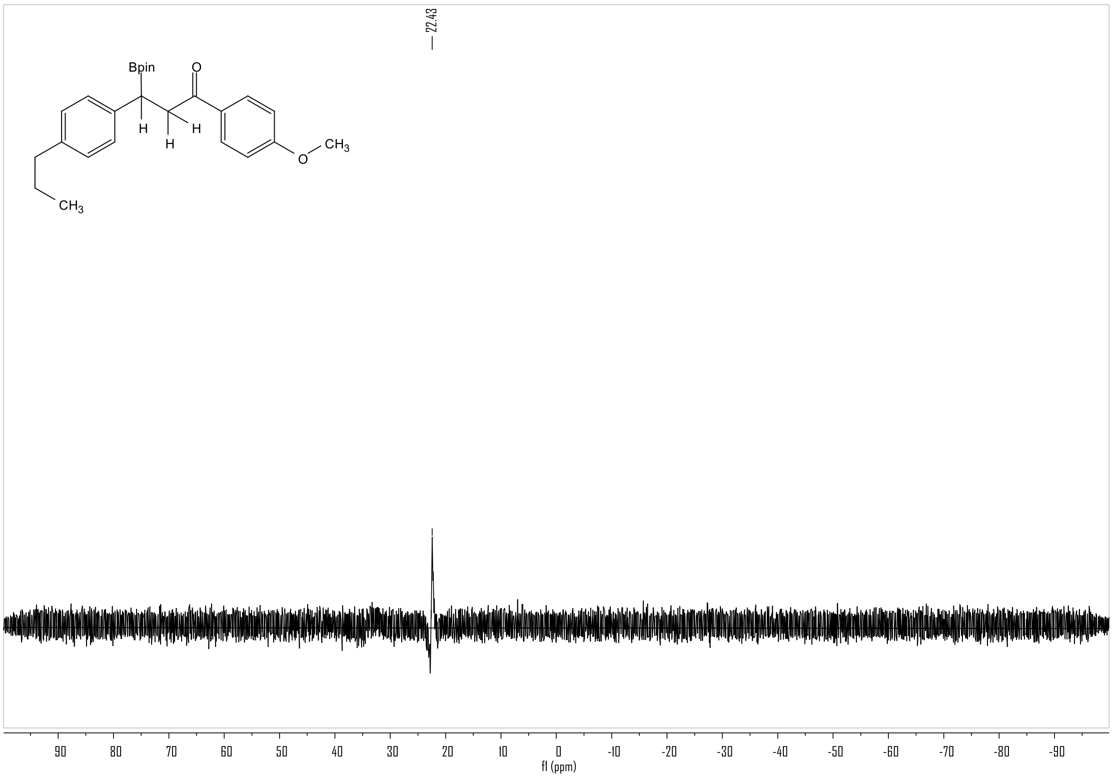

^1H NMR (600 MHz) Spectrum of **3ca** in $CDCl_3$

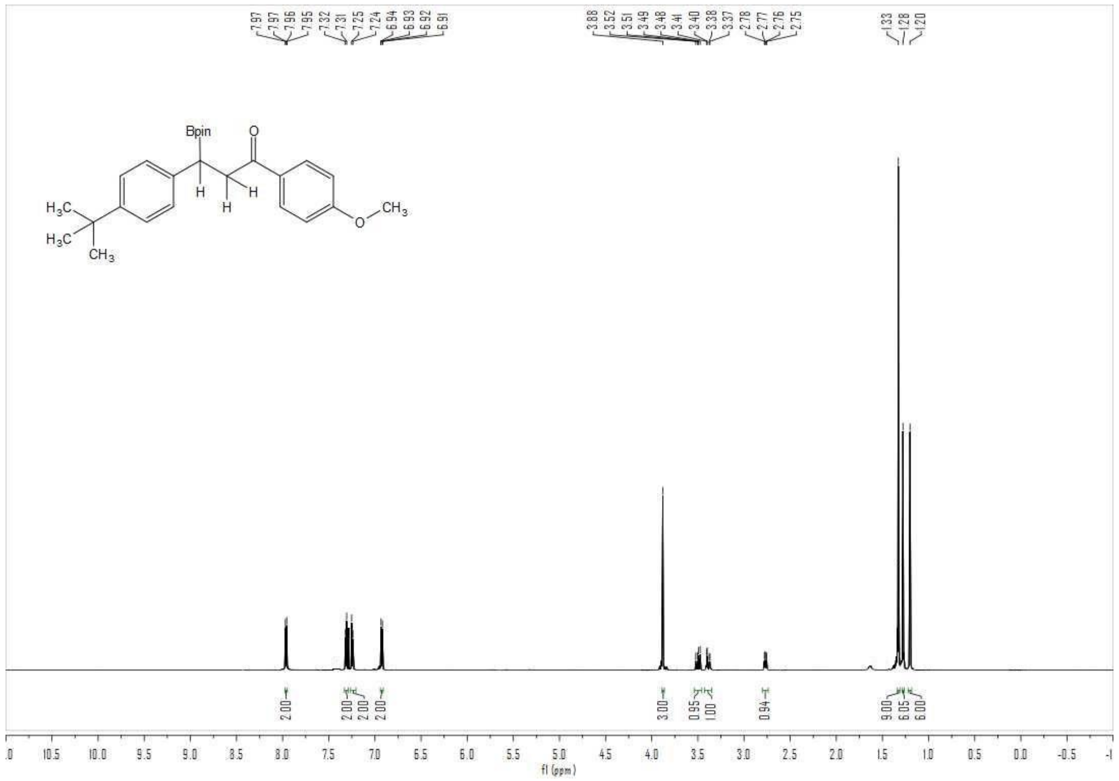

$^{13}C\{^1H\}$ NMR (151 MHz) Spectrum of **3ca** in $CDCl_3$

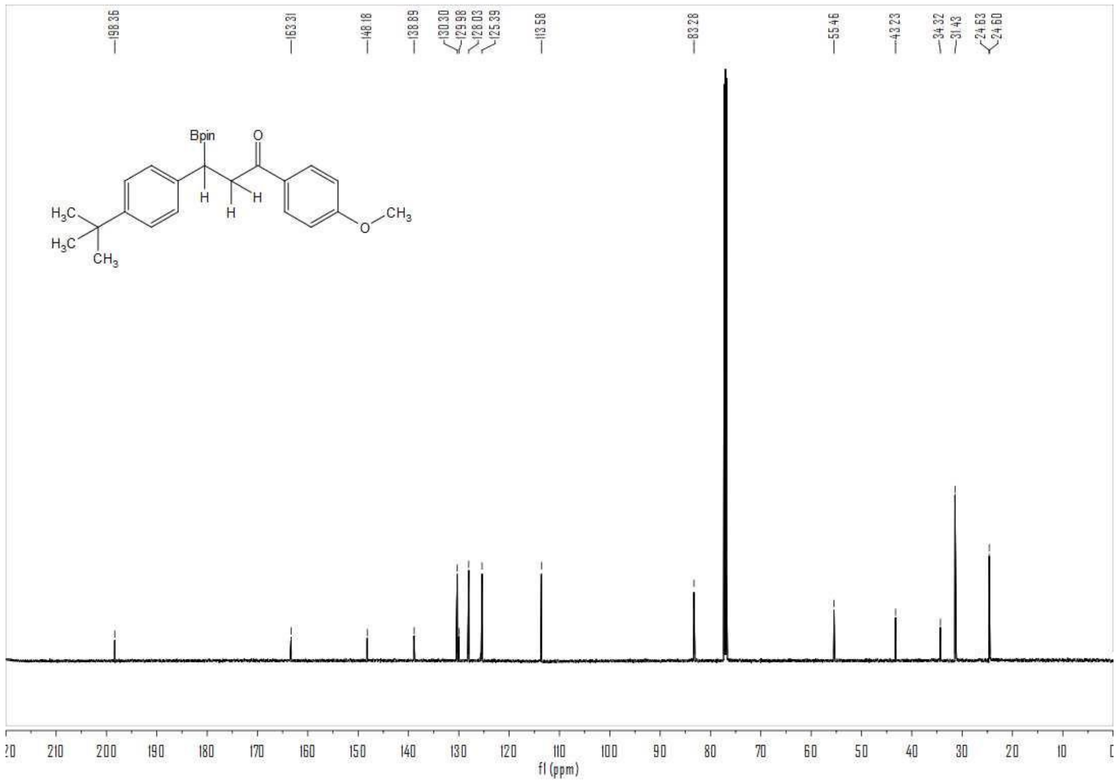

^{11}B NMR (160 MHz) Spectrum of **3ca** in $CDCl_3$

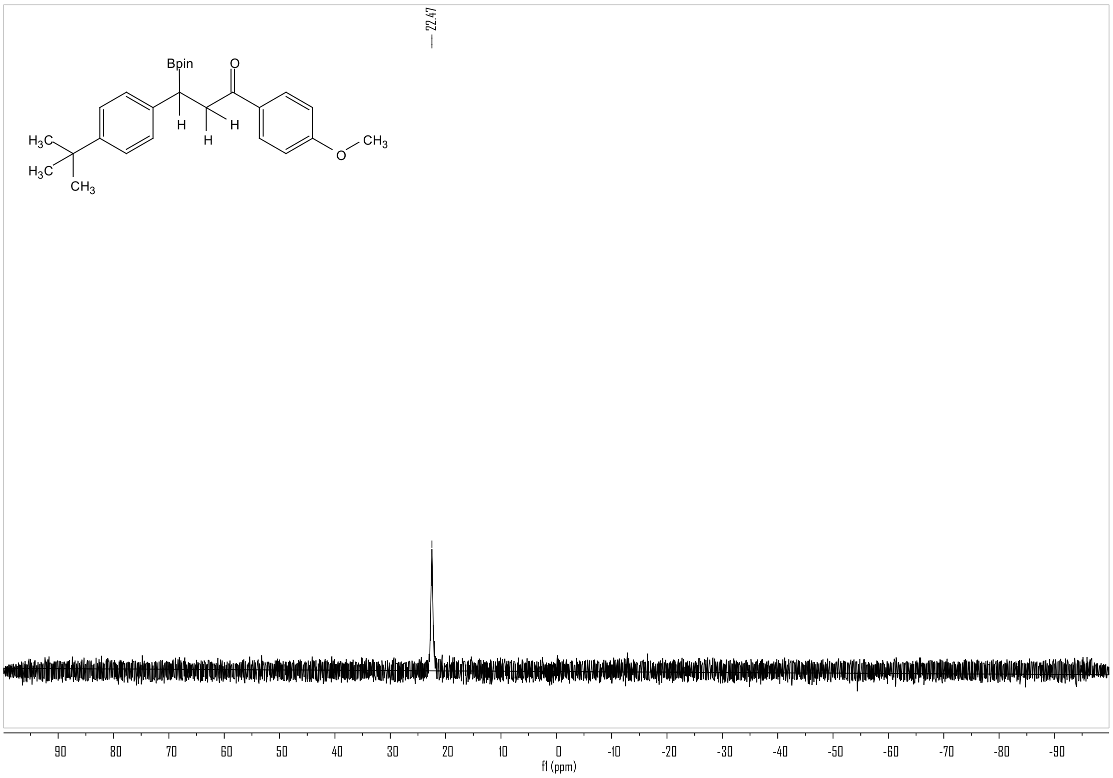

^{1}H NMR (600 MHz) Spectrum of **3da** in $CDCl_3$

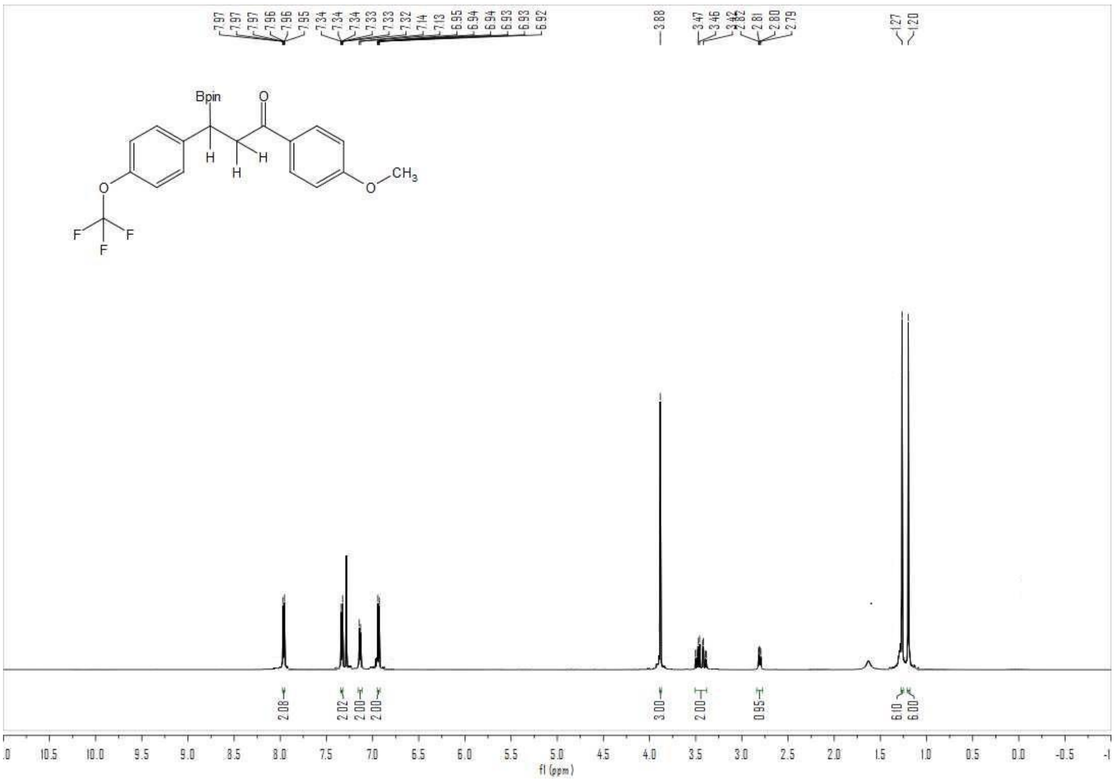

$^{13}C\{^{1}H\}$ NMR (151 MHz) Spectrum of **3da** in $CDCl_3$

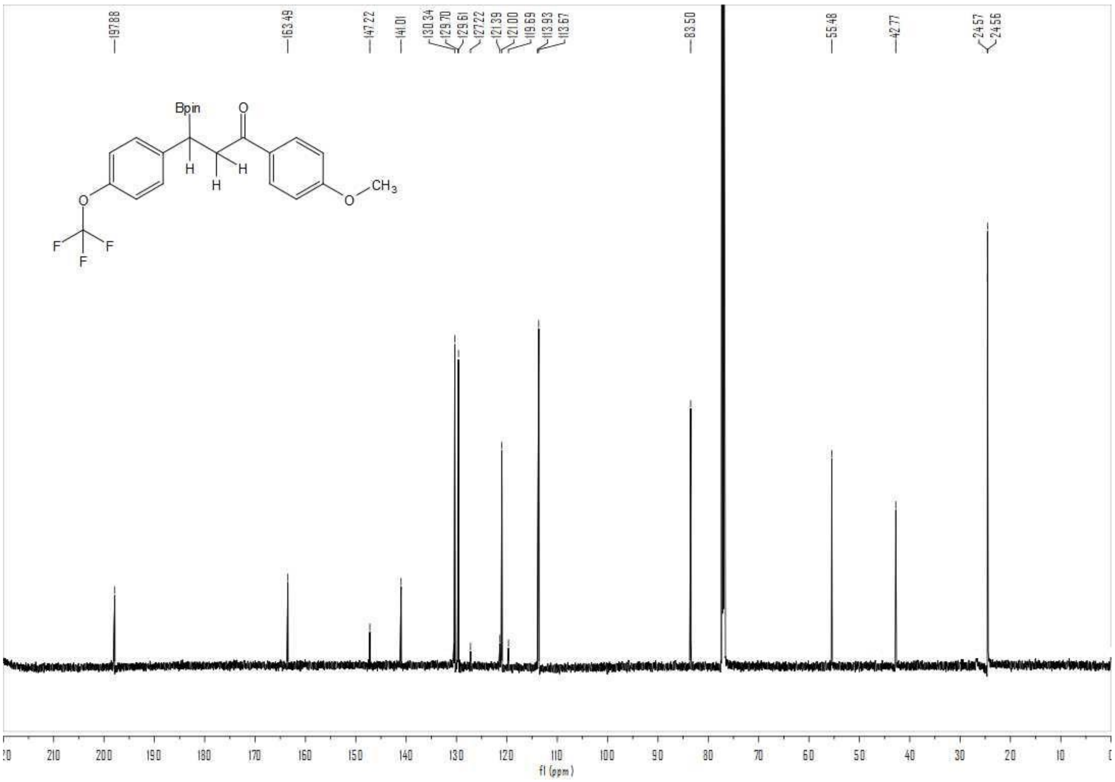

^{19}F NMR (471 MHz) Spectrum of **3da** in $CDCl_3$

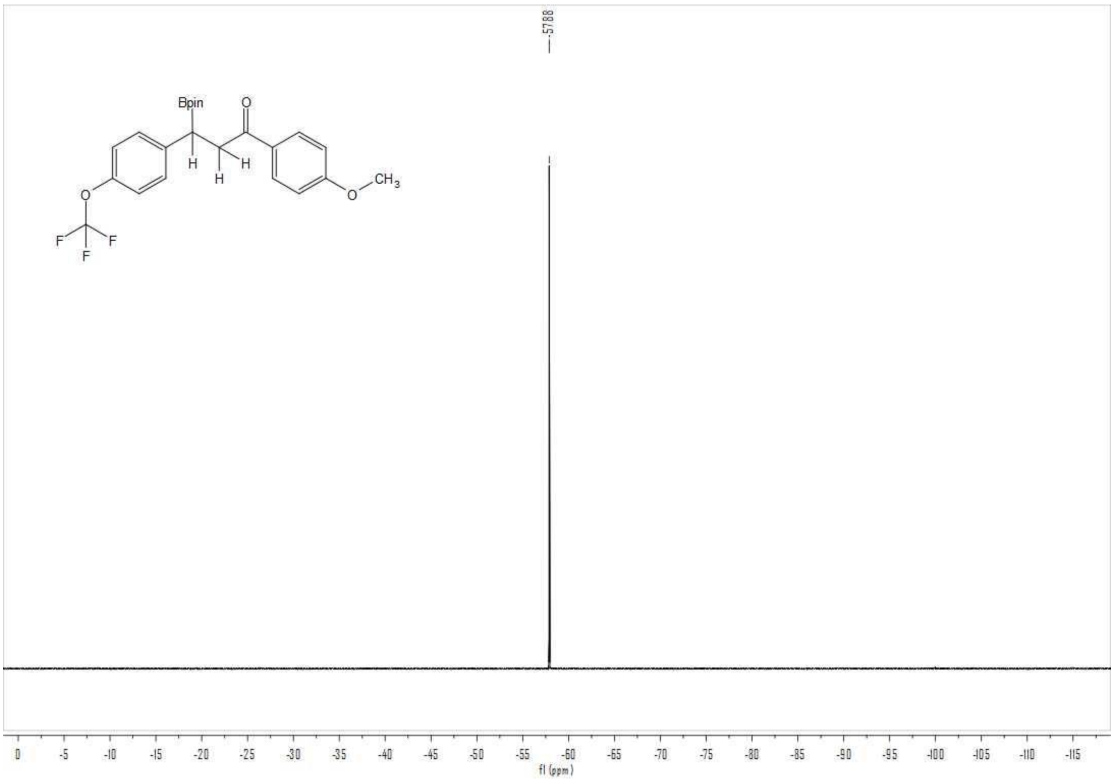

^{11}B NMR (160 MHz) Spectrum of **3da** in $CDCl_3$

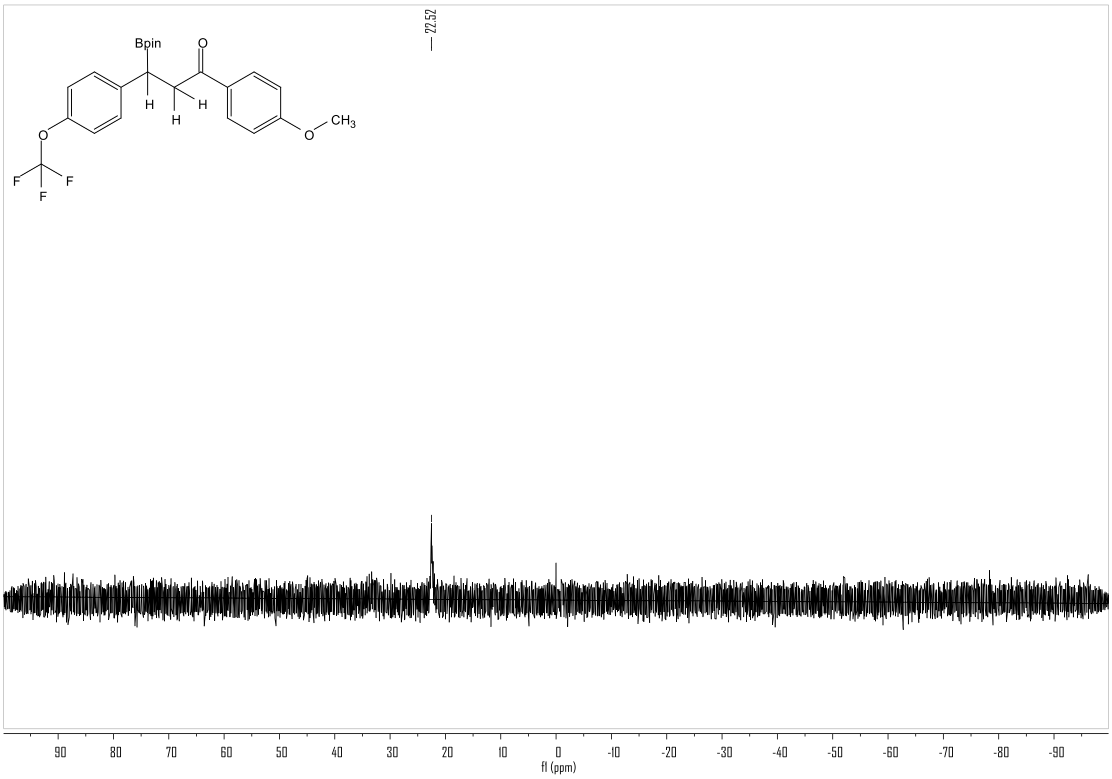

^{1}H NMR (600 MHz) Spectrum of **3ea** in $CDCl_3$

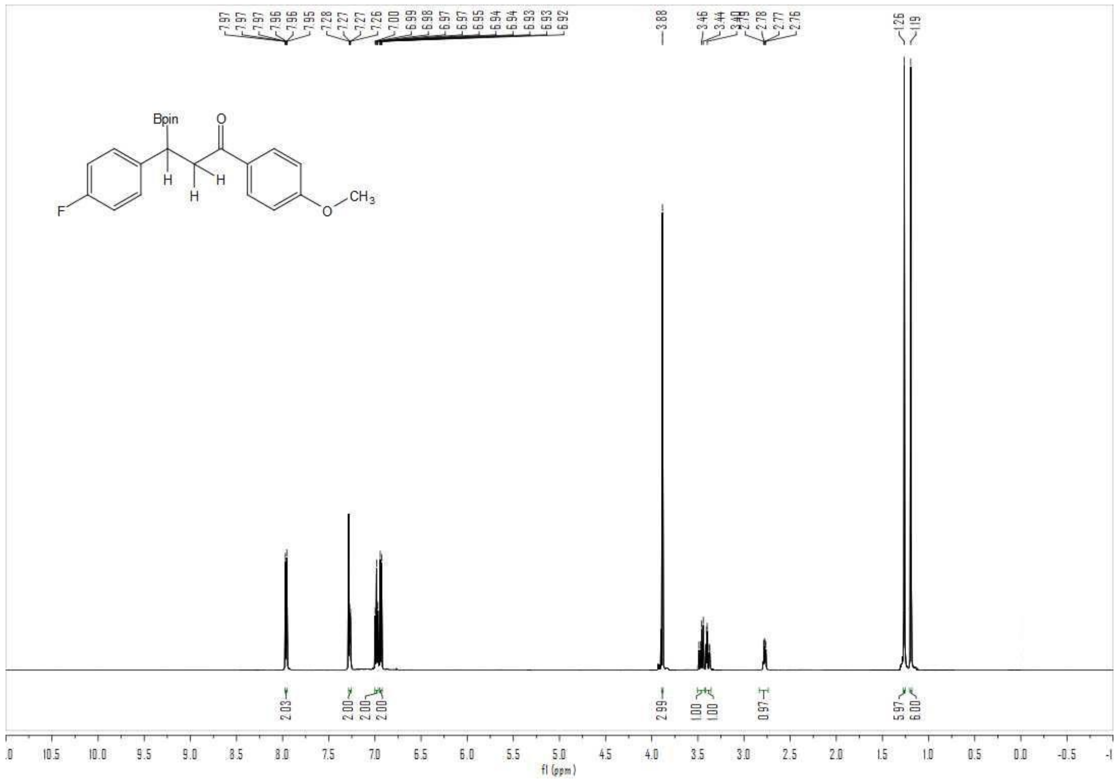

$^{13}C\{^{1}H\}$ NMR (151 MHz) Spectrum of **3ea** in $CDCl_3$

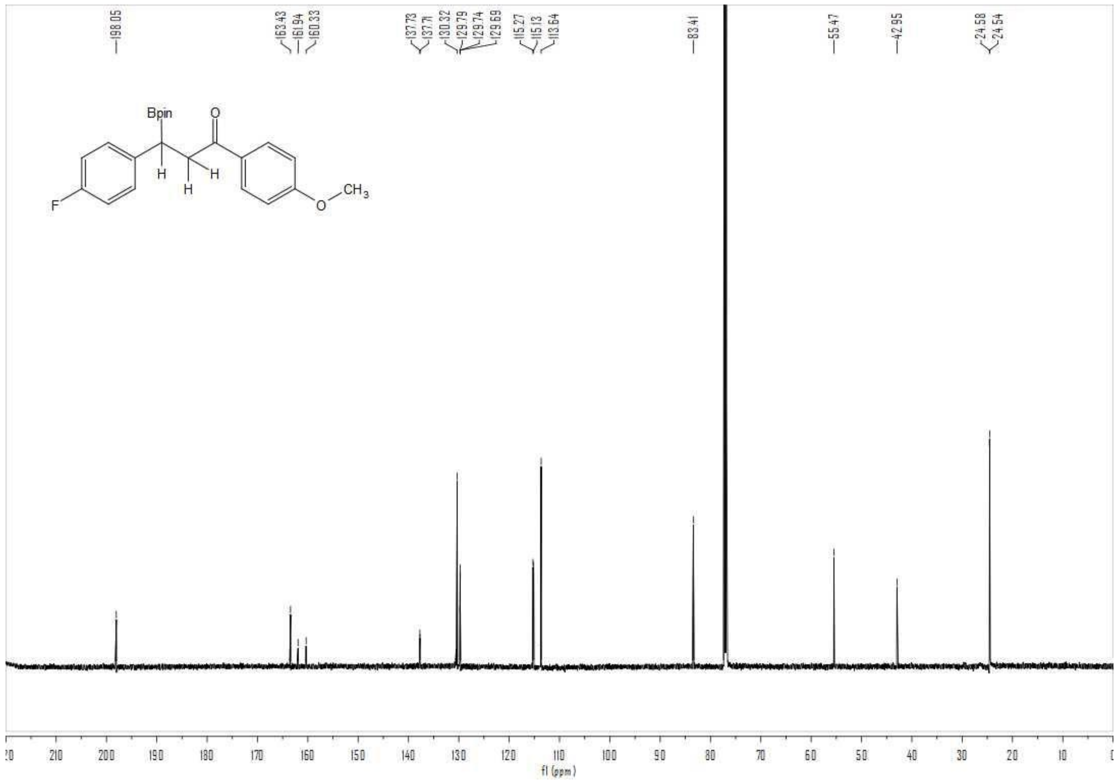

^{19}F NMR (471 MHz) Spectrum of **3ea** in $CDCl_3$

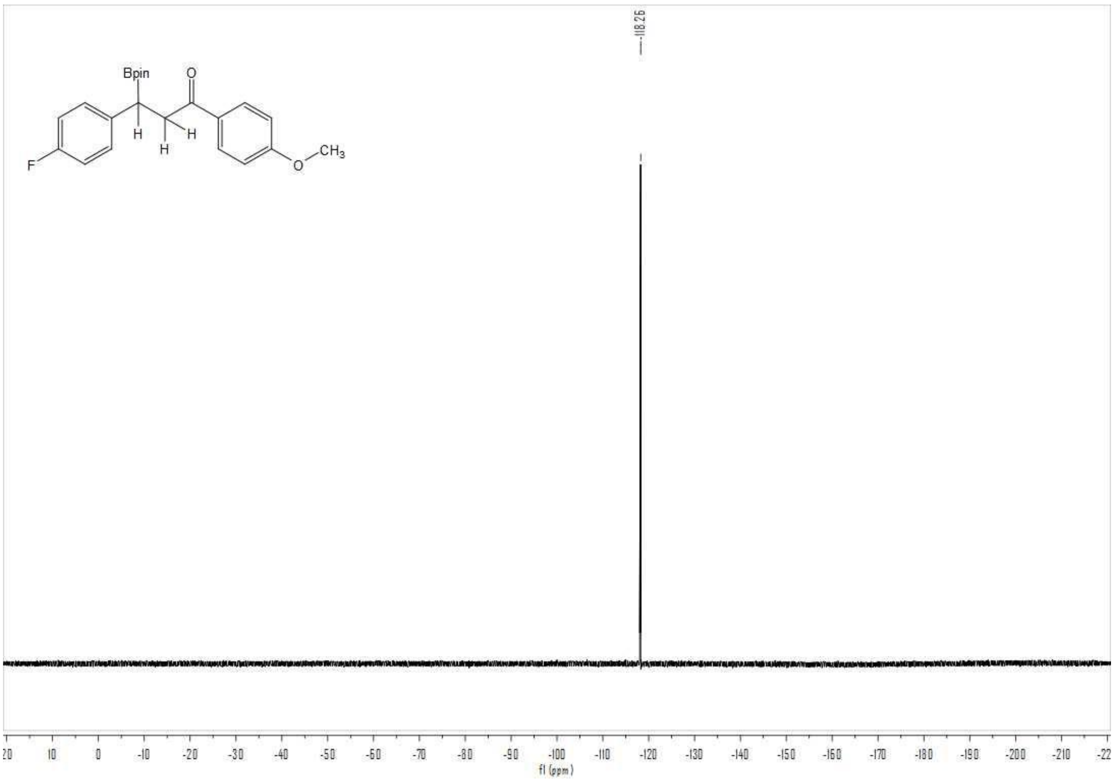

^{11}B NMR (160 MHz) Spectrum of **3ea** in $CDCl_3$

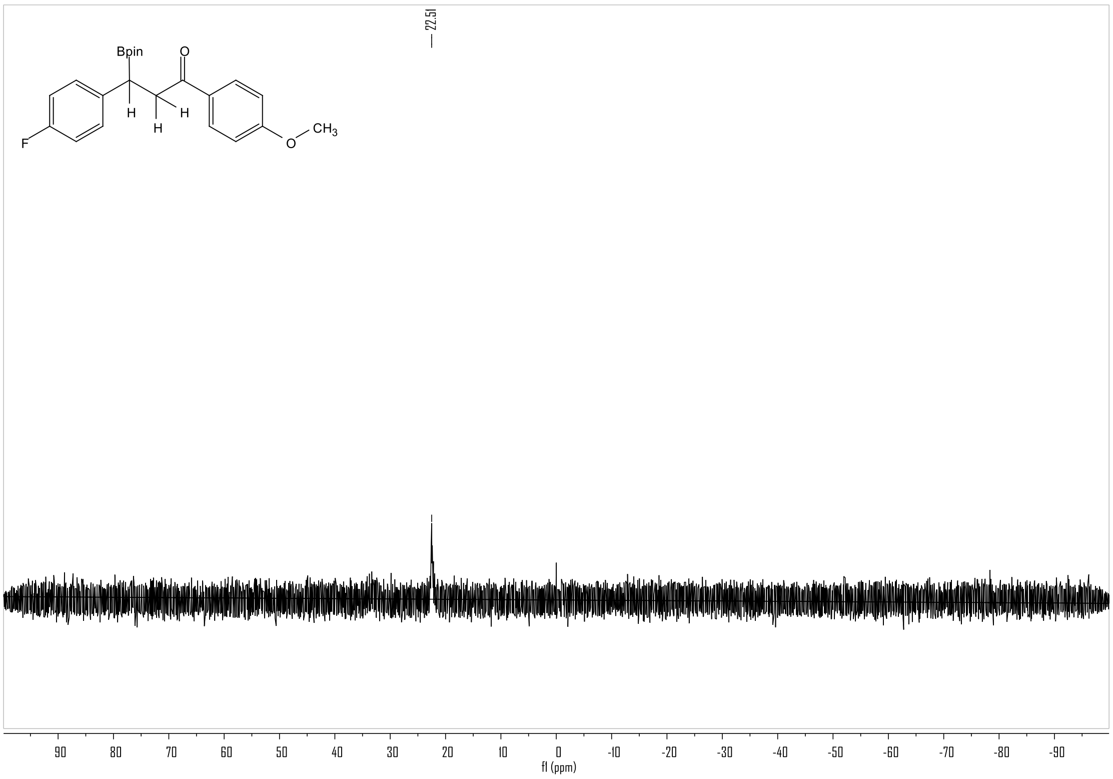

^{1}H NMR (600 MHz) Spectrum of **3fa** in $CDCl_3$

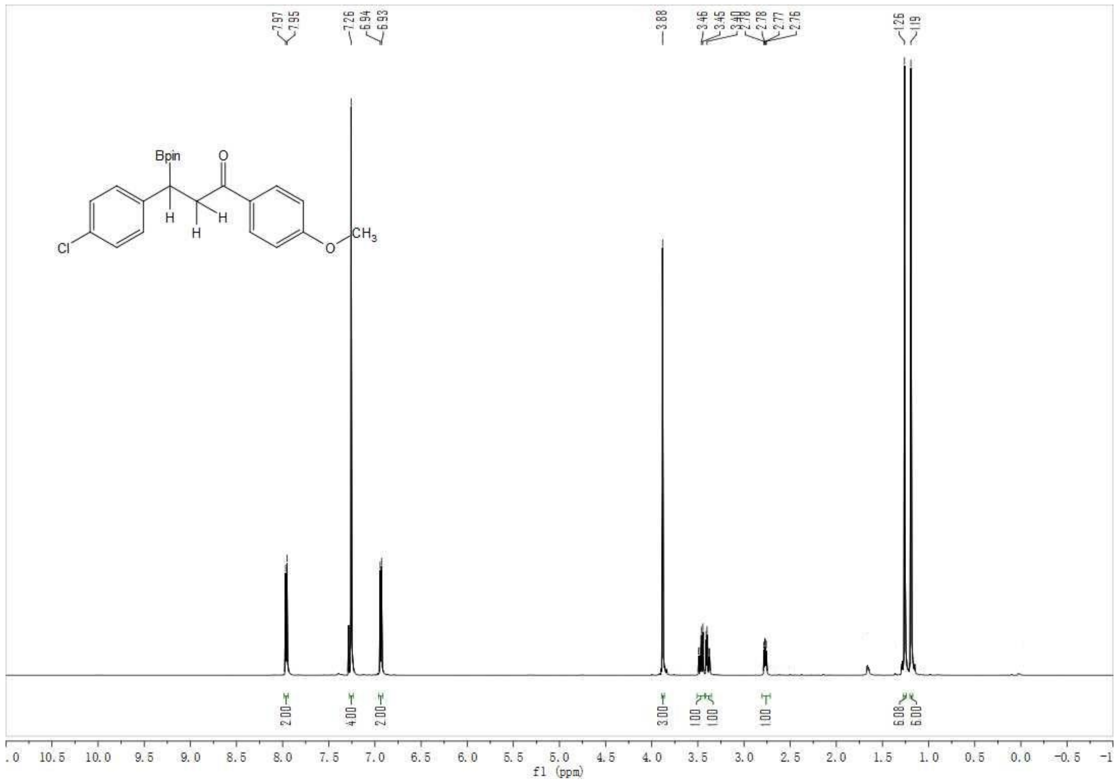

$^{13}C\{^{1}H\}$ NMR (151 MHz) Spectrum of **3fa** in $CDCl_3$

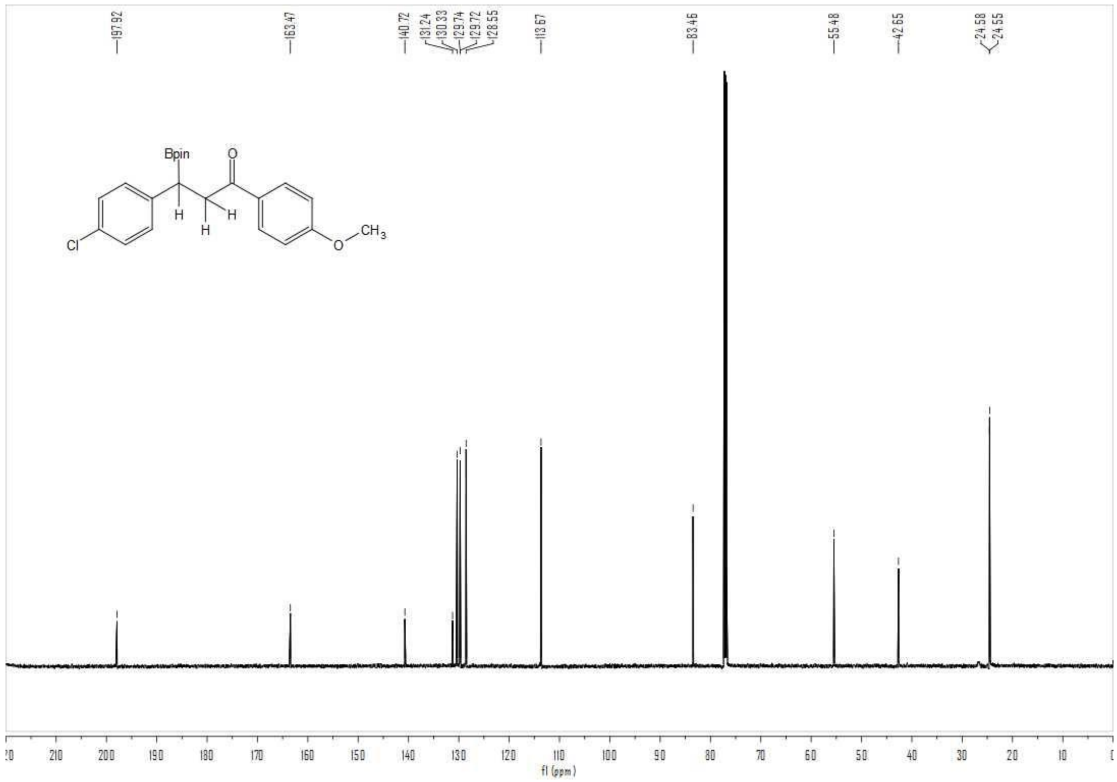

^{11}B NMR (160 MHz) Spectrum of **3fa** in $CDCl_3$

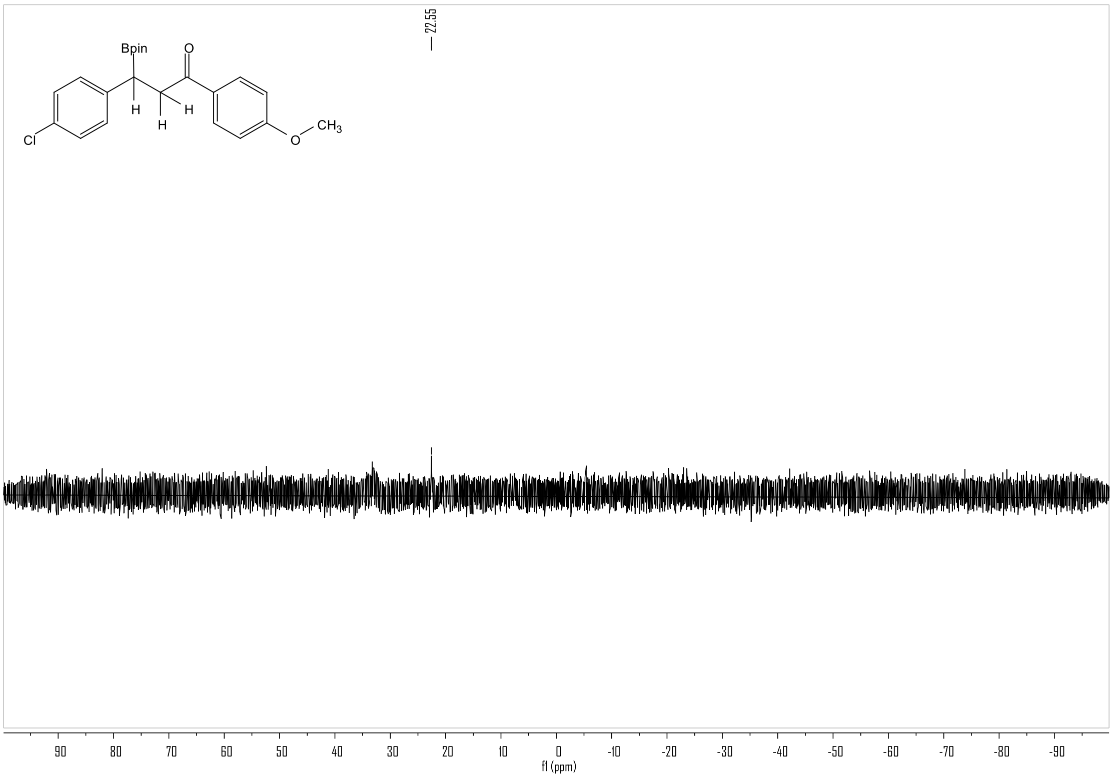

^{1}H NMR (600 MHz) Spectrum of **3ga** in $CDCl_3$

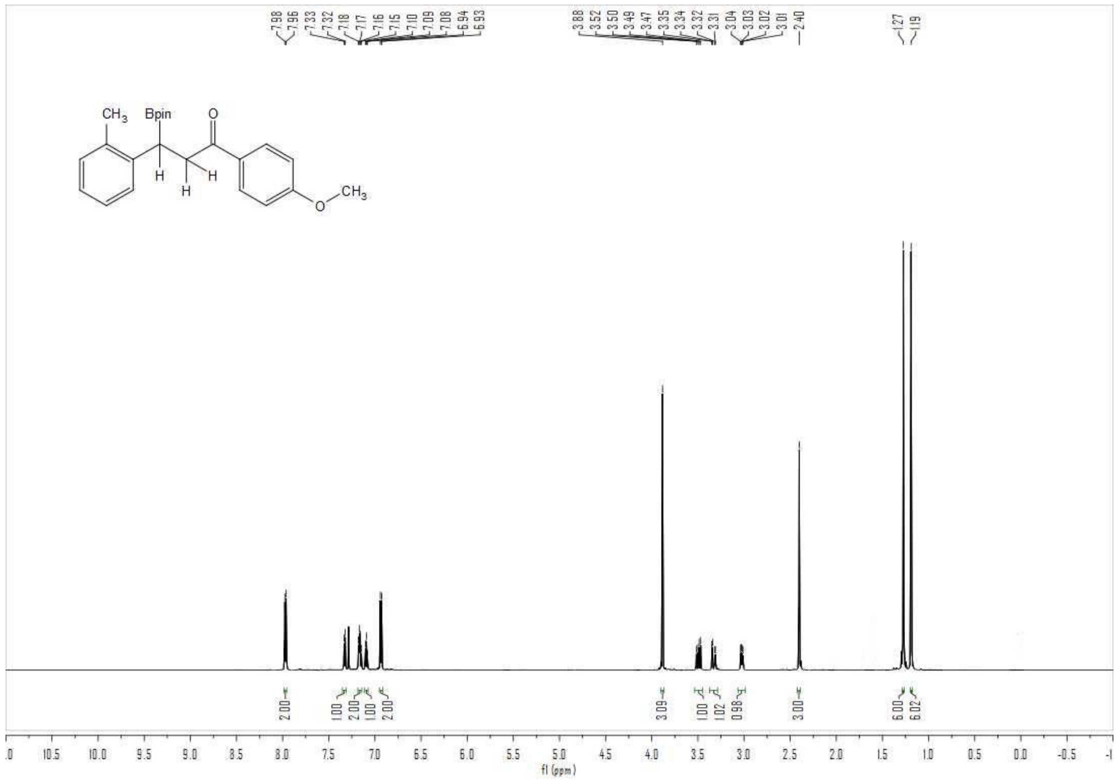

$^{13}C\{^{1}H\}$ NMR (151 MHz) Spectrum of **3ga** in $CDCl_3$

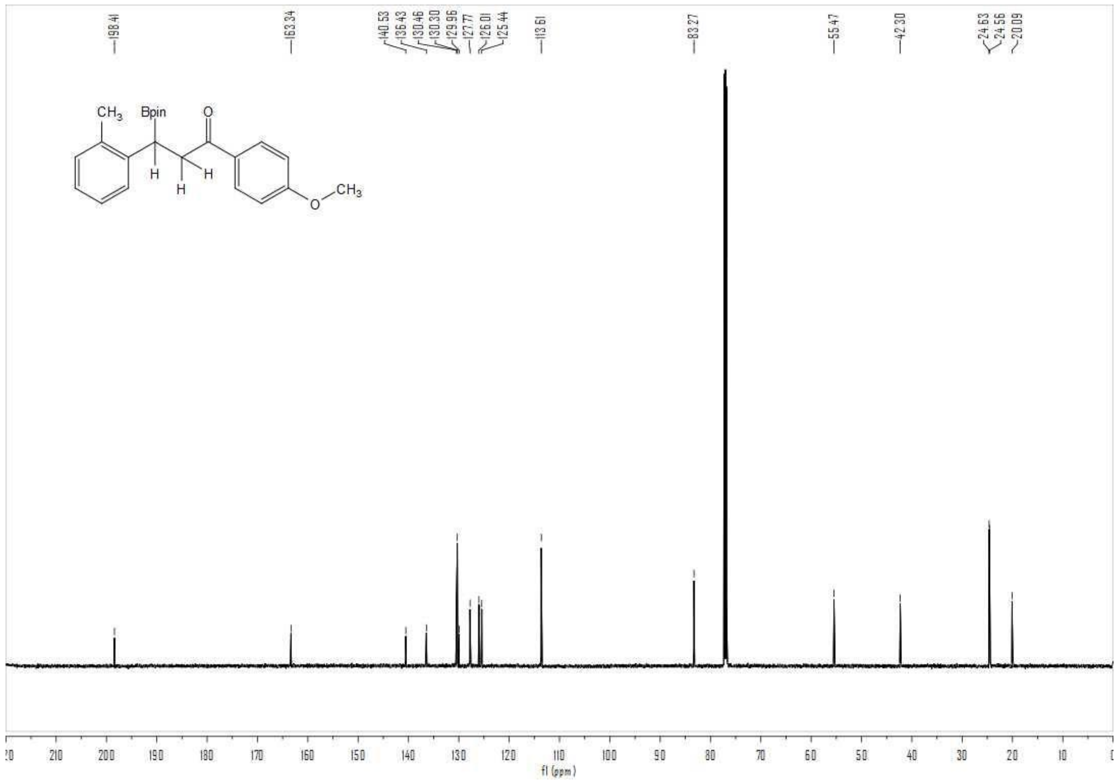

^{11}B NMR (160 MHz) Spectrum of **3ga** in $CDCl_3$

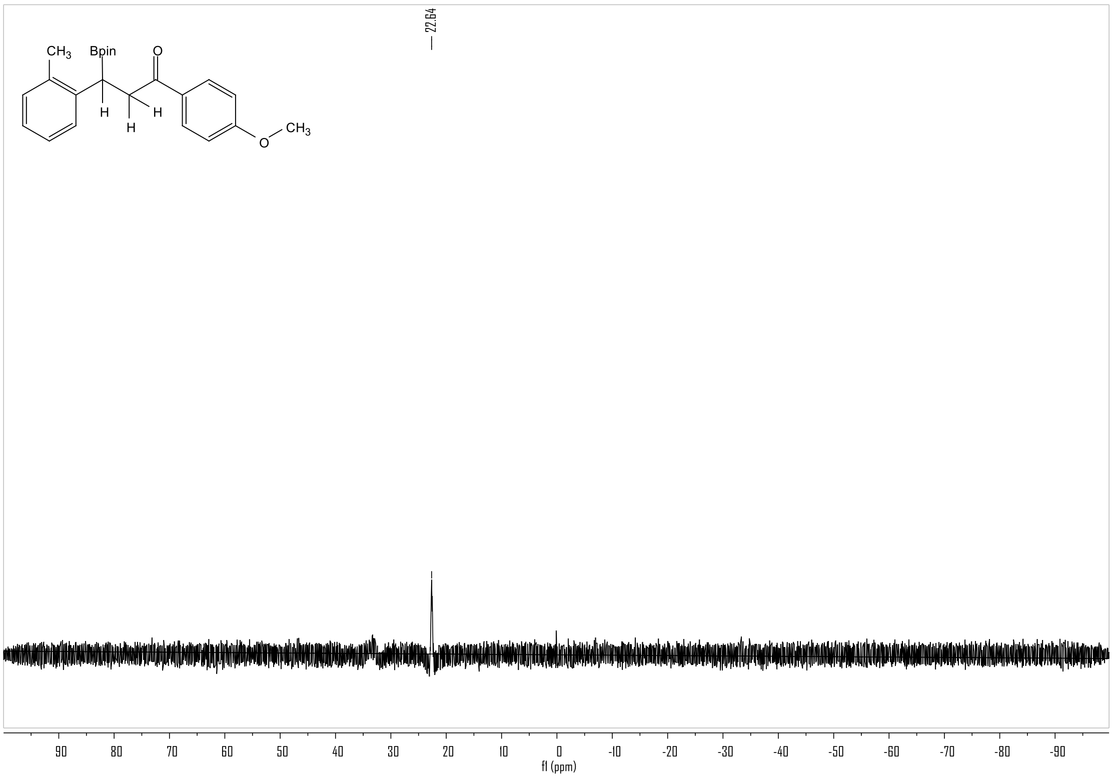

^{1}H NMR (600 MHz) Spectrum of **3ha** in $CDCl_3$

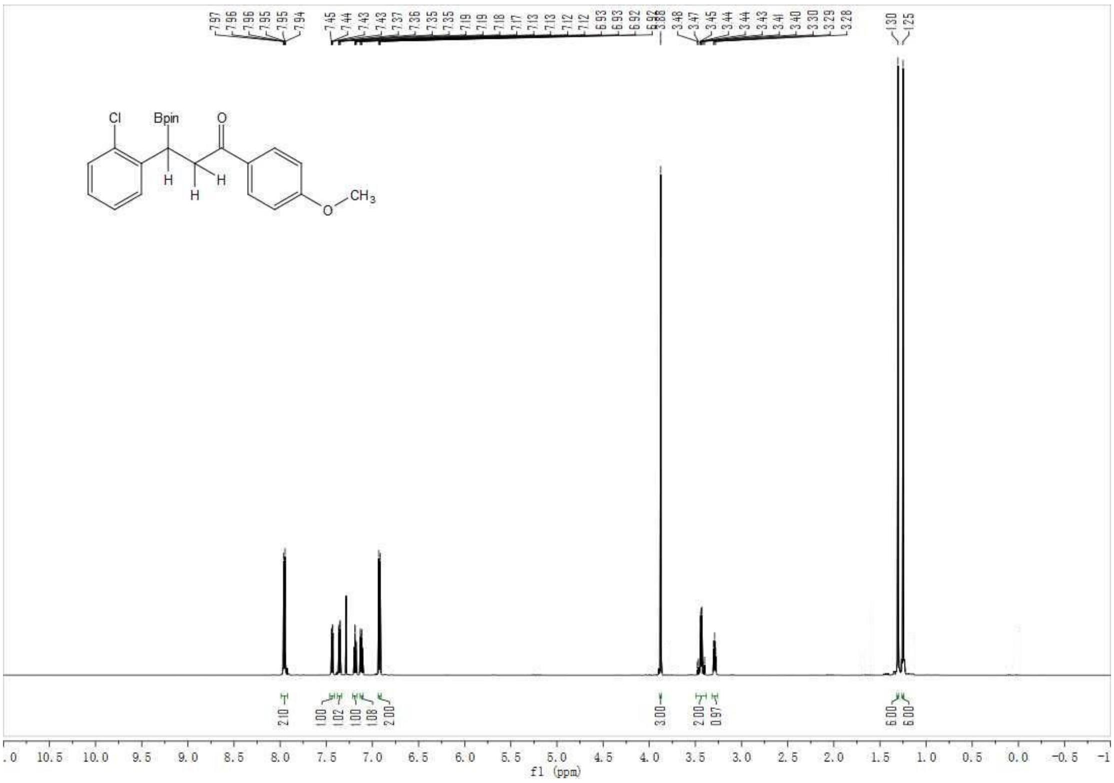

^{13}C{^{1}H} NMR (151 MHz) Spectrum of **3ha** in $CDCl_3$

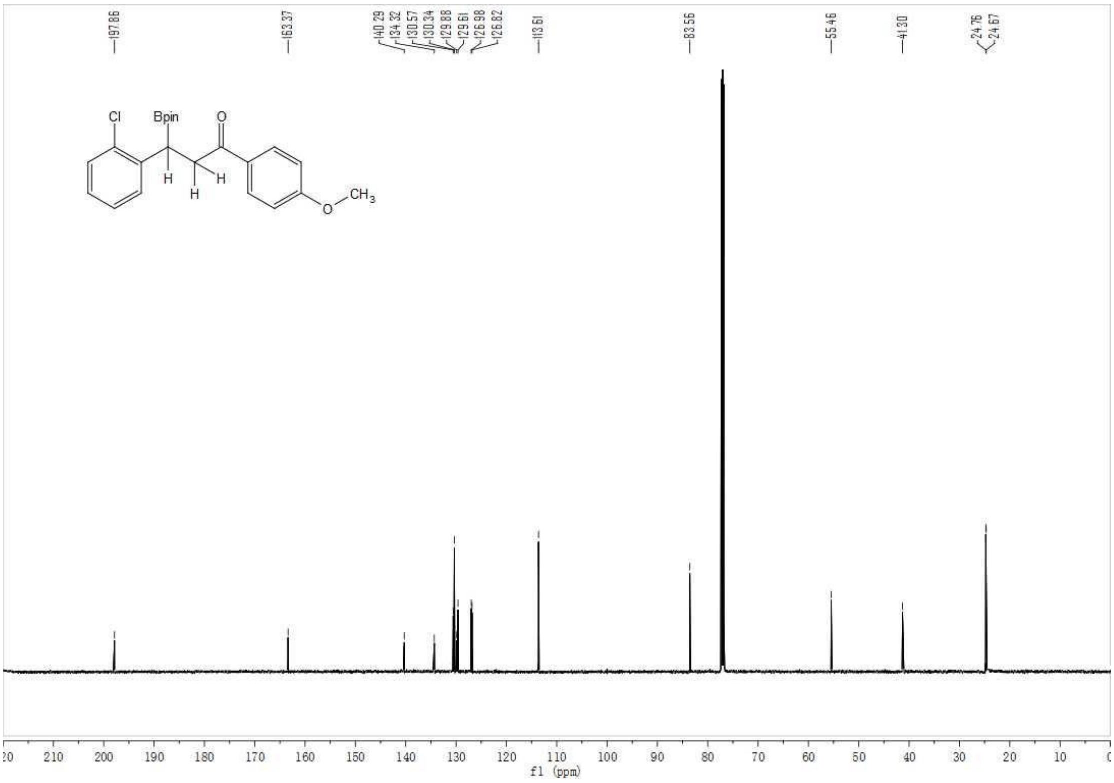

^{11}B NMR (160 MHz) Spectrum of **3ha** in $CDCl_3$

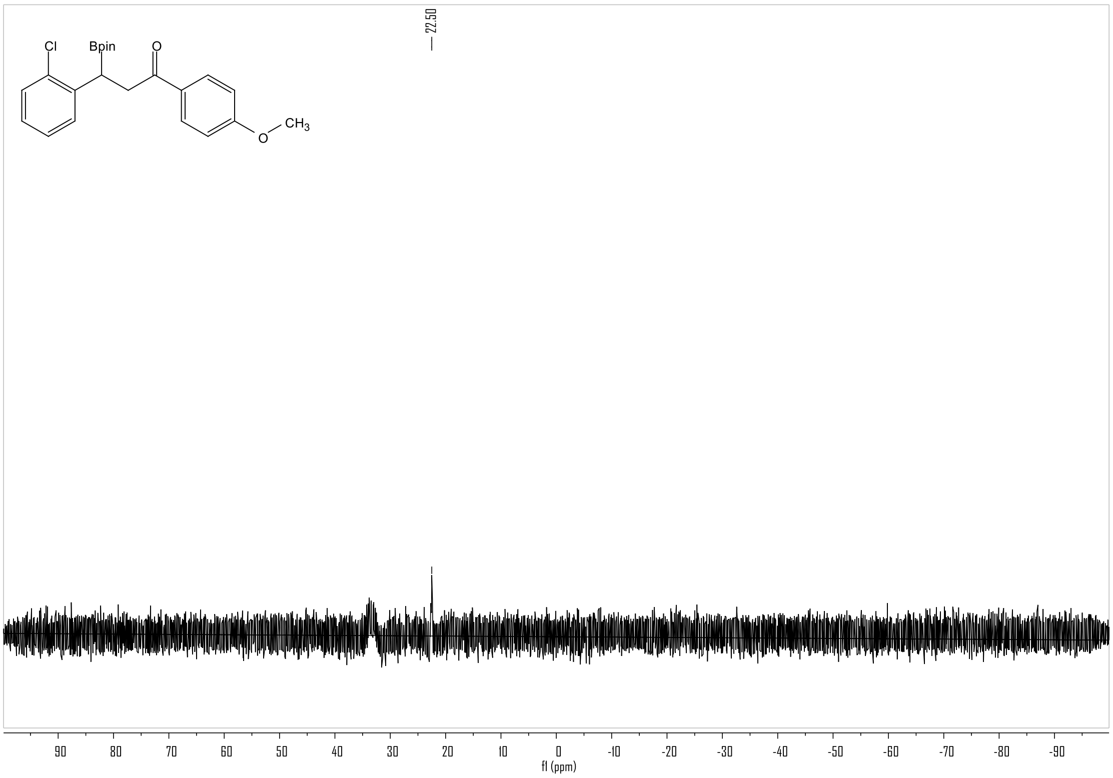

^{1}H NMR (600 MHz) Spectrum of **3ia** in $CDCl_3$

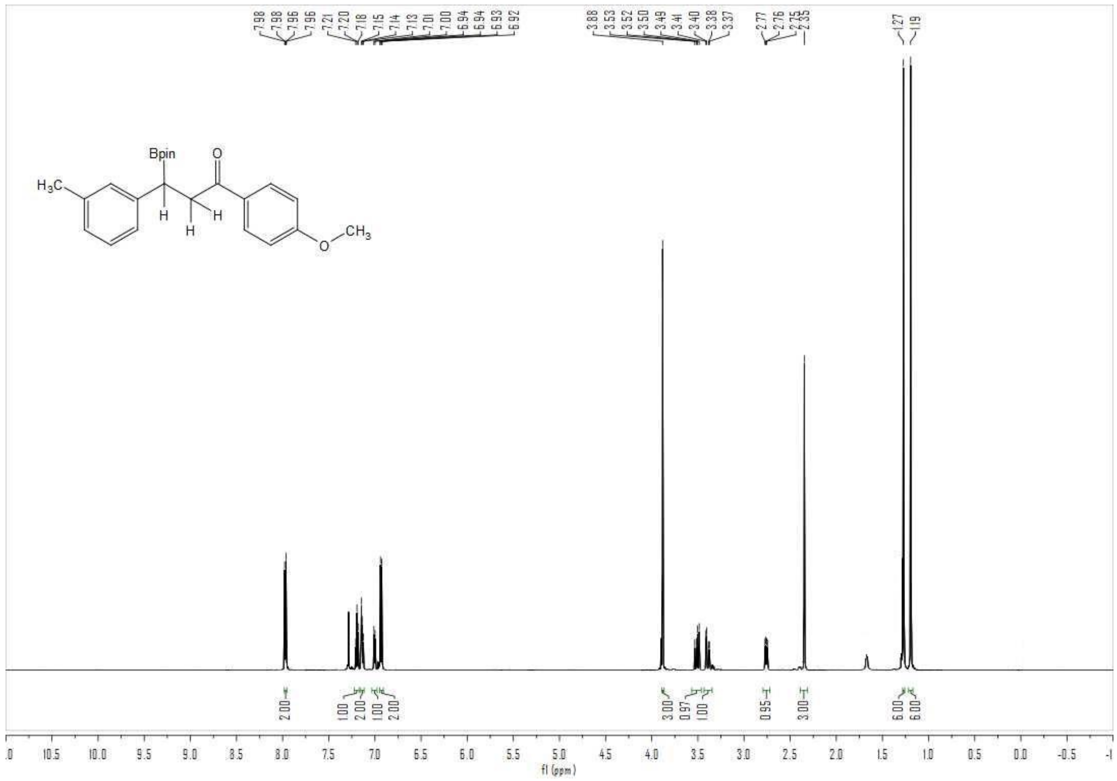

$^{13}C\{^{1}H\}$ NMR (151 MHz) Spectrum of **3ia** in $CDCl_3$

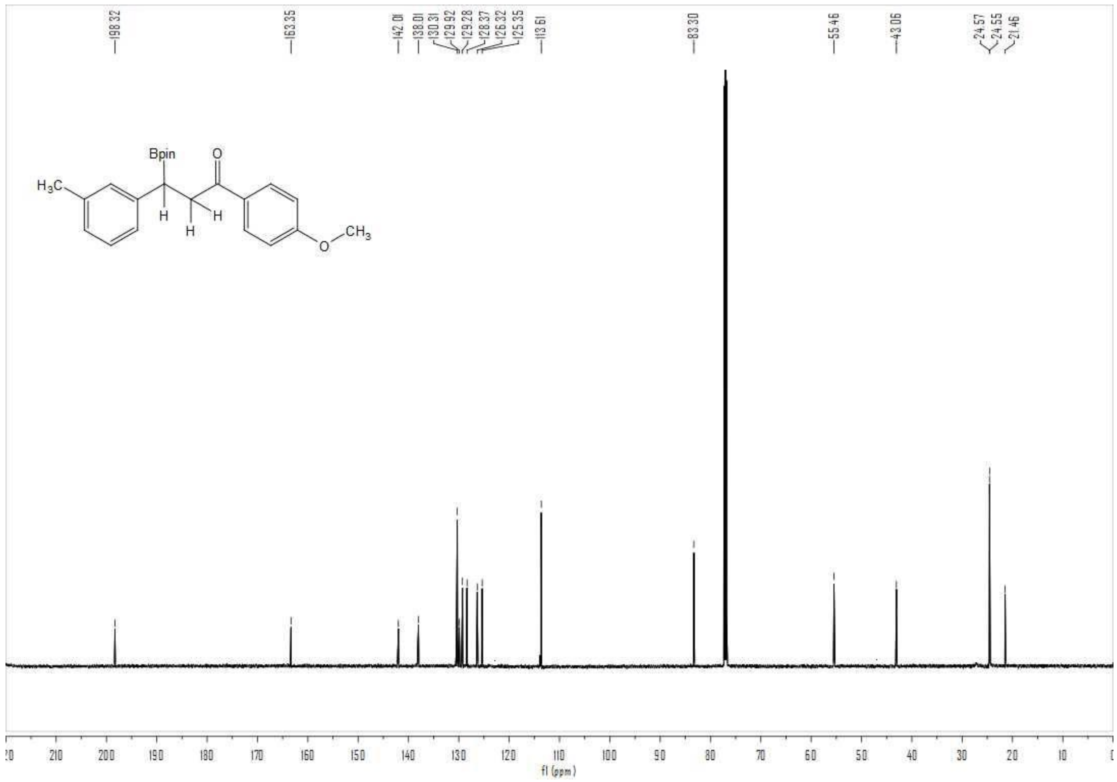

^{11}B NMR (160 MHz) Spectrum of **3ia** in $CDCl_3$

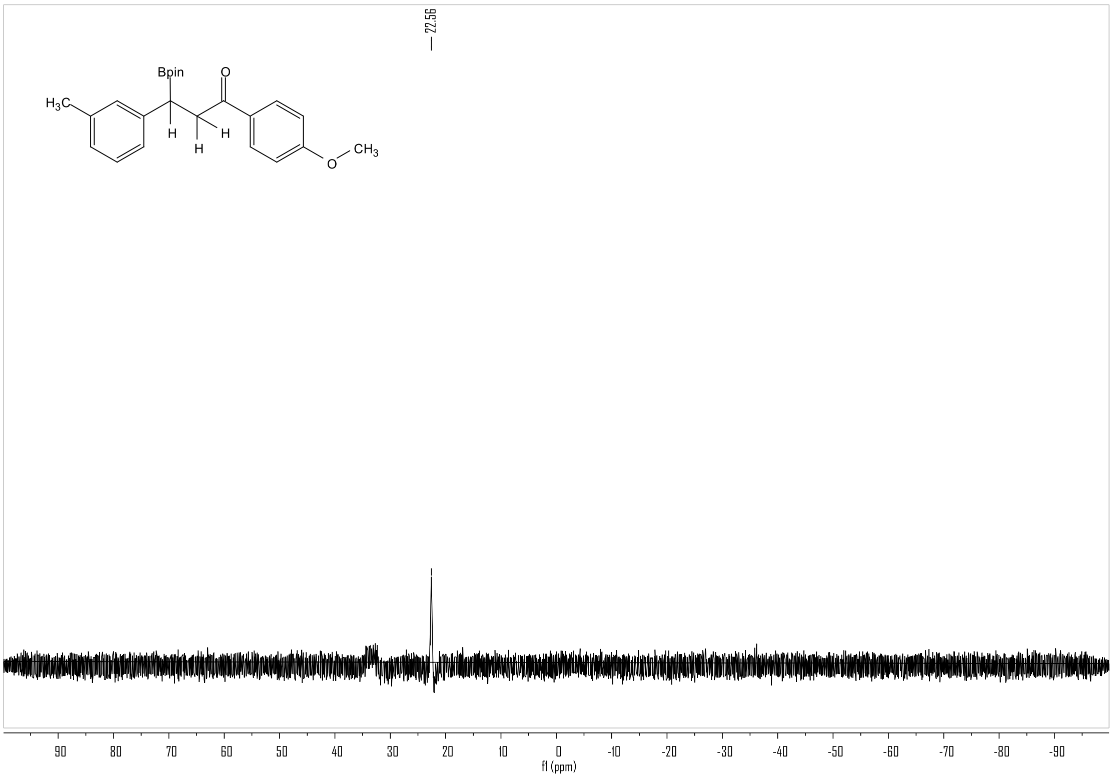

^{1}H NMR (600 MHz) Spectrum of **3ja** in $CDCl_3$

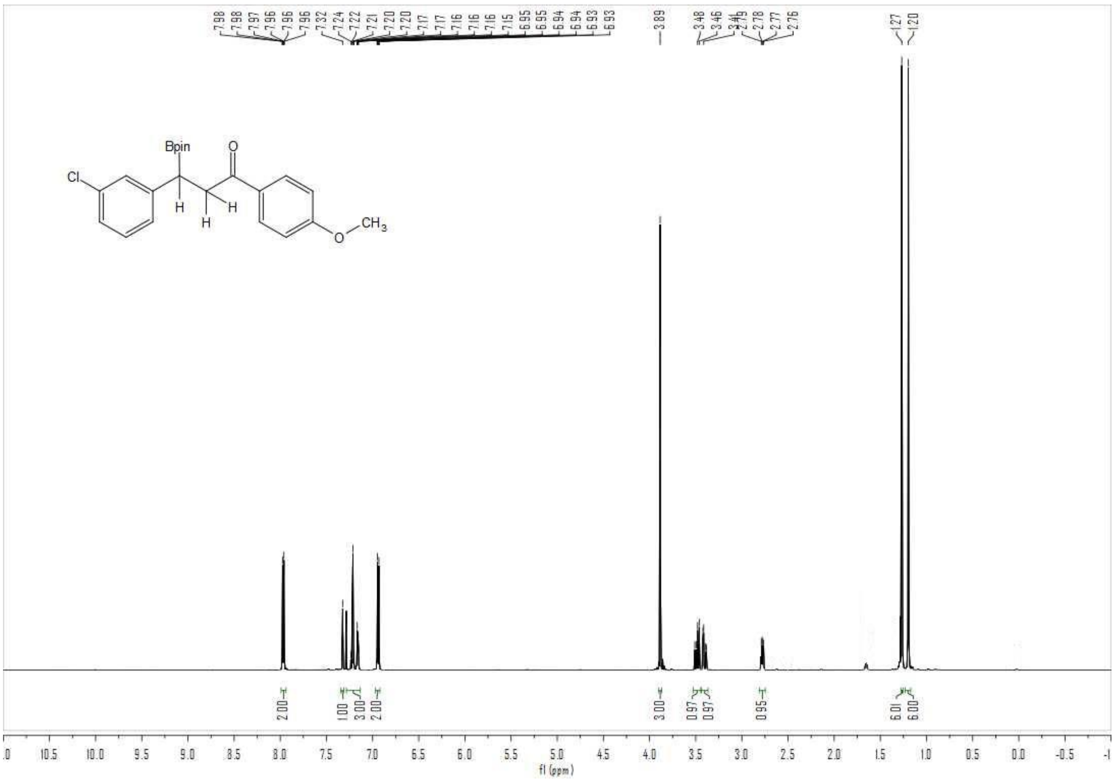

$^{13}C\{^{1}H\}$ NMR (151 MHz) Spectrum of **3ja** in $CDCl_3$

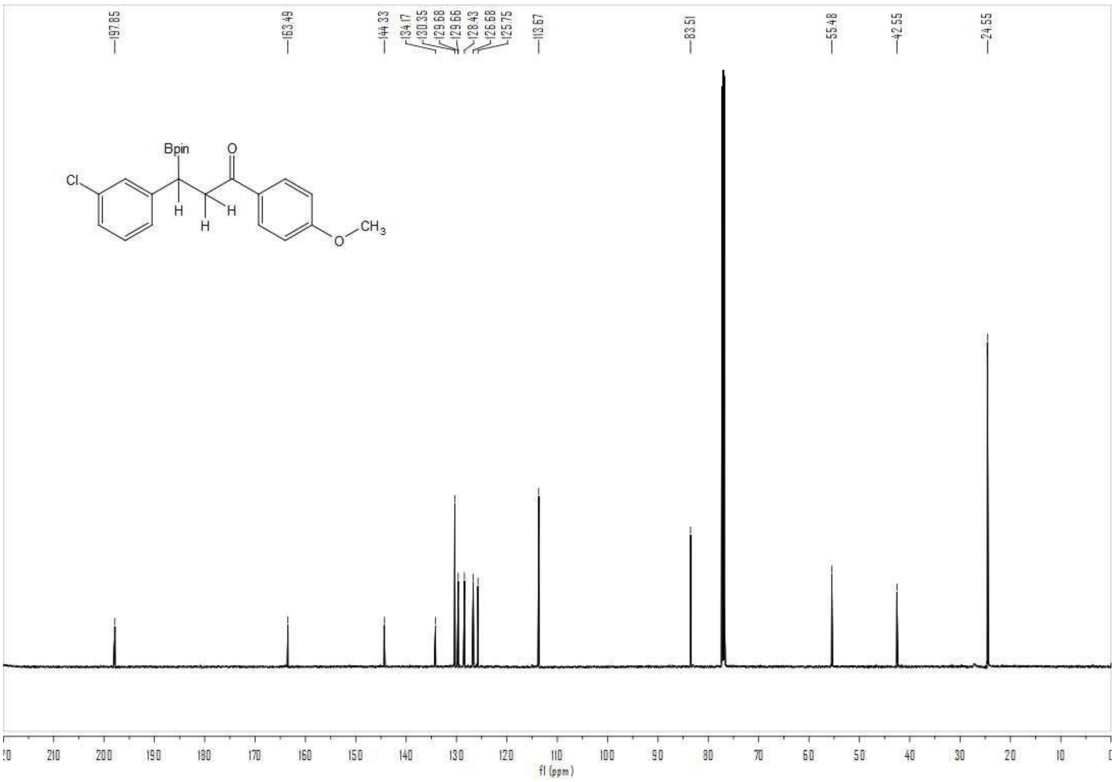

^{11}B NMR (160 MHz) Spectrum of **3ja** in $CDCl_3$

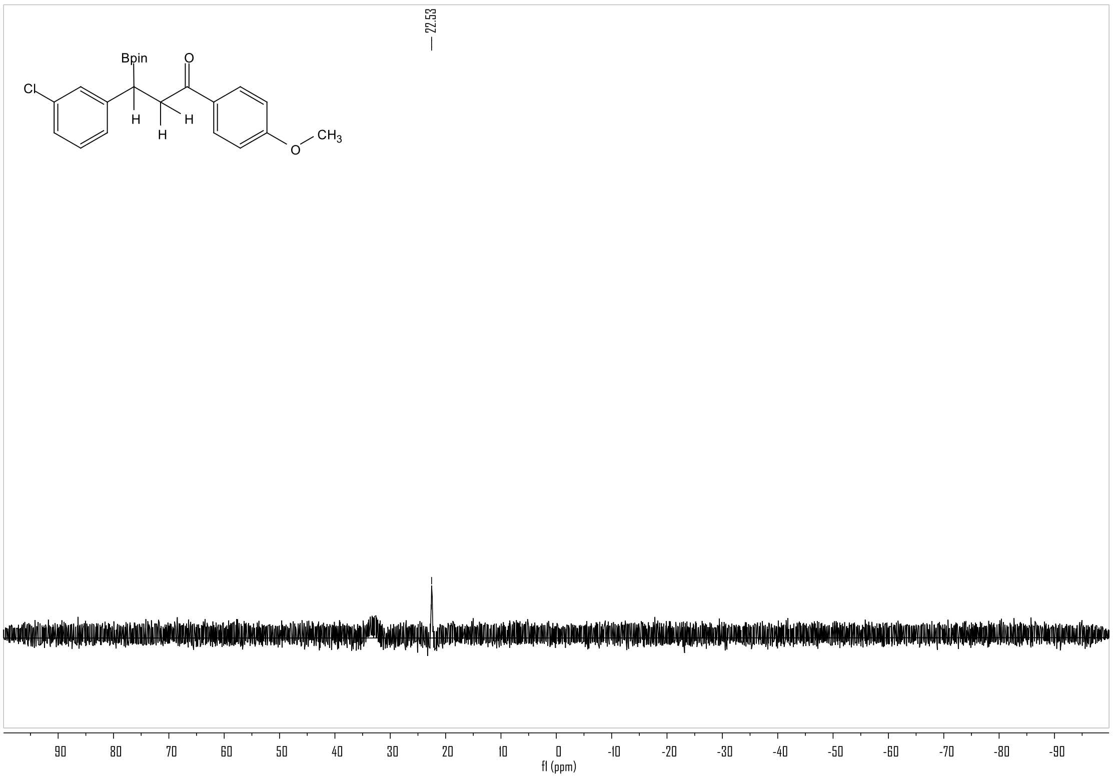

^{1}H NMR (600 MHz) Spectrum of **3ka** in $CDCl_3$

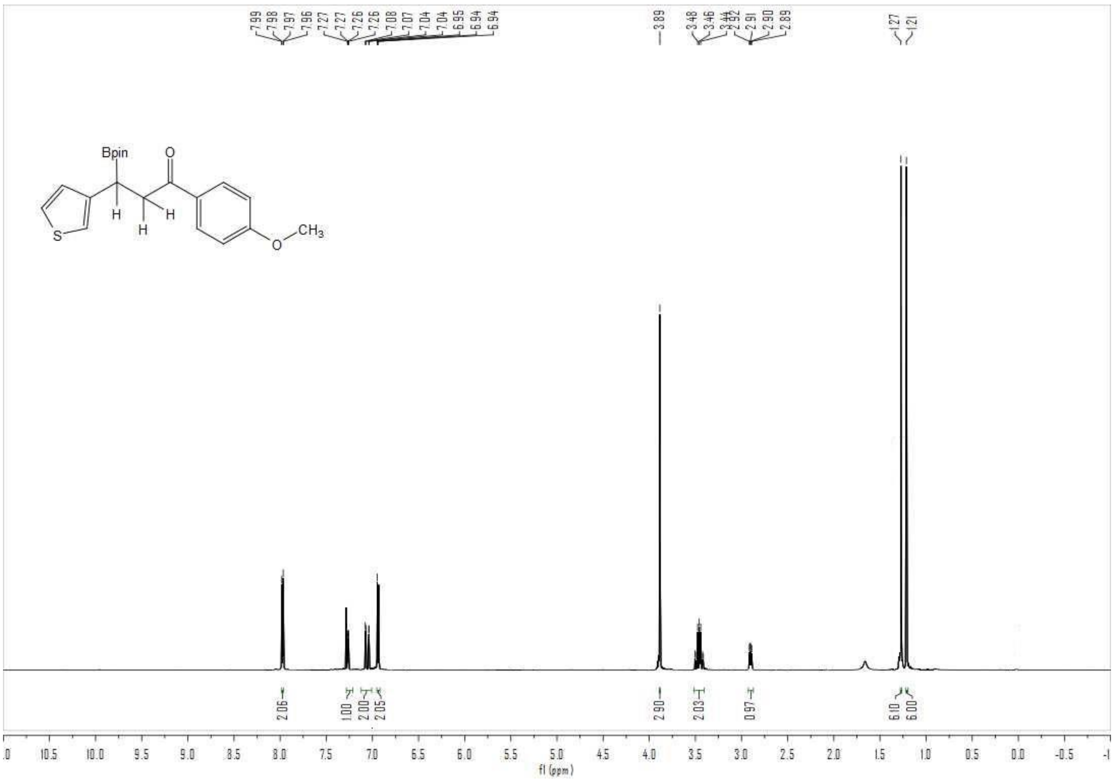

^{13}C{^{1}H} NMR (151 MHz) Spectrum of **3ka** in $CDCl_3$

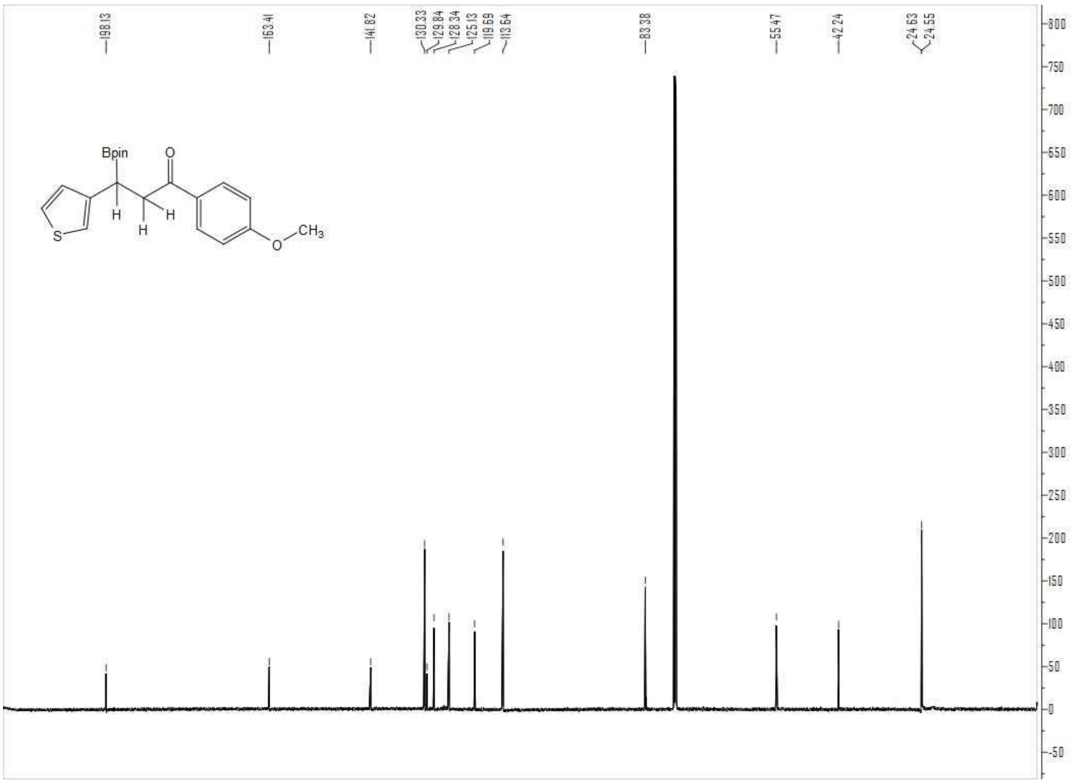

^{11}B NMR (160 MHz) Spectrum of **3ka** in $CDCl_3$

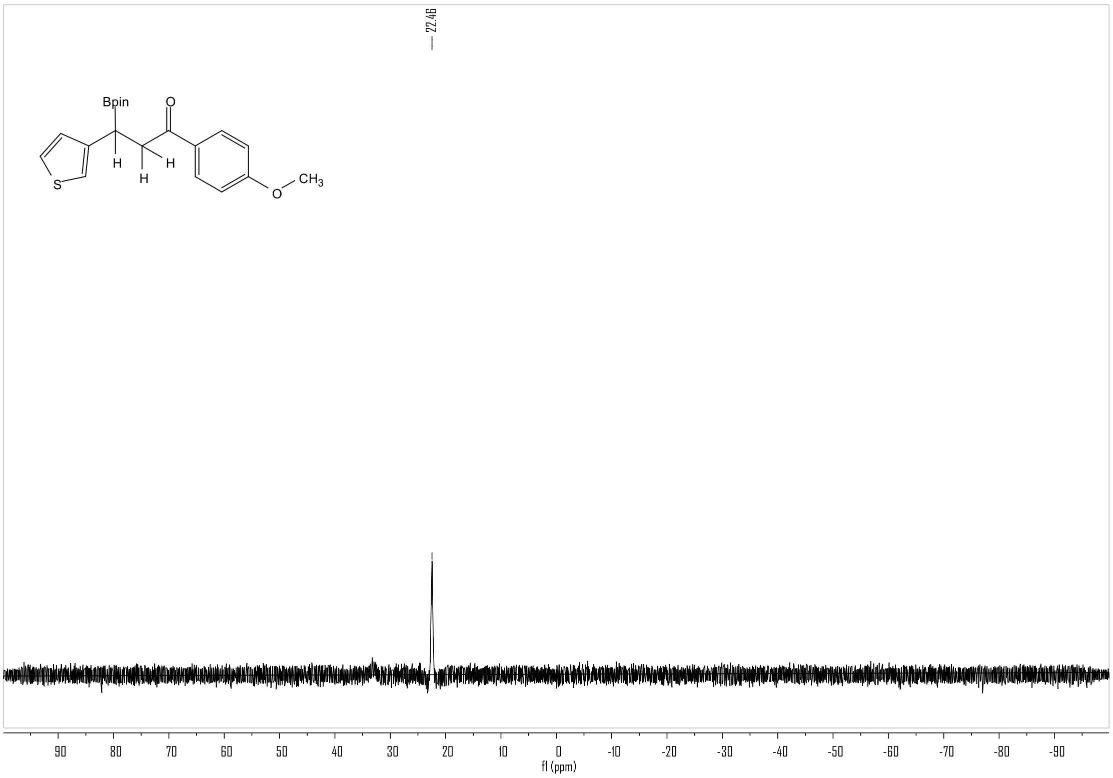

^{1}H NMR (600 MHz) Spectrum of **3la** in $CDCl_3$

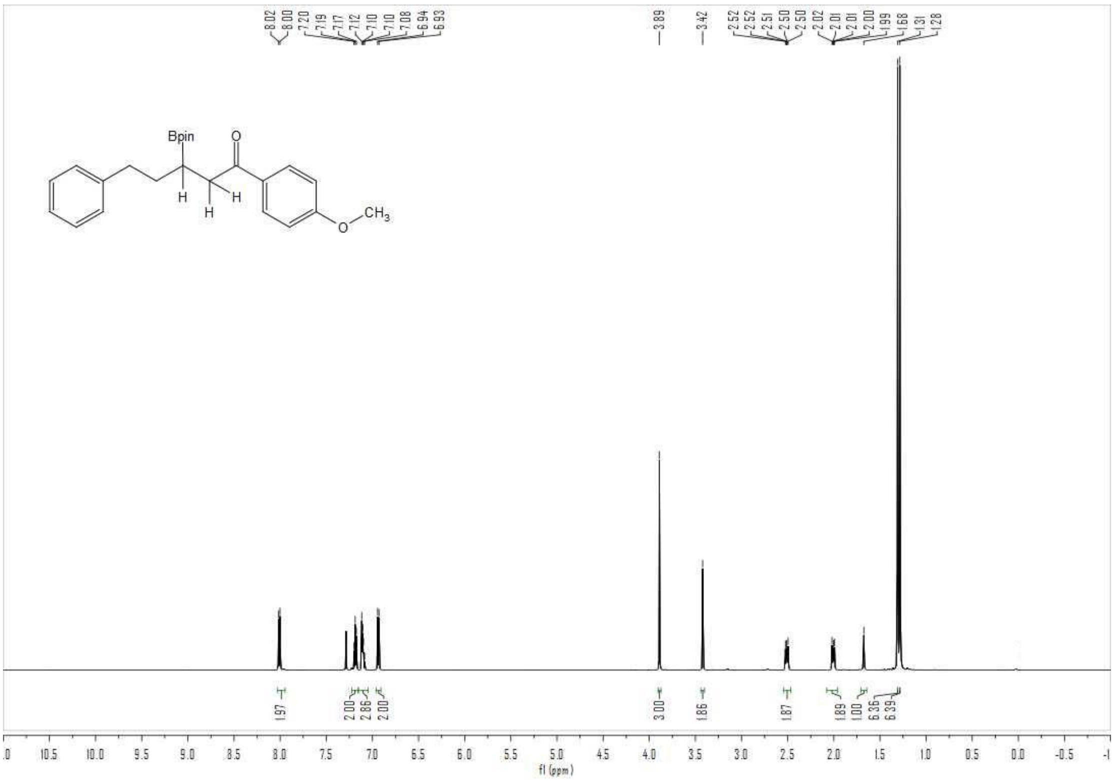

$^{13}C\{^{1}H\}$ NMR (151 MHz) Spectrum of **3la** in $CDCl_3$

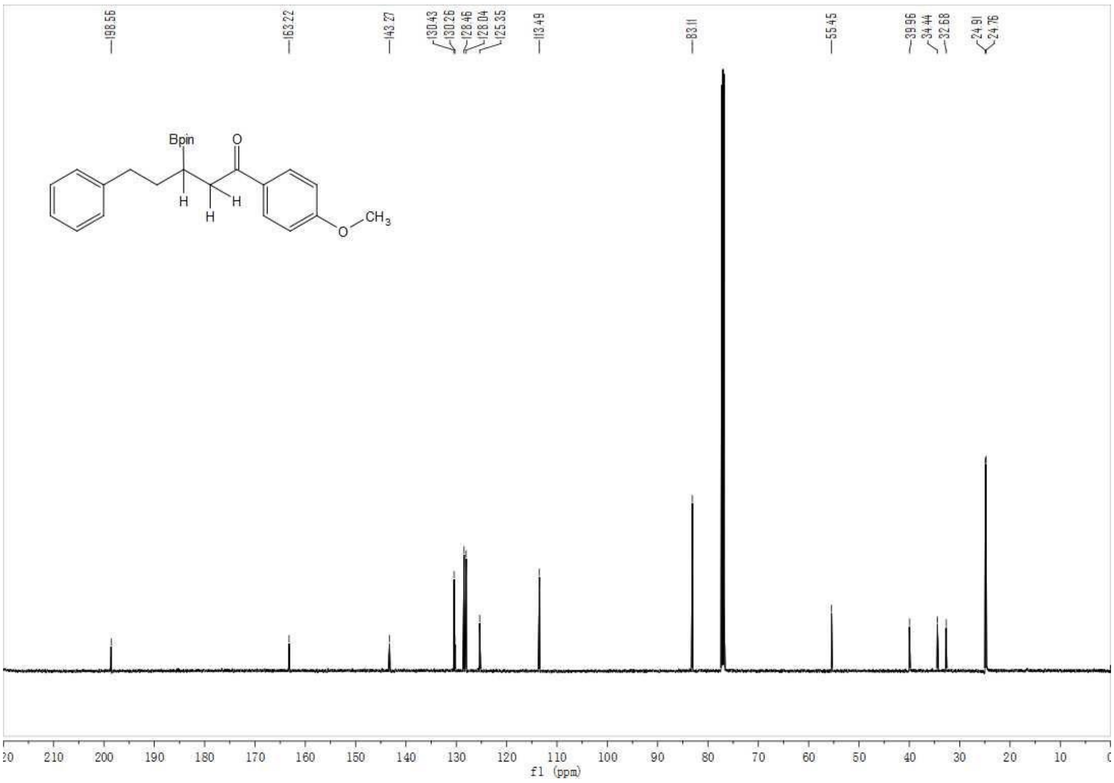

^{11}B NMR (160 MHz) Spectrum of **3la** in $CDCl_3$

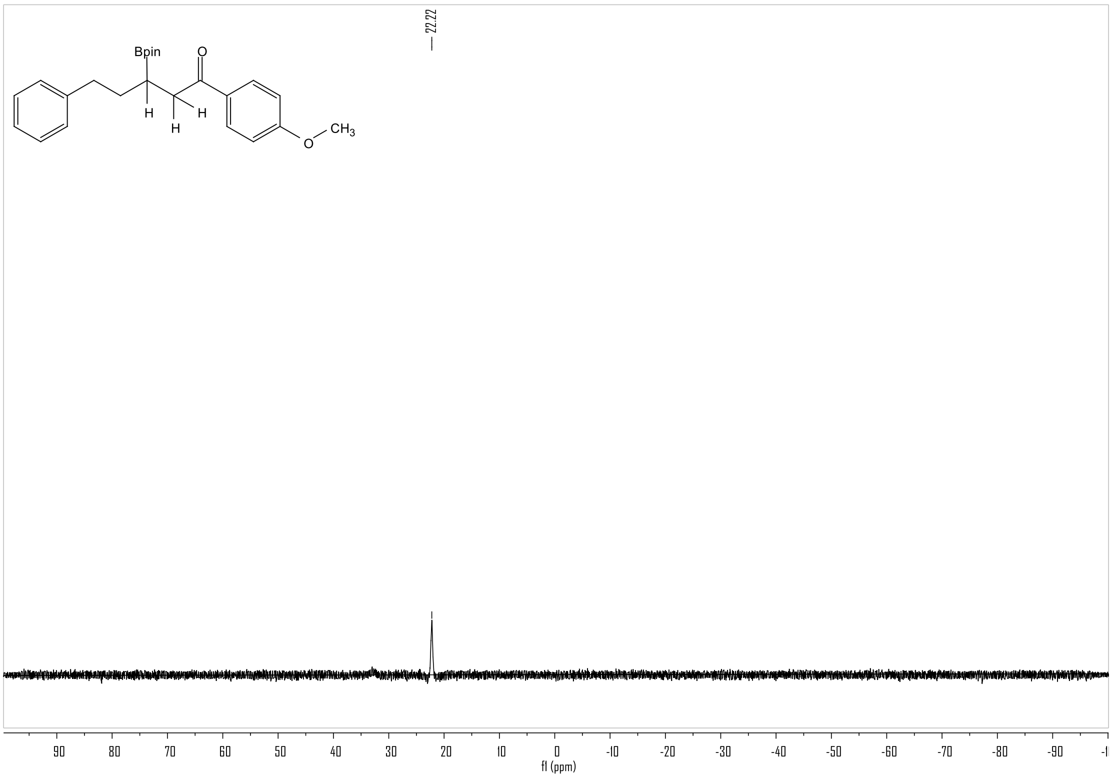

^{1}H NMR (600 MHz) Spectrum of **3ma** in $CDCl_3$

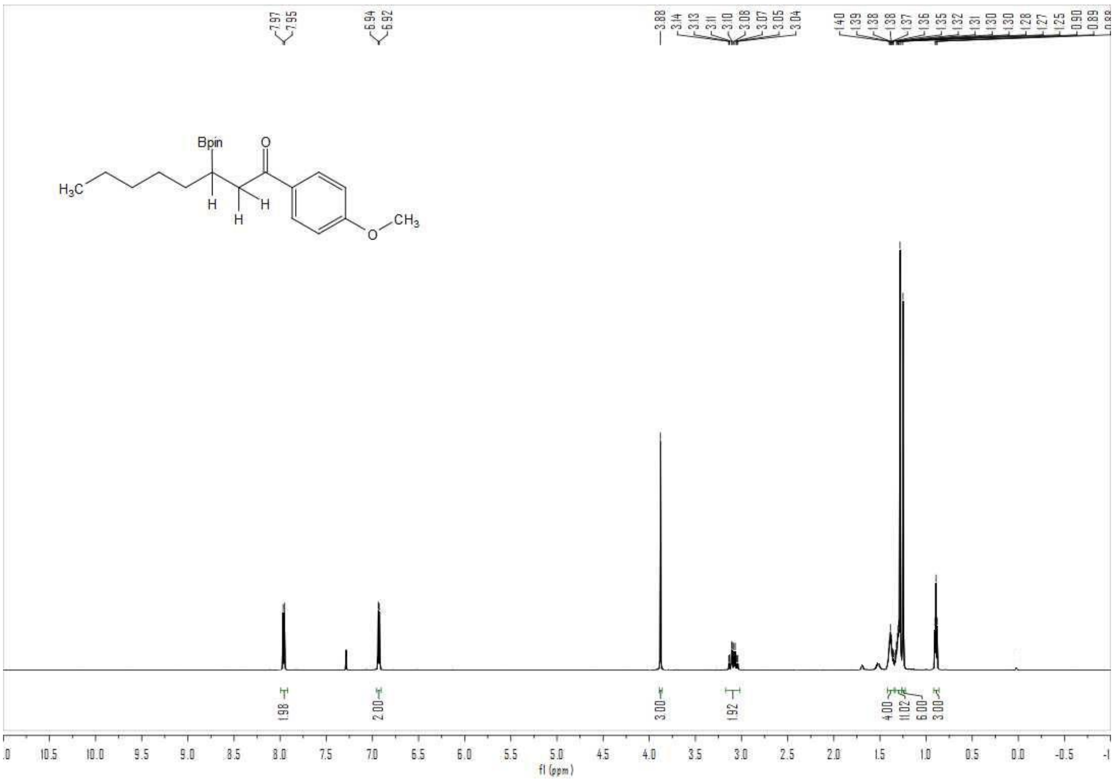

$^{13}C\{^{1}H\}$ NMR (151 MHz) Spectrum of **3ma** in $CDCl_3$

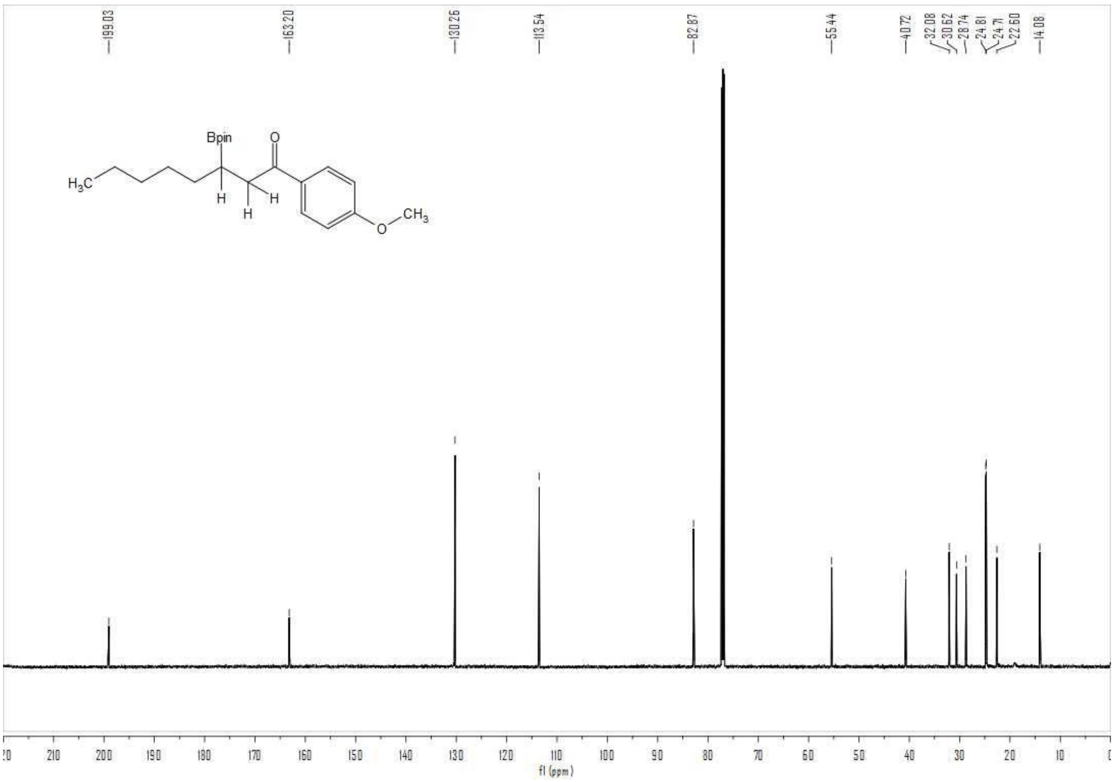

^{11}B NMR (160 MHz) Spectrum of **3ma** in $CDCl_3$

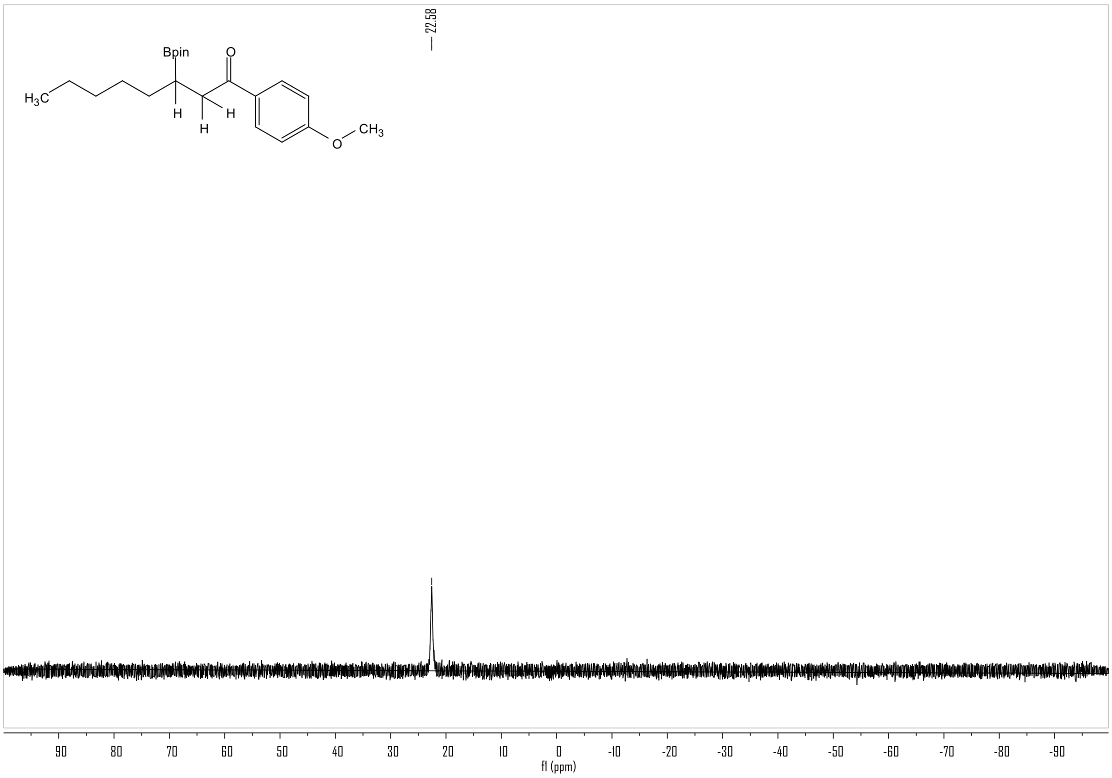

^{1}H NMR (600 MHz) Spectrum of **3na** in $CDCl_3$

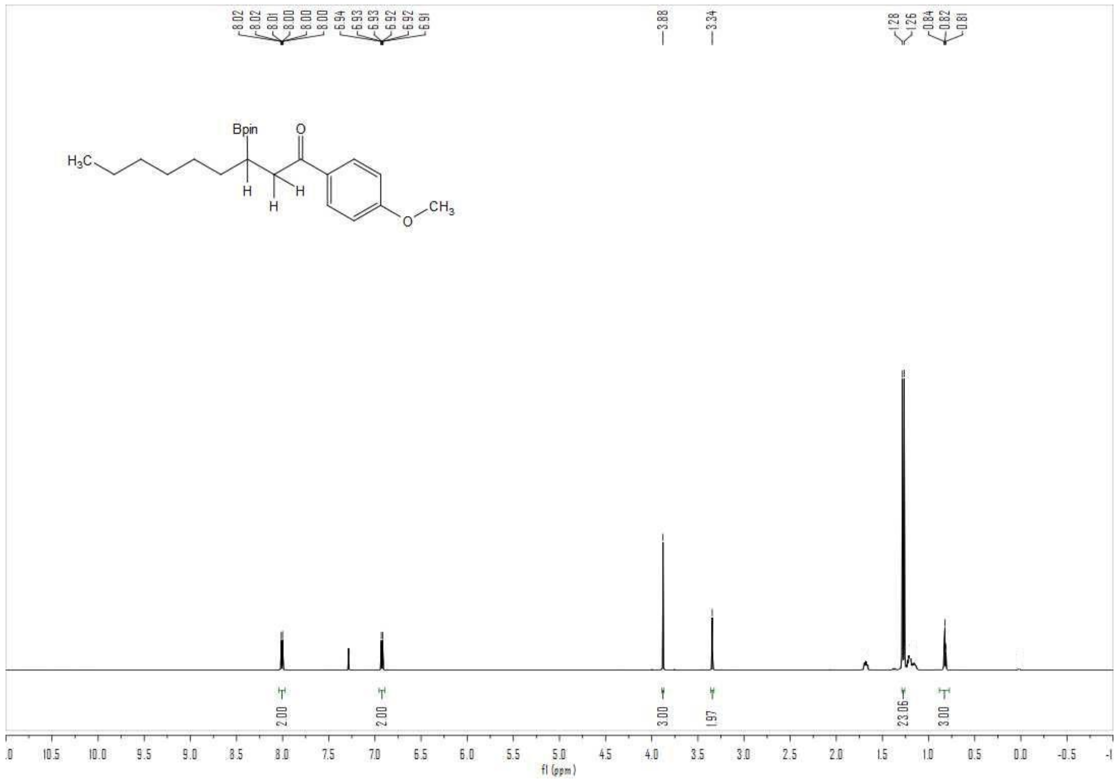

$^{13}C\{^{1}H\}$ NMR (151 MHz) Spectrum of **3na** in $CDCl_3$

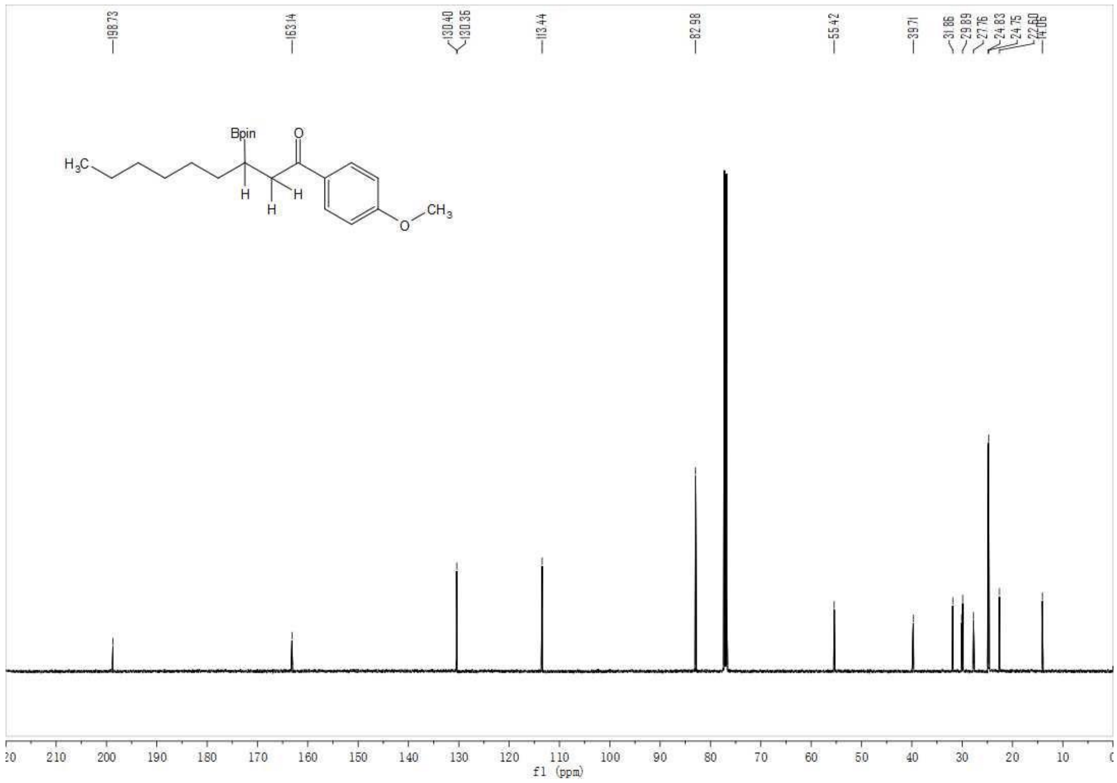

^{11}B NMR (160 MHz) Spectrum of **3na** in $CDCl_3$

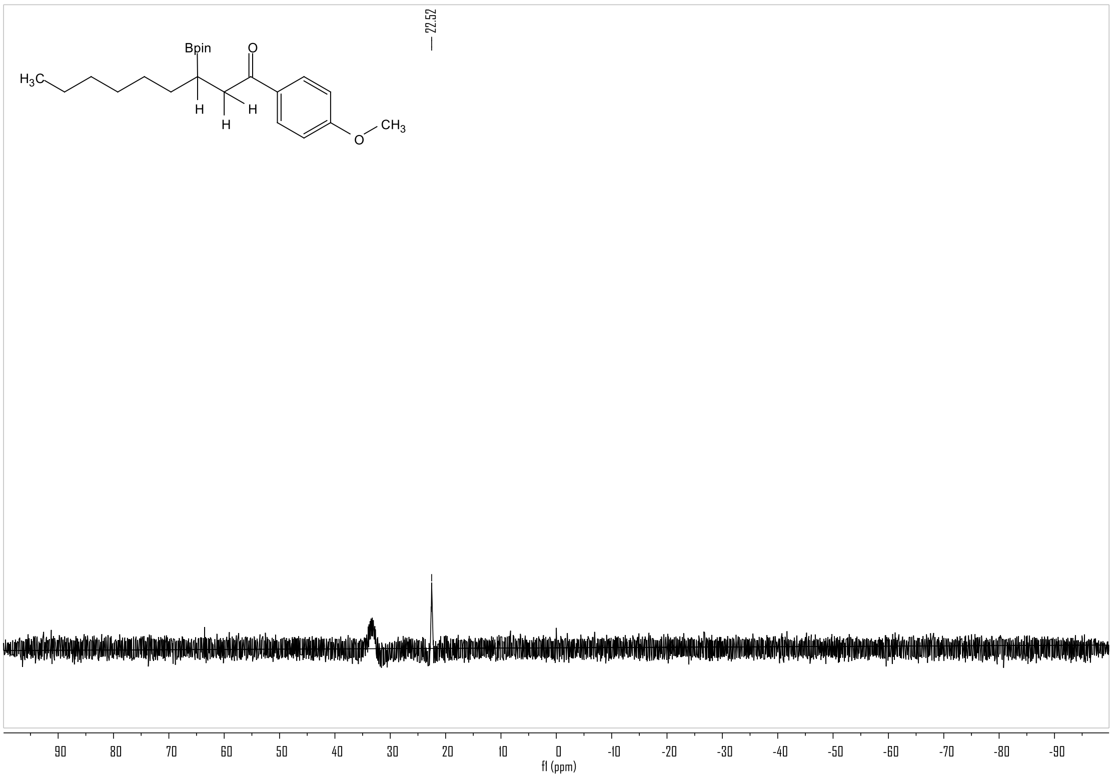

^{1}H NMR (600 MHz) Spectrum of **3ab** in $CDCl_3$

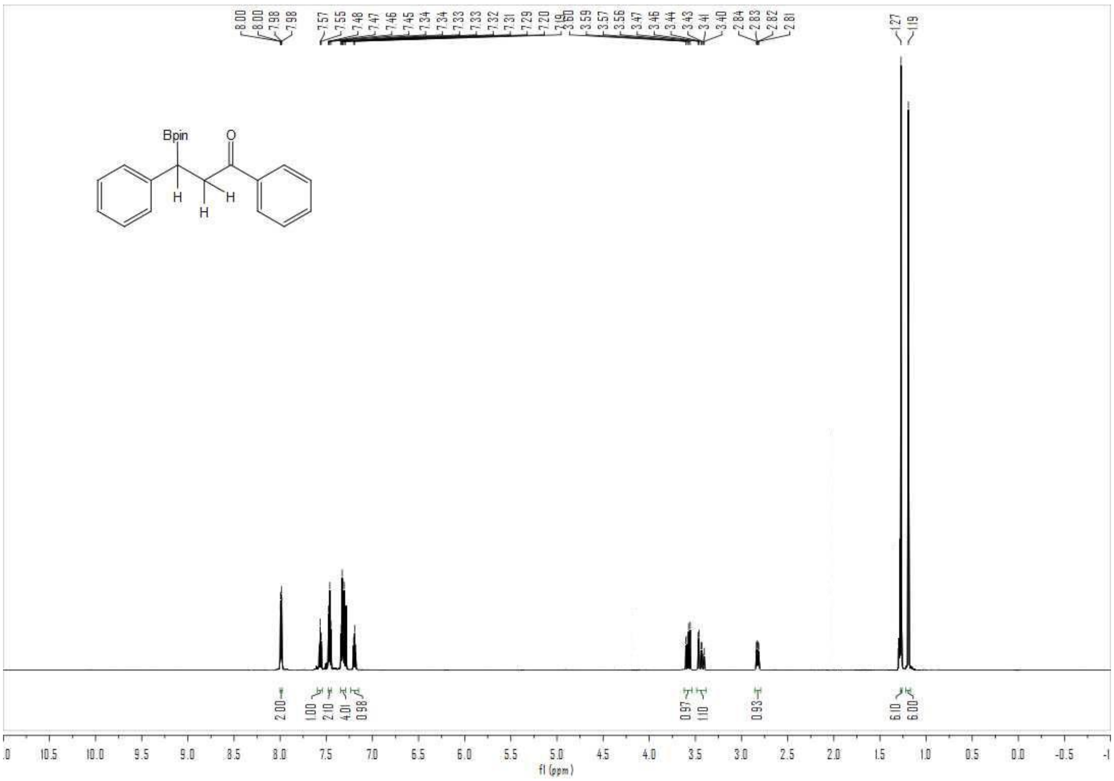

$^{13}C\{^{1}H\}$ NMR (151 MHz) Spectrum of **3ab** in $CDCl_3$

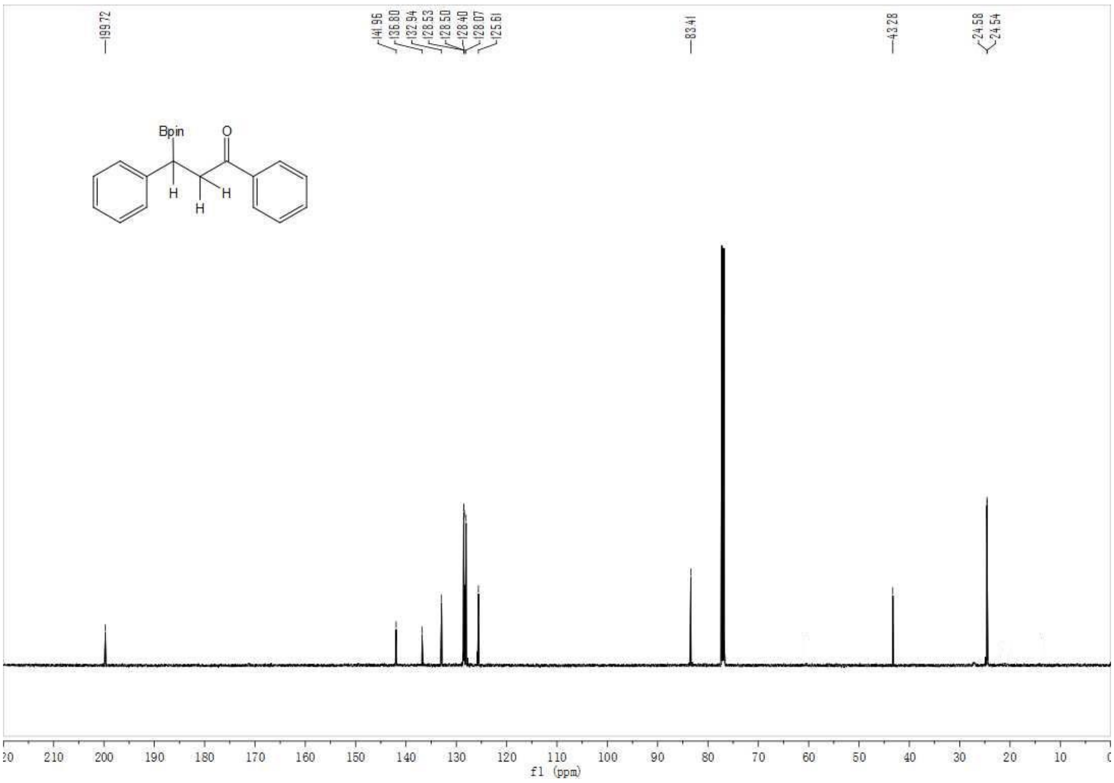

^{11}B NMR (160 MHz) Spectrum of **3ab** in $CDCl_3$

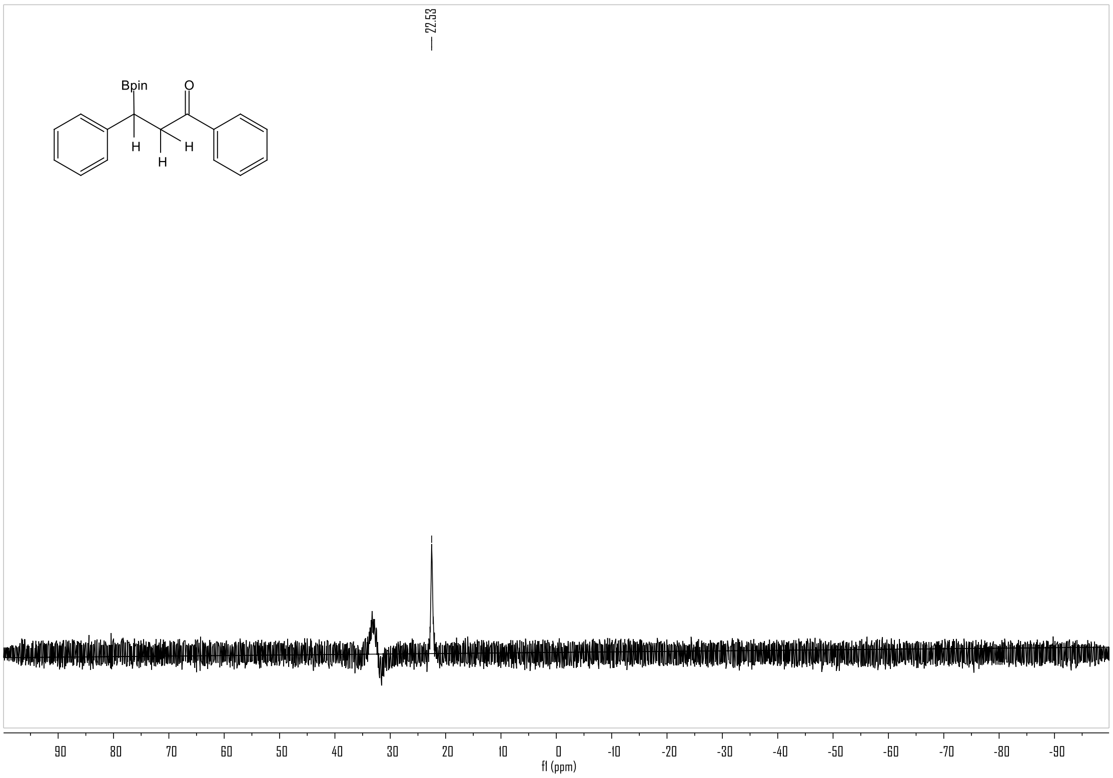

^{1}H NMR (600 MHz) Spectrum of **3ac** in CDCl$_3$

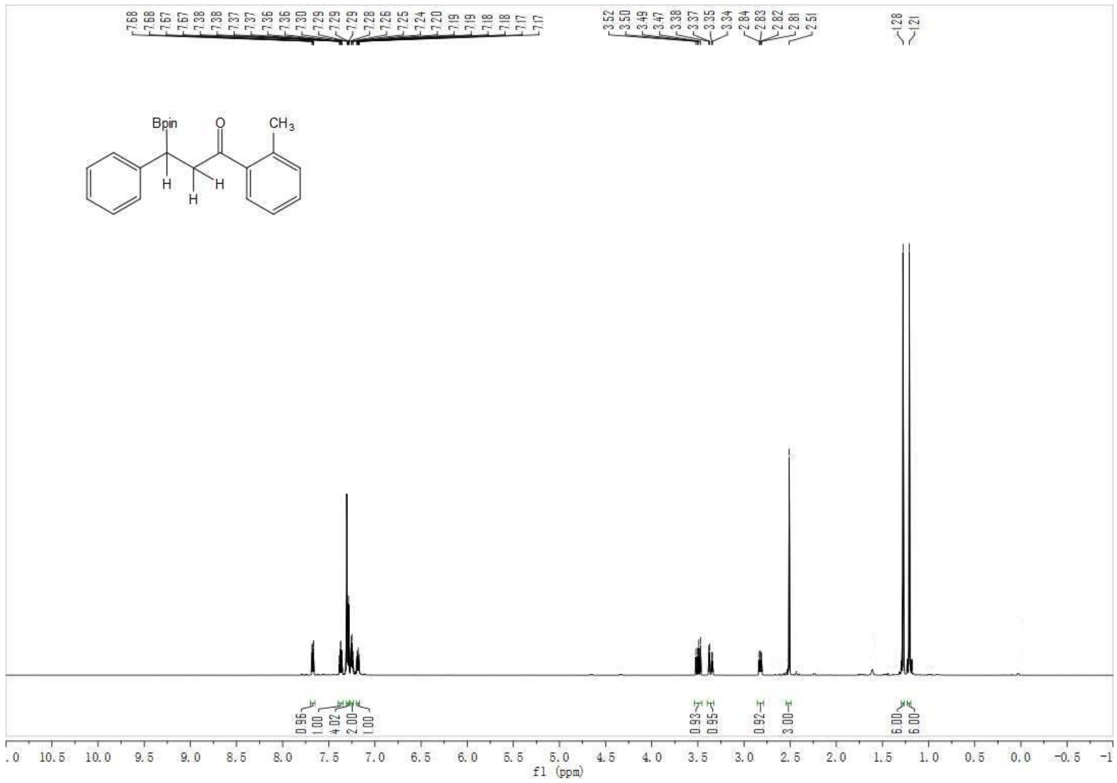

^{13}C{^{1}H} NMR (151 MHz) Spectrum of **3ac** in CDCl$_3$

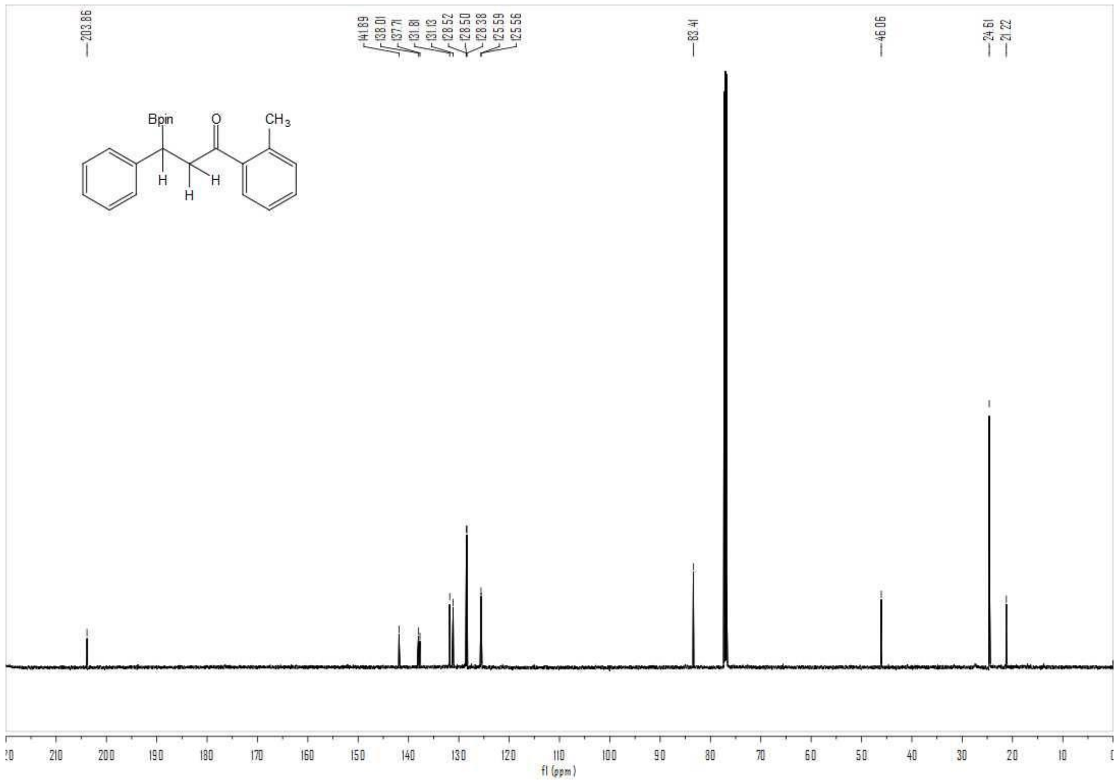

^{11}B NMR (160 MHz) Spectrum of **3ac** in $CDCl_3$

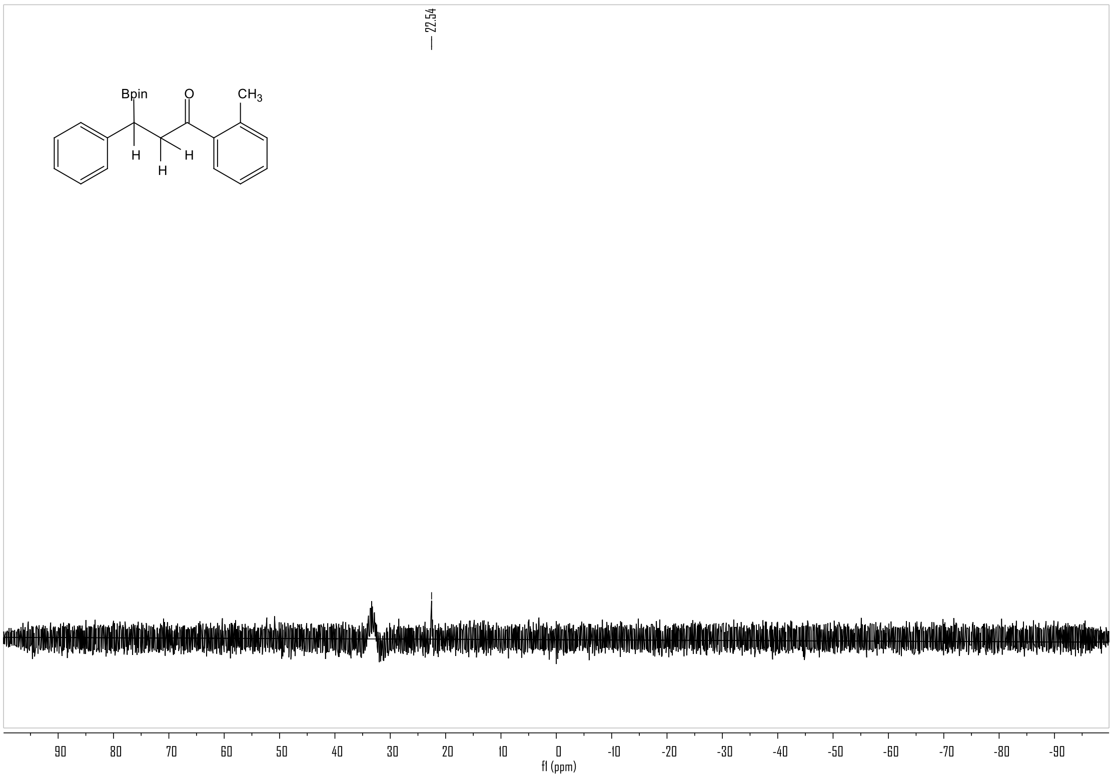

^{1}H NMR (600 MHz) Spectrum of **3ad** in $CDCl_3$

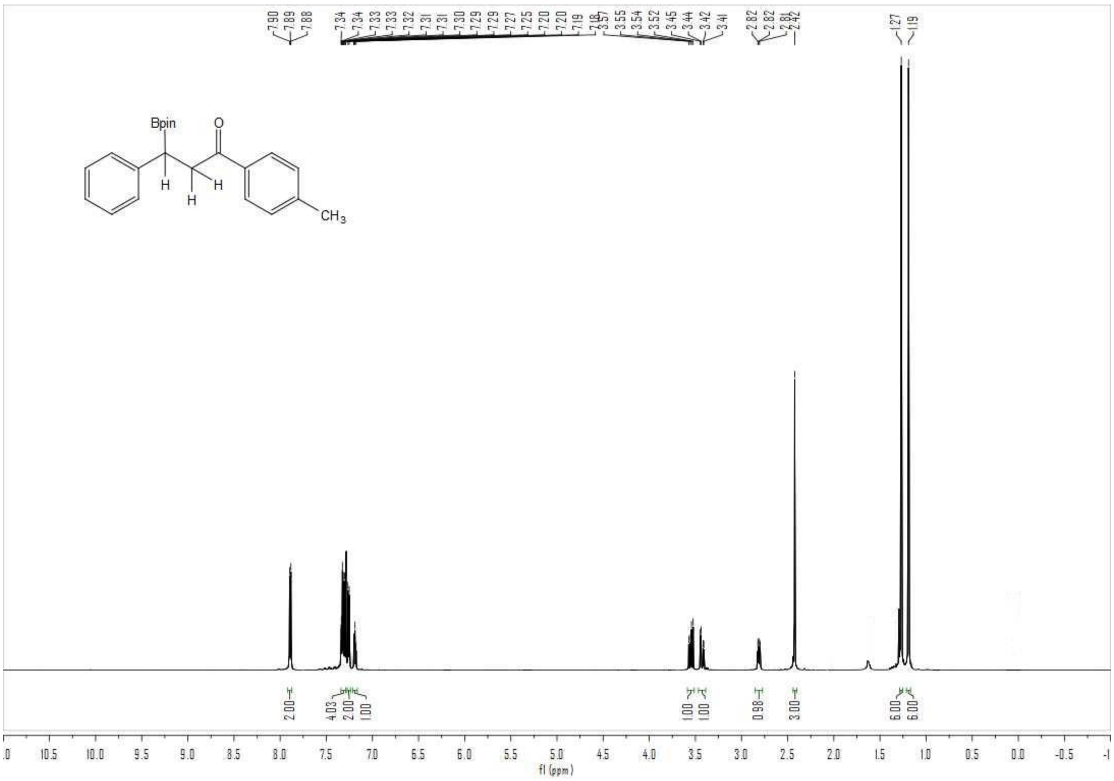

$^{13}C\{^{1}H\}$ NMR (151 MHz) Spectrum of **3ad** in $CDCl_3$

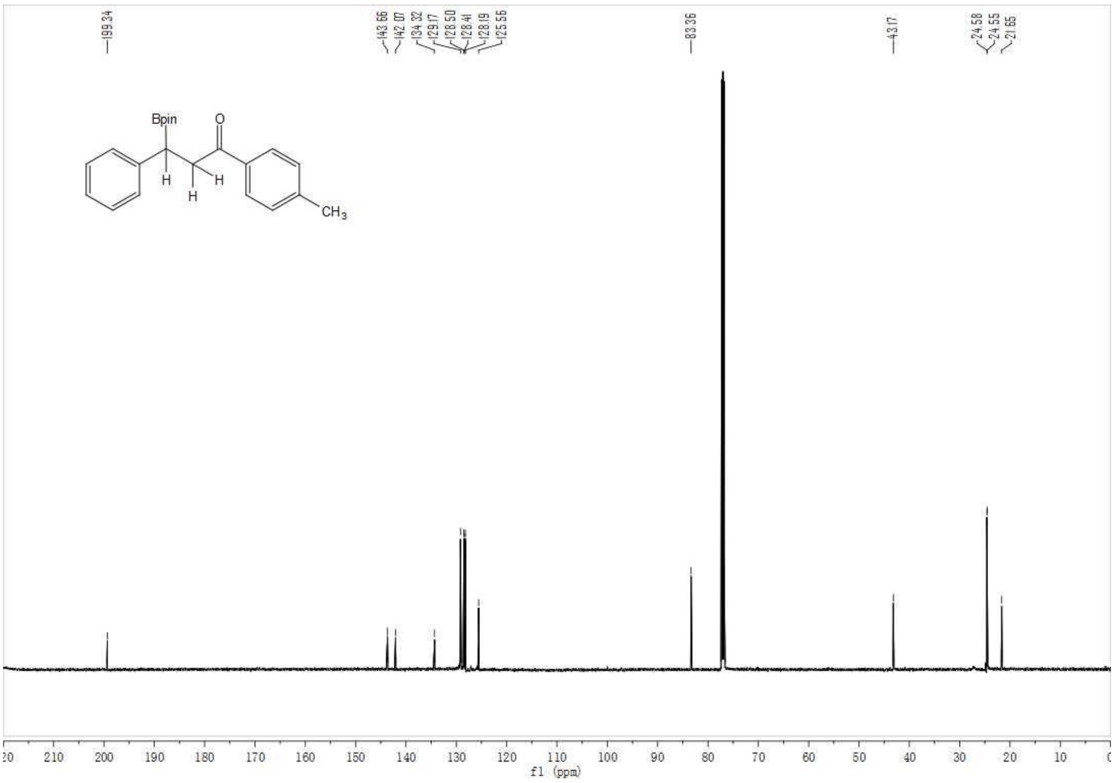

^{11}B NMR (160 MHz) Spectrum of **3ad** in $CDCl_3$

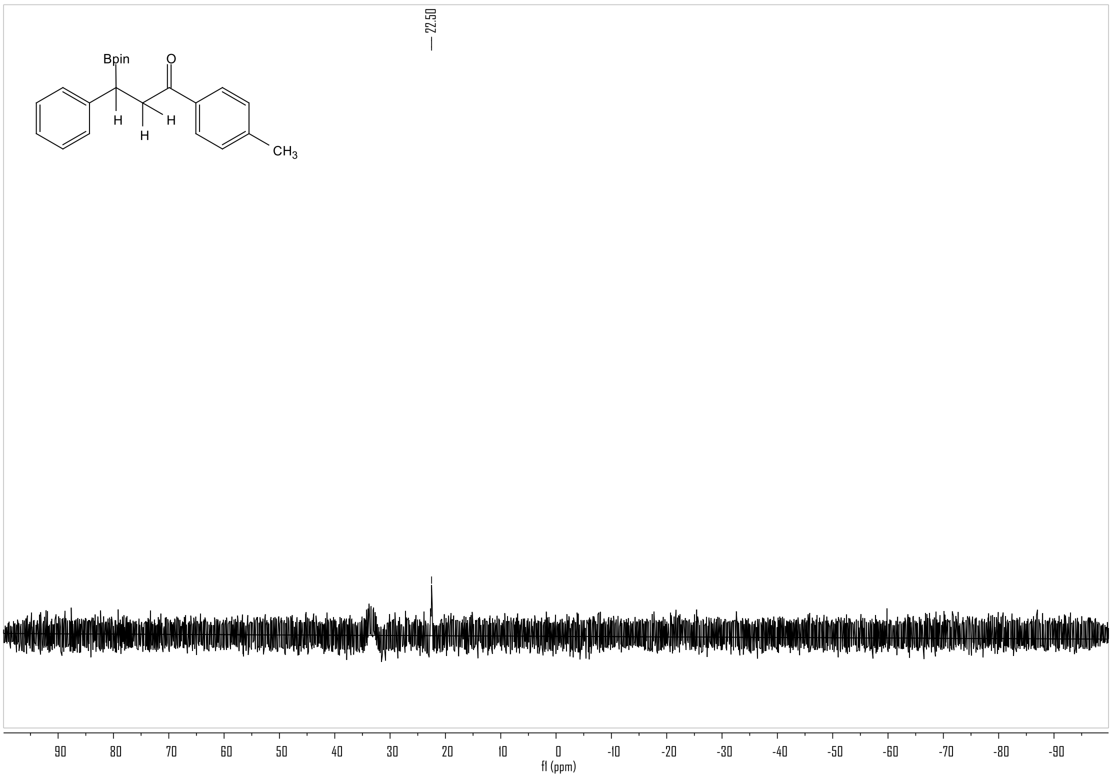

^{1}H NMR (600 MHz) Spectrum of **3ae** in $CDCl_3$

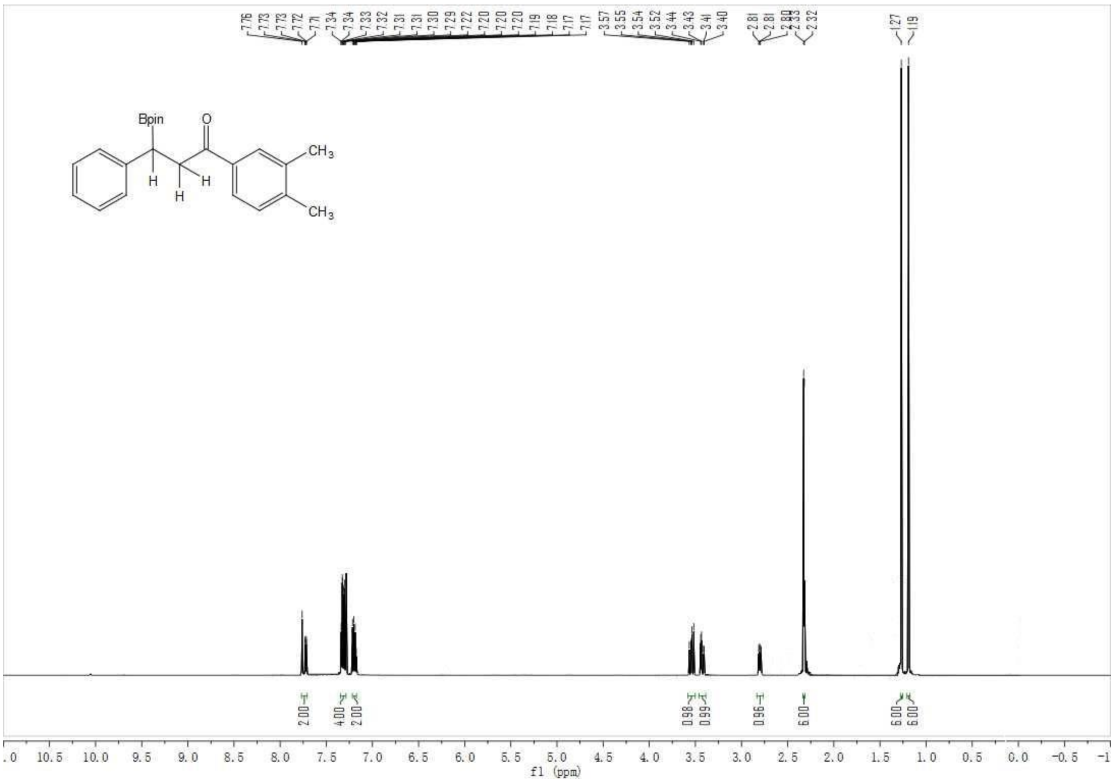

^{13}C{^{1}H} NMR (151 MHz) Spectrum of **3ae** in $CDCl_3$

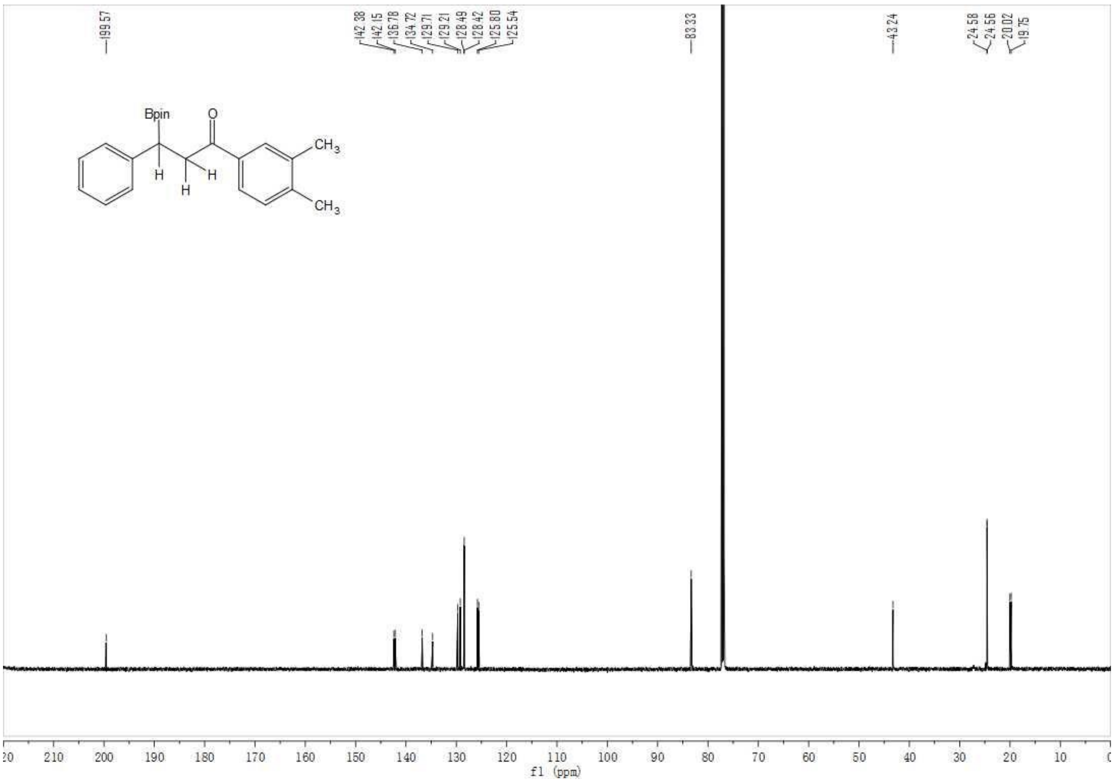

^{11}B NMR (160 MHz) Spectrum of **3ae** in $CDCl_3$

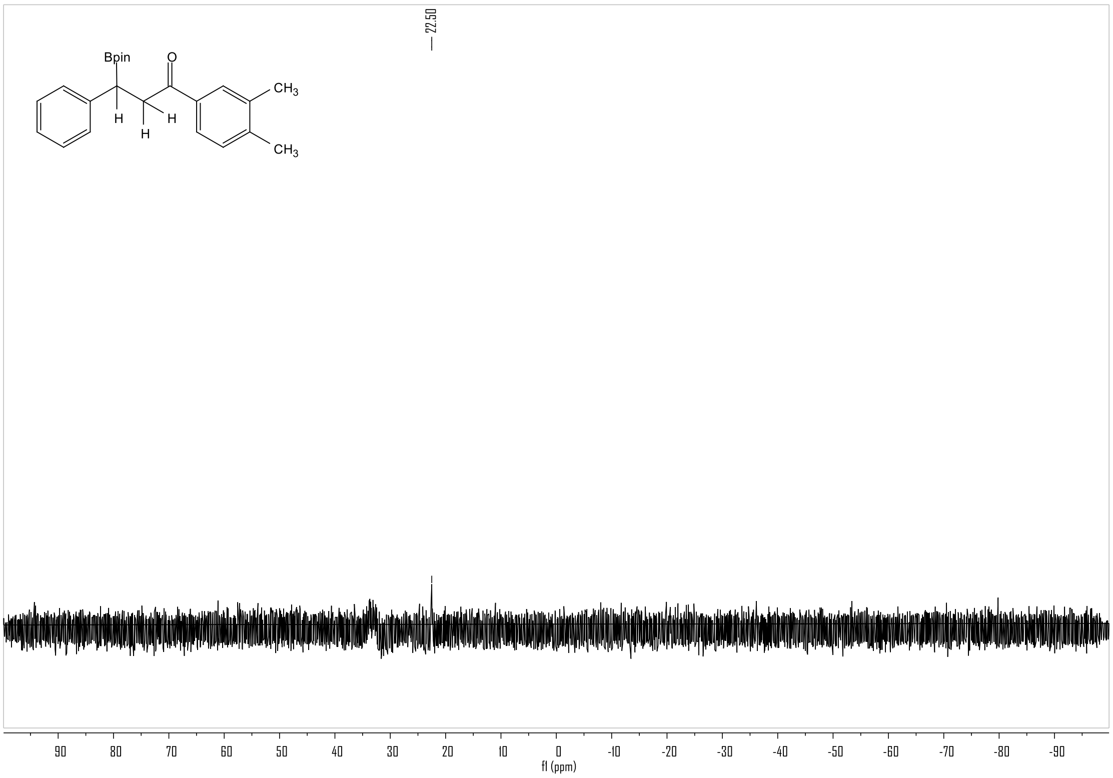

^{1}H NMR (600 MHz) Spectrum of **3af** in $CDCl_3$

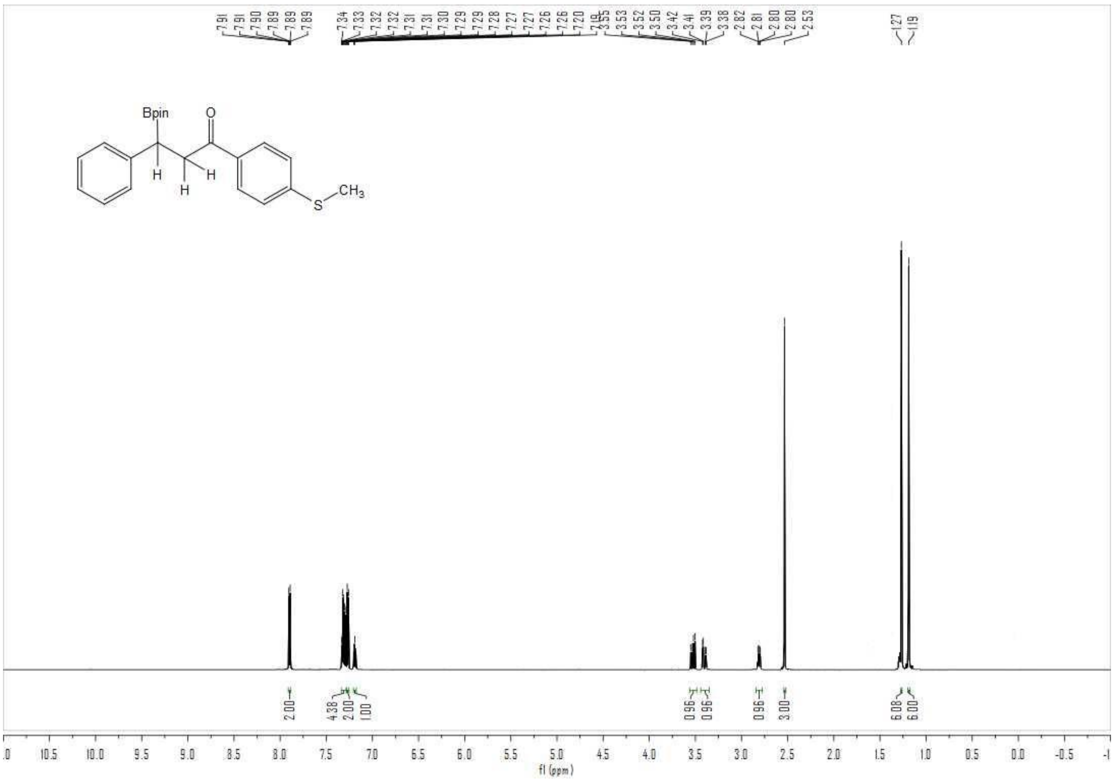

^{13}C{^{1}H} NMR (151 MHz) Spectrum of **3af** in $CDCl_3$

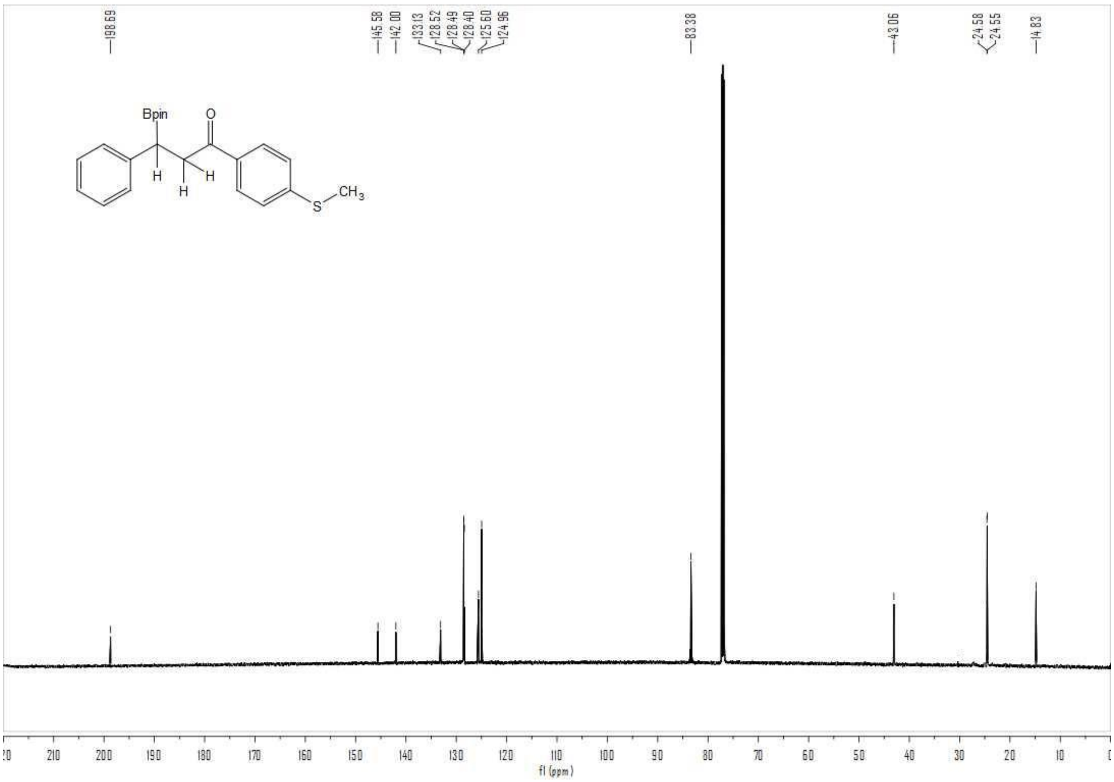

^{11}B NMR (160 MHz) Spectrum of **3af** in $CDCl_3$

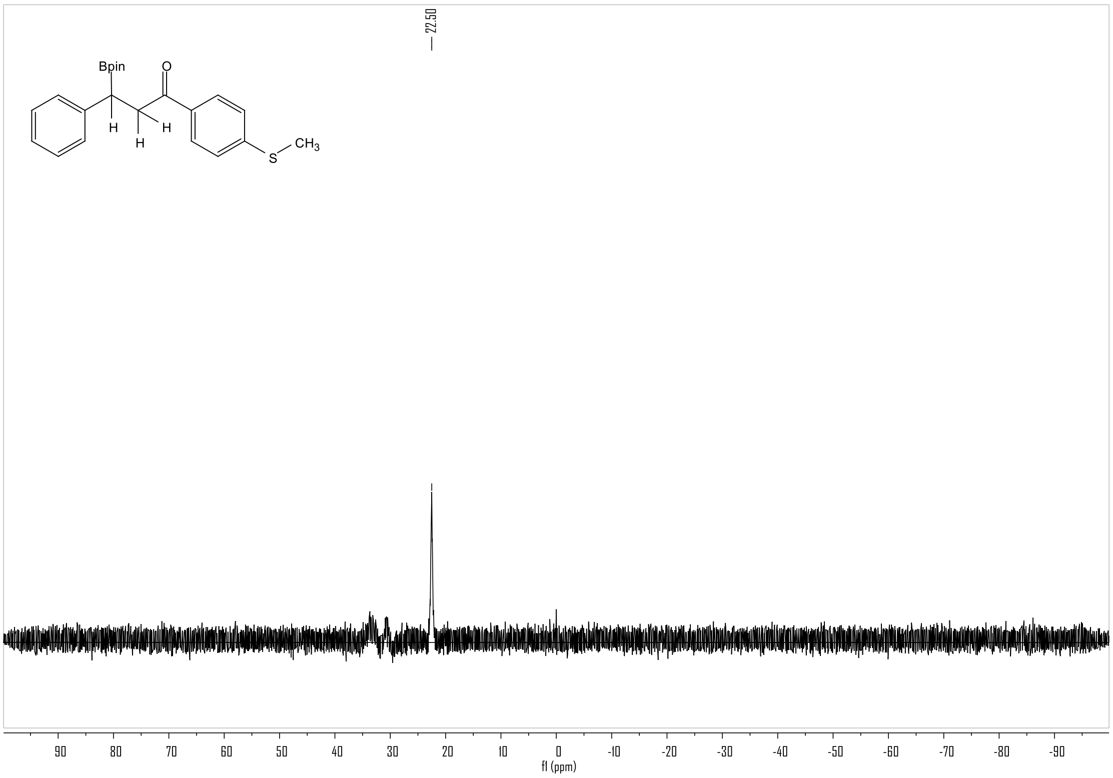

^1H NMR (600 MHz) Spectrum of **3ag** in $CDCl_3$

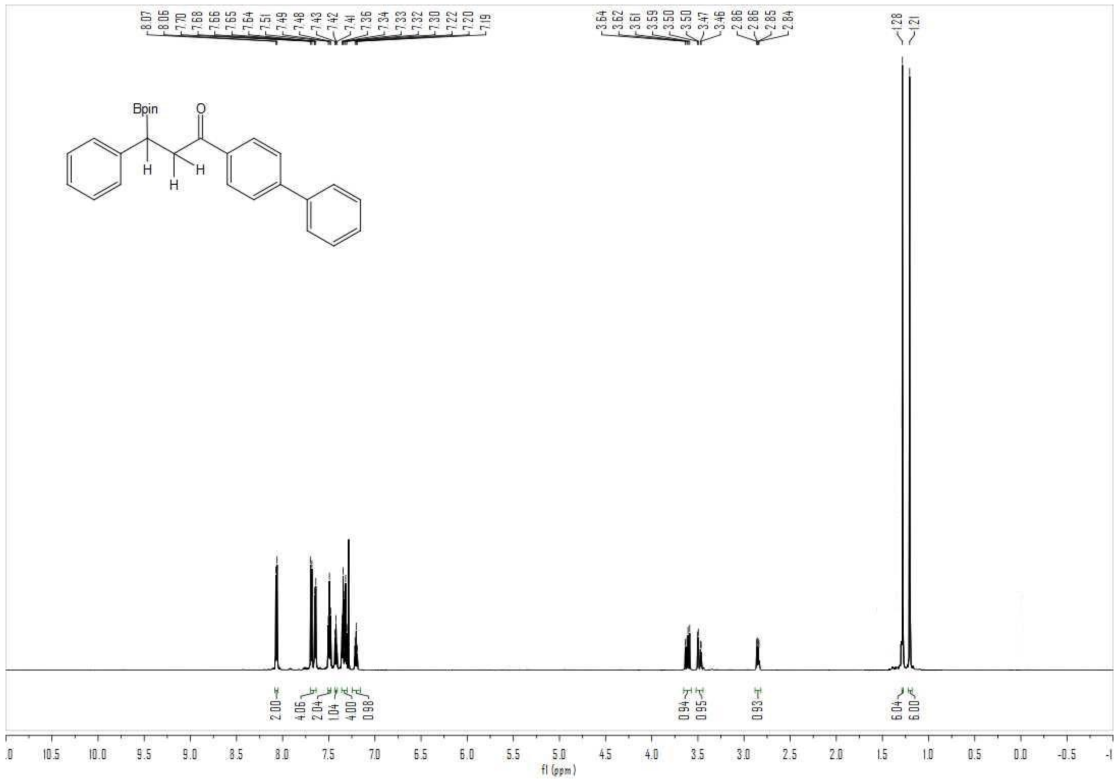

$^{13}C\{^1H\}$ NMR (151 MHz) Spectrum of **3ag** in $CDCl_3$

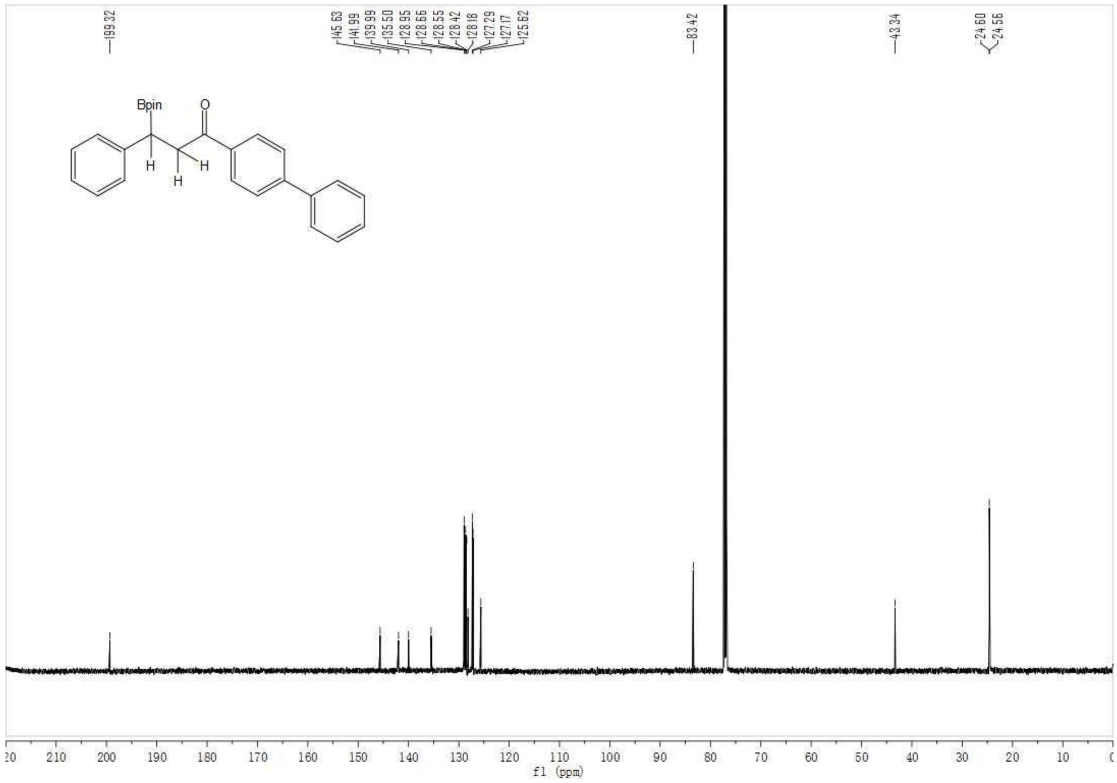

^{11}B NMR (160 MHz) Spectrum of **3ag** in $CDCl_3$

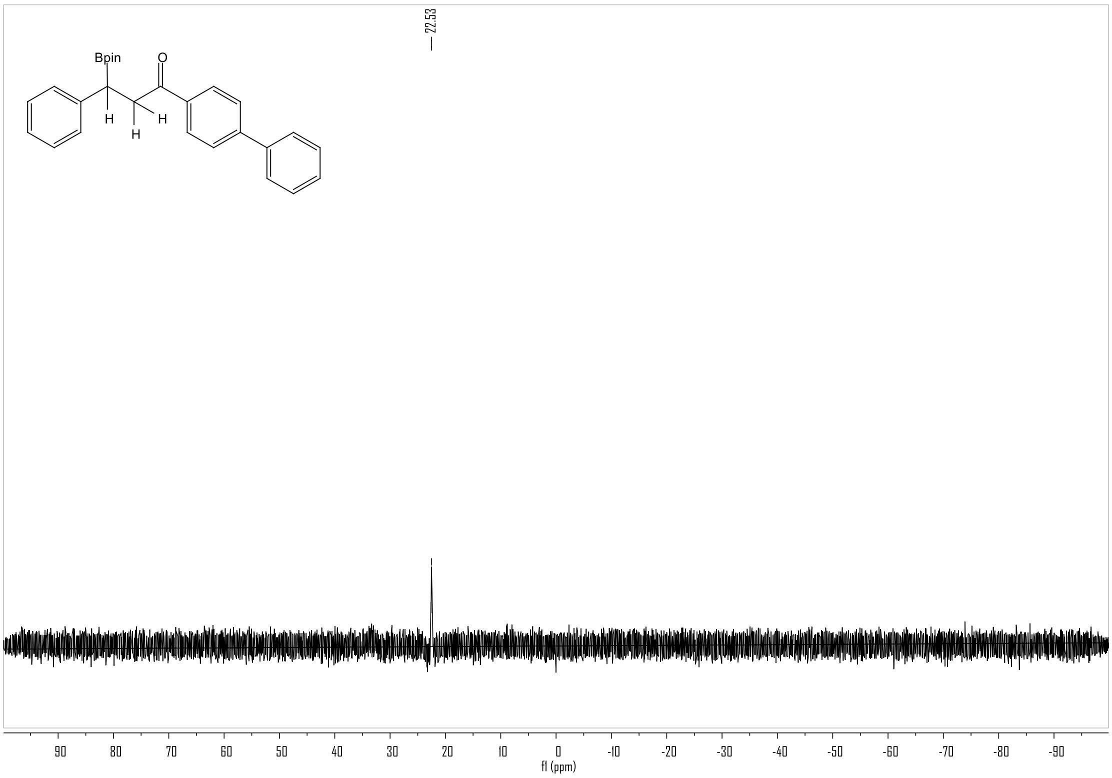

^{1}H NMR (600 MHz) Spectrum of **3ah** in $CDCl_3$

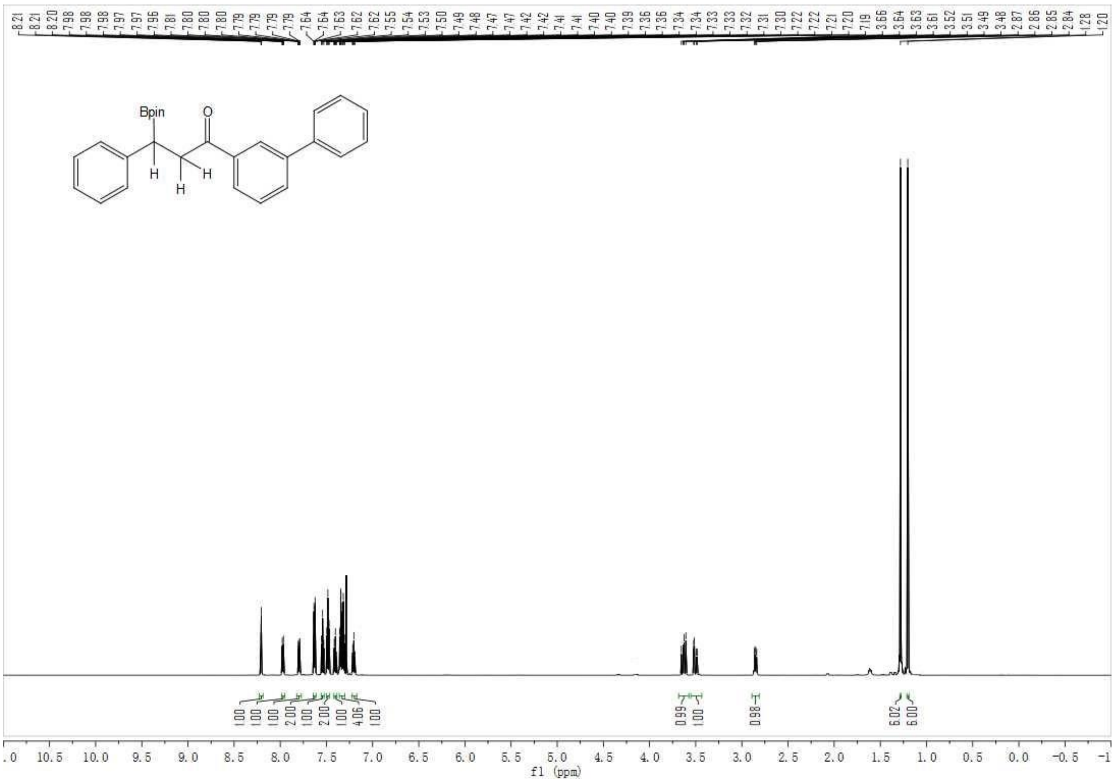

^{13}C{^{1}H} NMR (151 MHz) Spectrum of **3ah** in $CDCl_3$

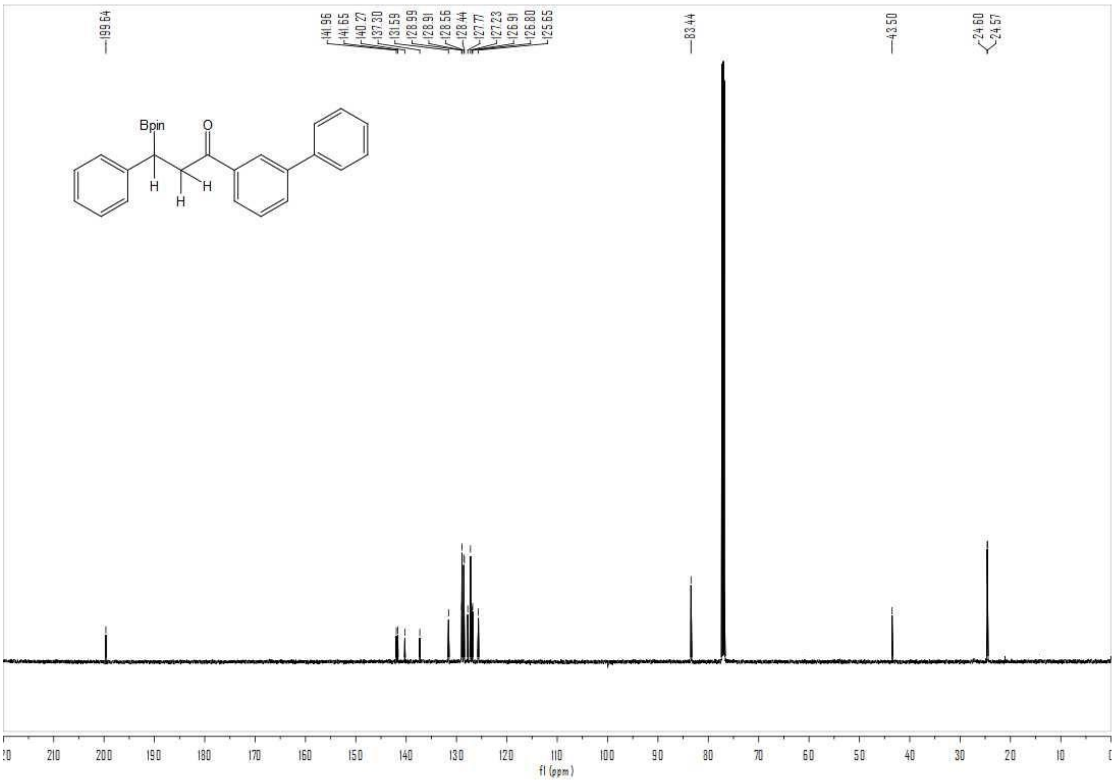

^{11}B NMR (160 MHz) Spectrum of **3ah** in $CDCl_3$

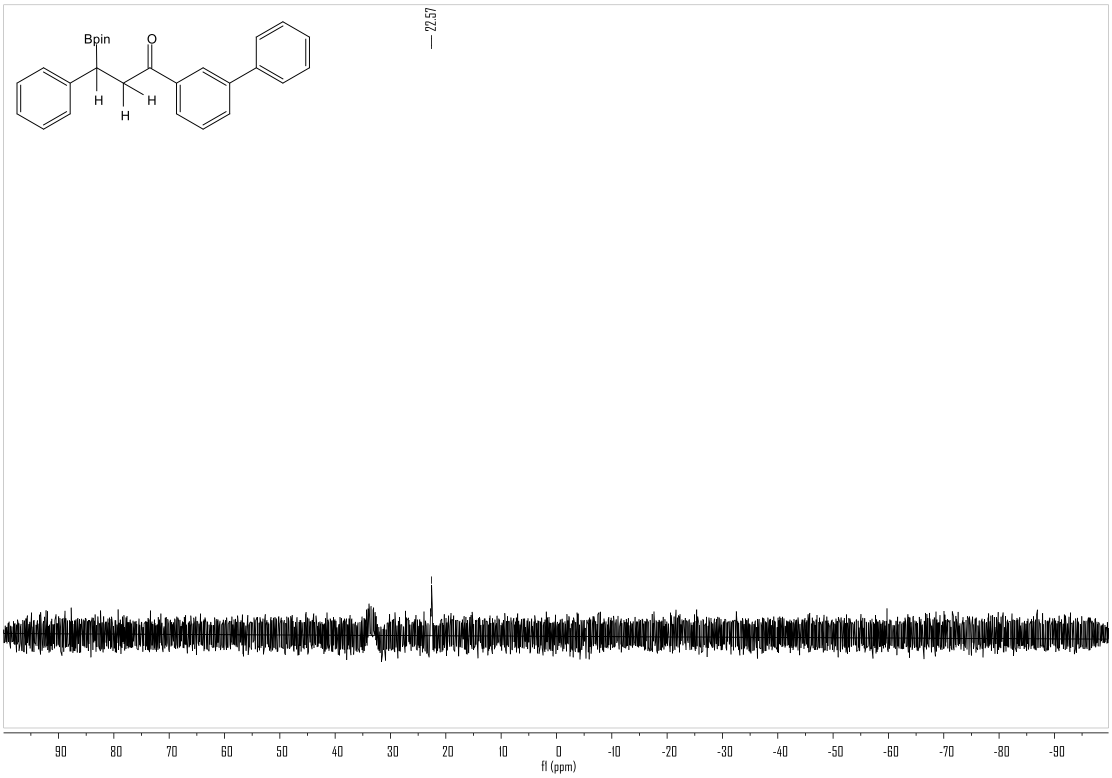

^{1}H NMR (600 MHz) Spectrum of **3ai** in $CDCl_3$

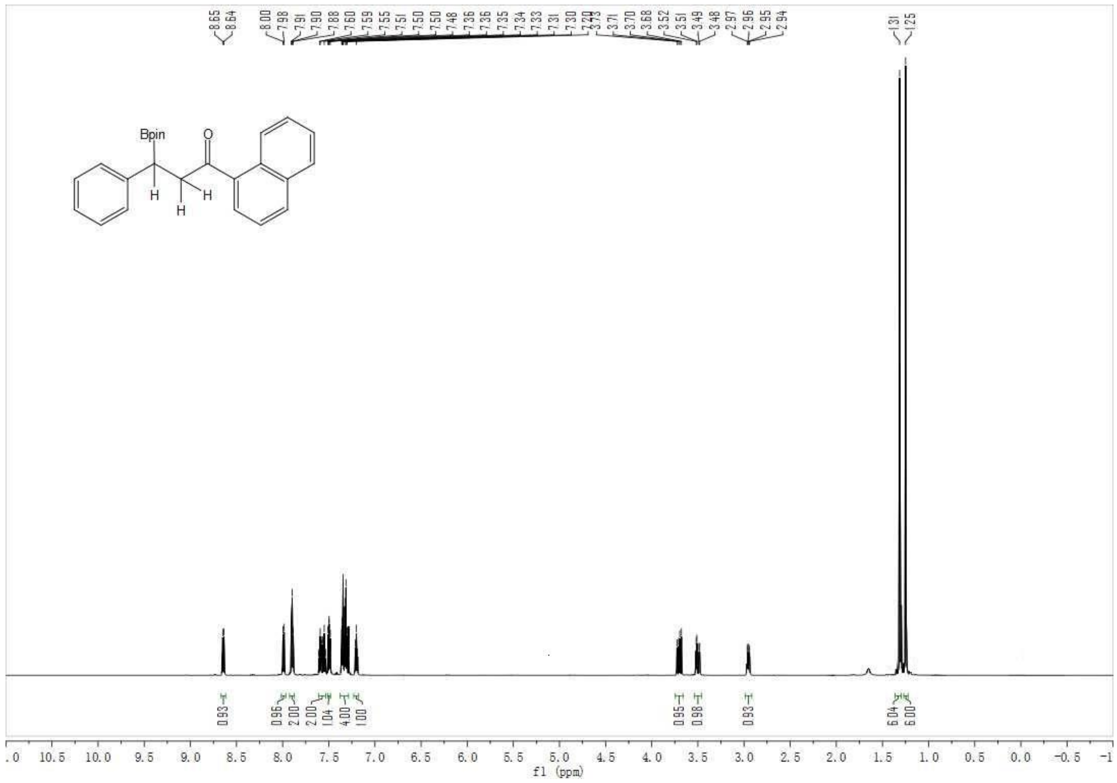

^{13}C{^{1}H} NMR (151 MHz) Spectrum of **3ai** in $CDCl_3$

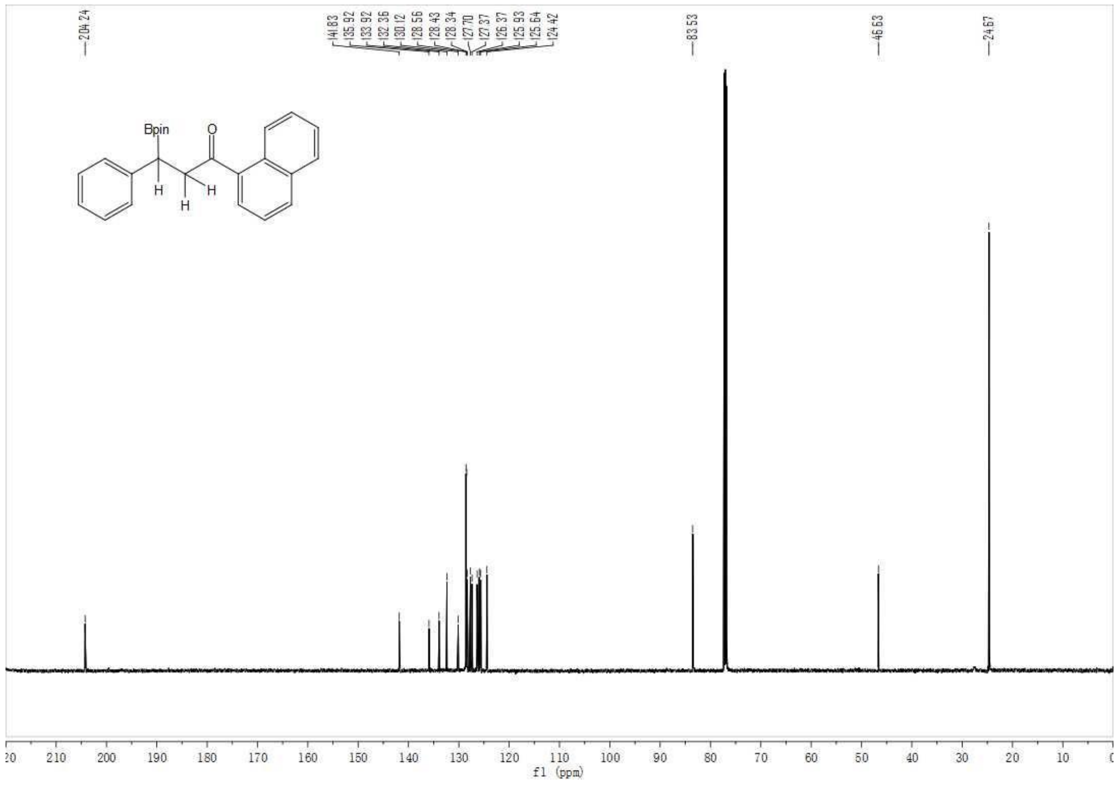

^{11}B NMR (160 MHz) Spectrum of **3ai** in $CDCl_3$

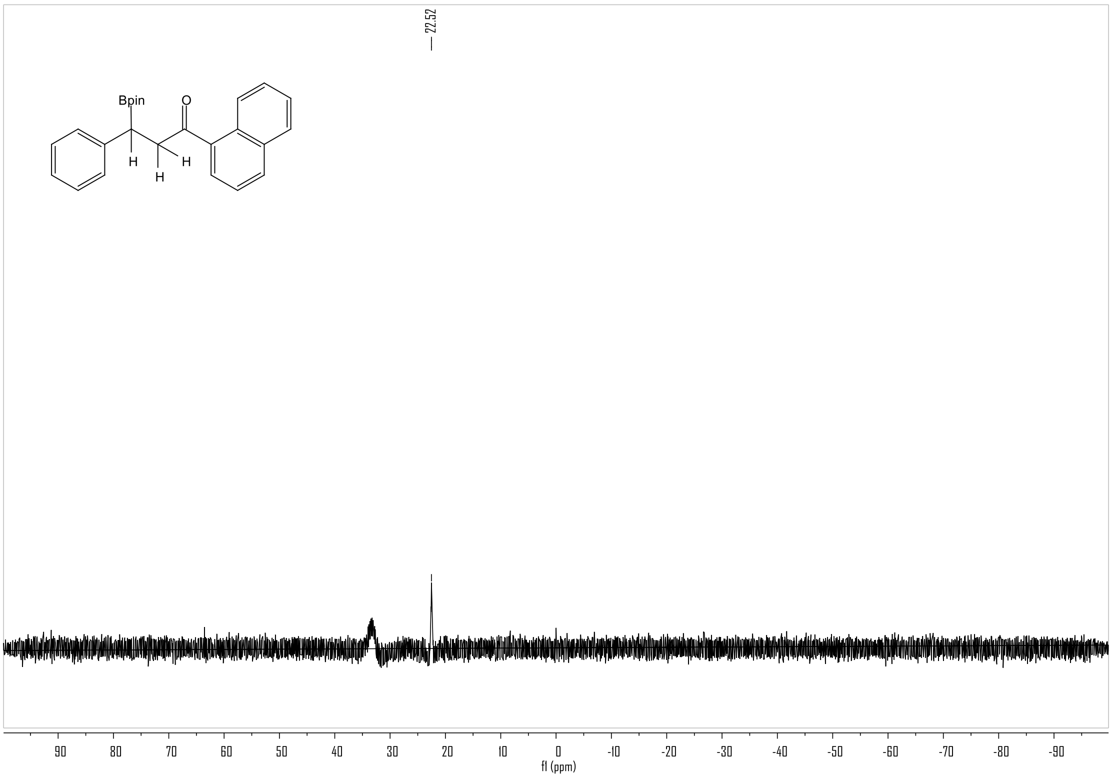

^{1}H NMR (600 MHz) Spectrum of **3aj** in $CDCl_3$

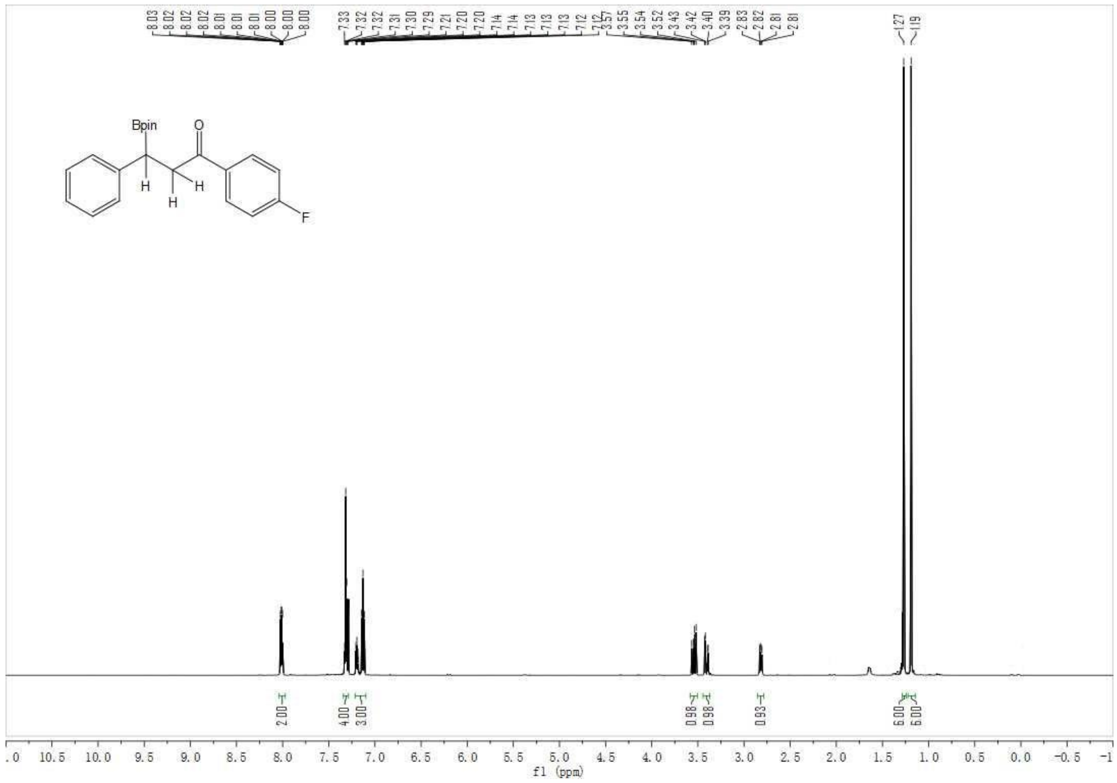

^{13}C{^{1}H} NMR (151 MHz) Spectrum of **3aj** in $CDCl_3$

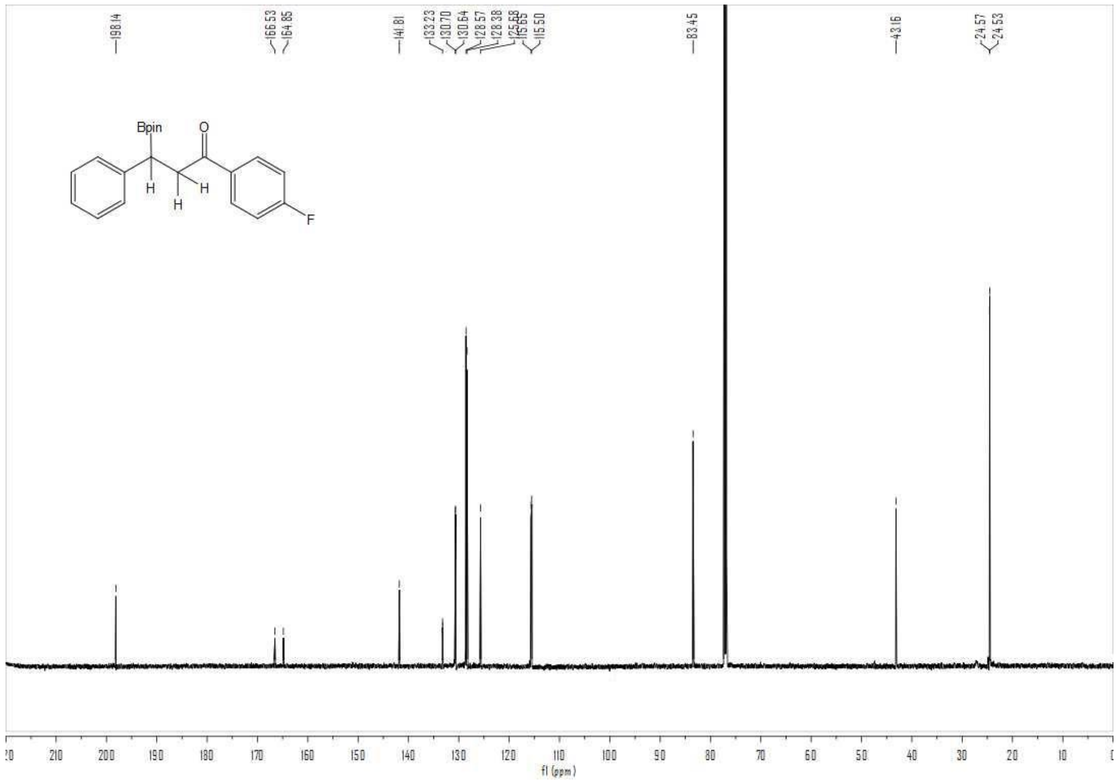

^{19}F NMR (471 MHz) Spectrum of **3aj** in $CDCl_3$

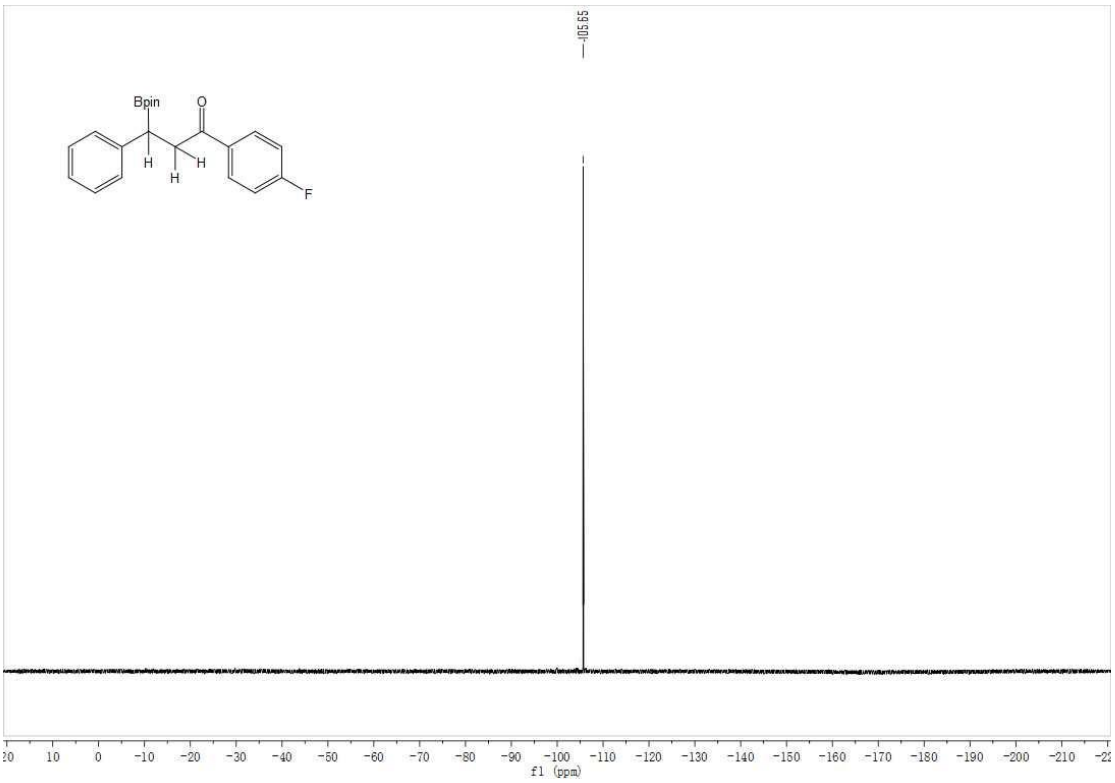

^{11}B NMR (160 MHz) Spectrum of **3aj** in $CDCl_3$

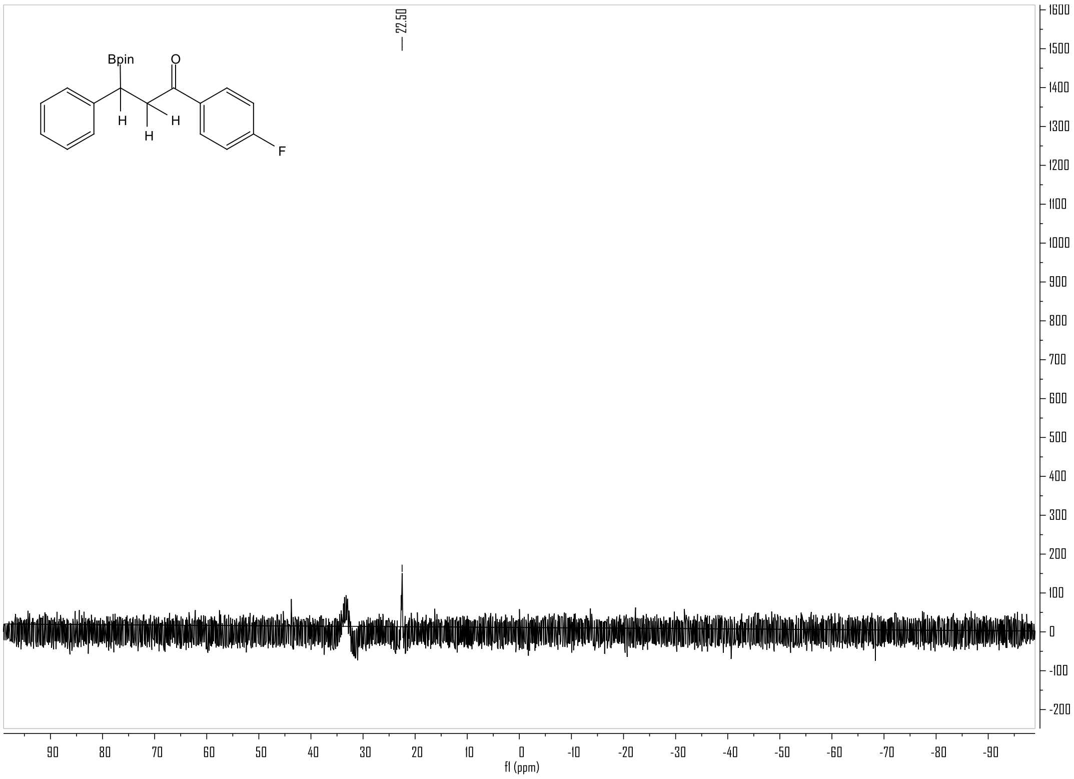

^{1}H NMR (600 MHz) Spectrum of **3ak** in $CDCl_3$

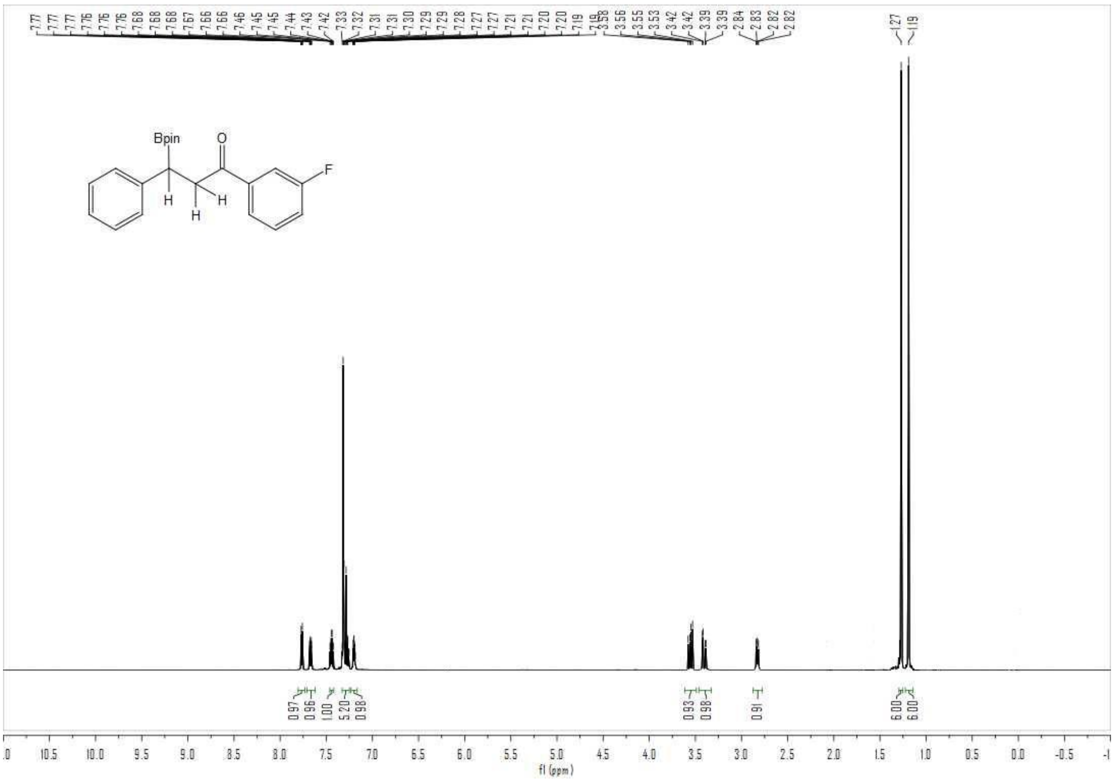

$^{13}C\{^{1}H\}$ NMR (151 MHz) Spectrum of **3ak** in $CDCl_3$

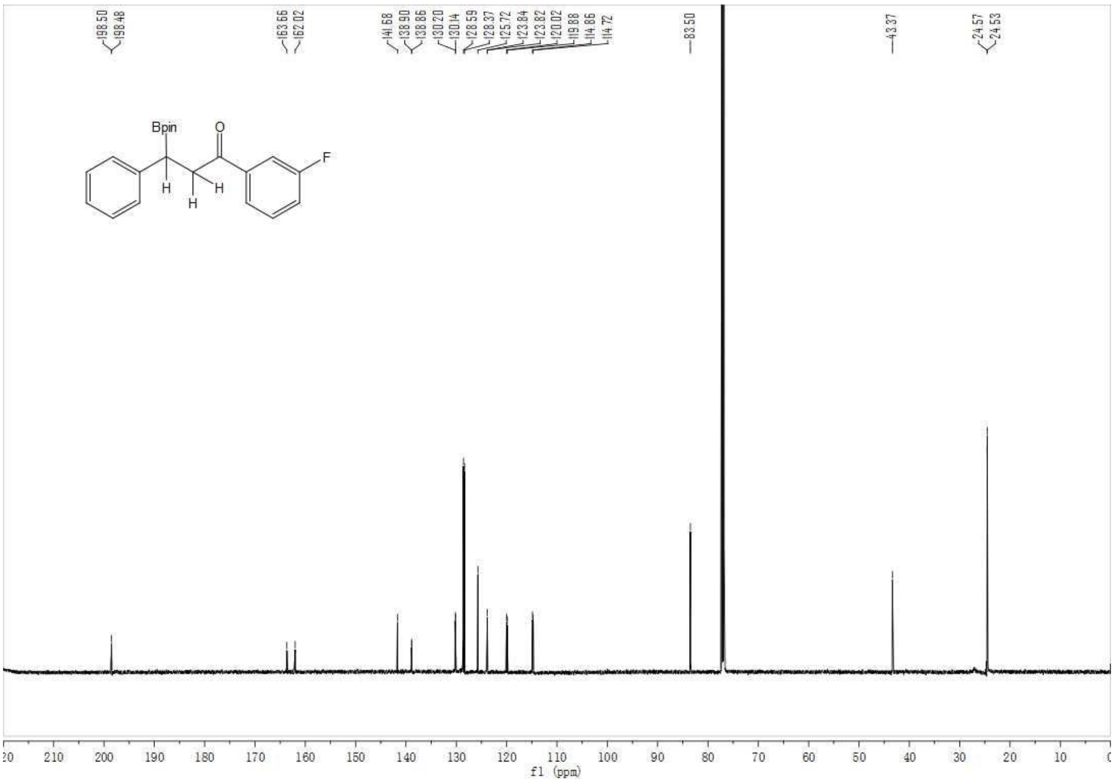

^{19}F NMR (471 MHz) Spectrum of **3ak** in $CDCl_3$

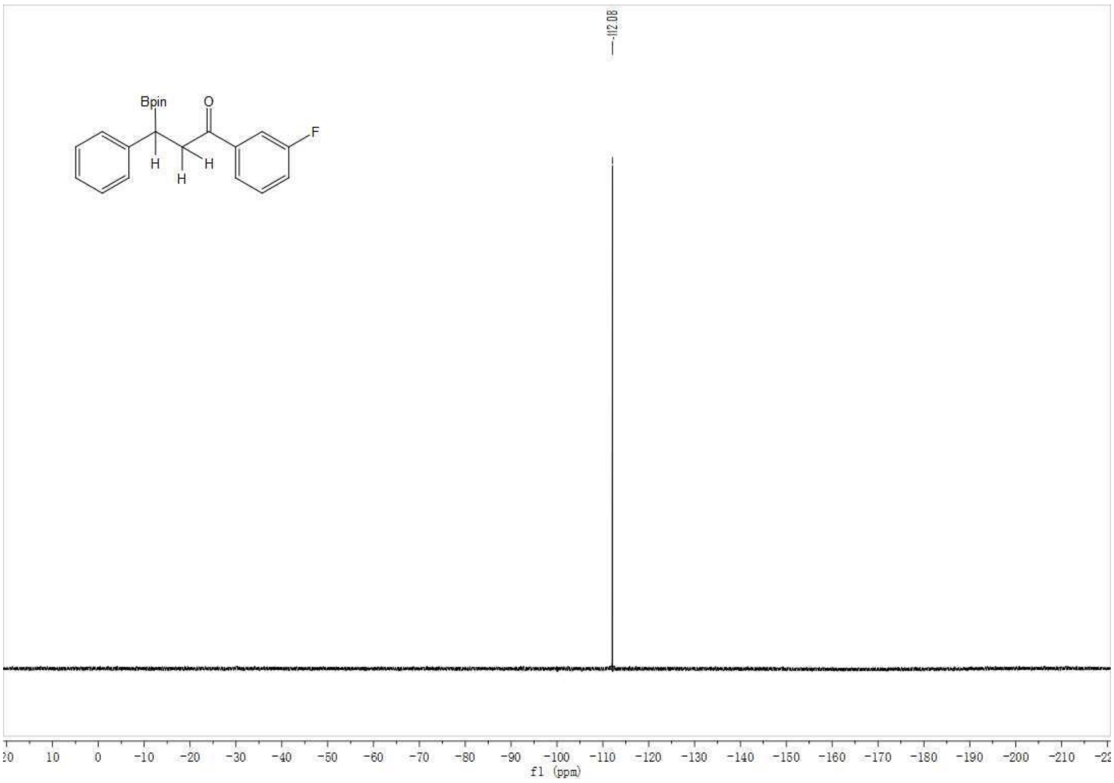

^{11}B NMR (160 MHz) Spectrum of **3ak** in $CDCl_3$

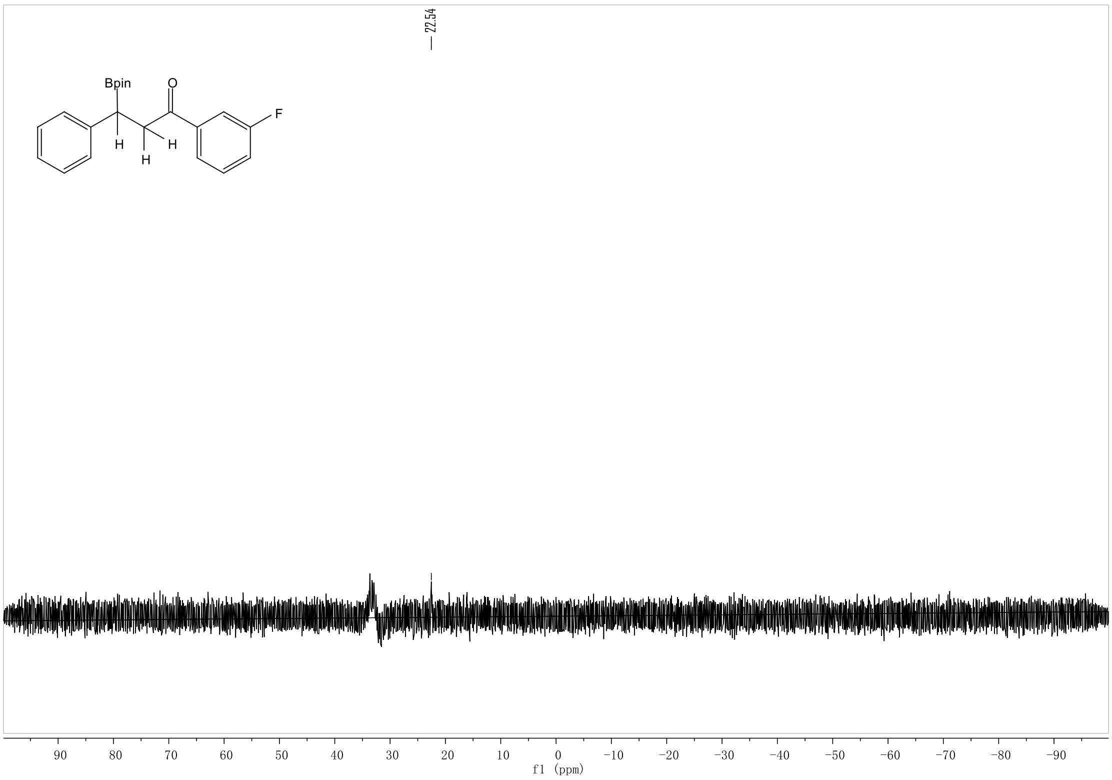

^{1}H NMR (600 MHz) Spectrum of **3al** in $CDCl_3$

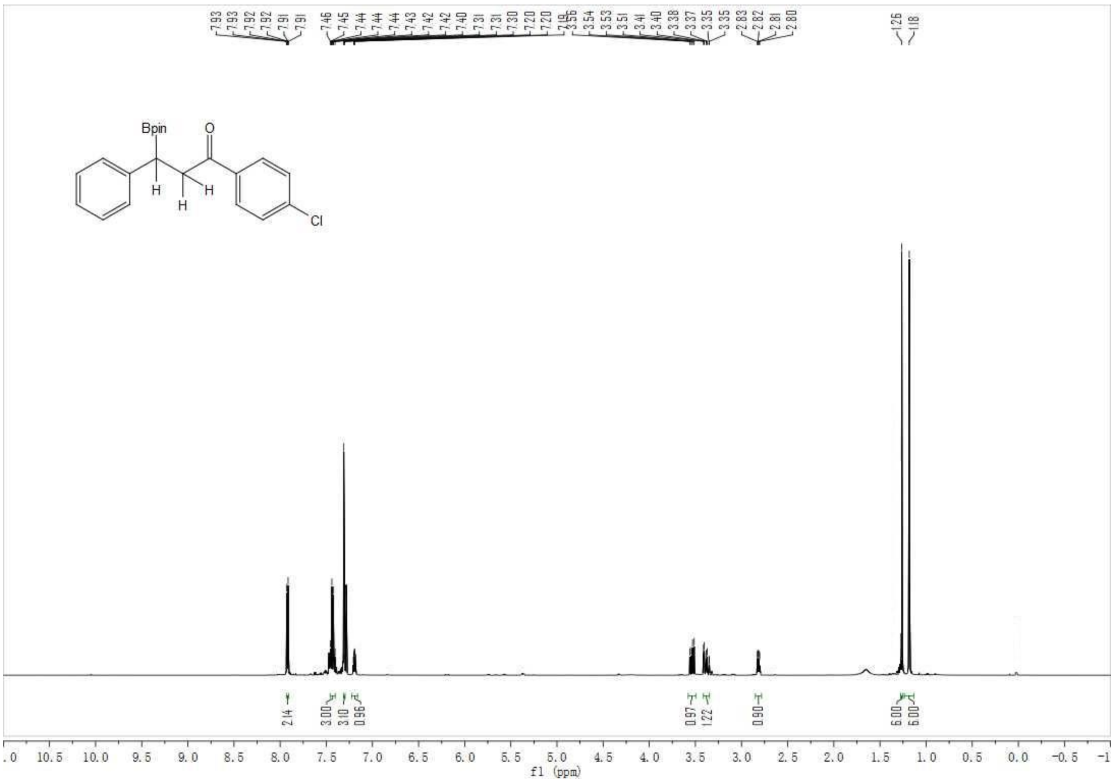

$^{13}C\{^{1}H\}$ NMR (151 MHz) Spectrum of **3al** in $CDCl_3$

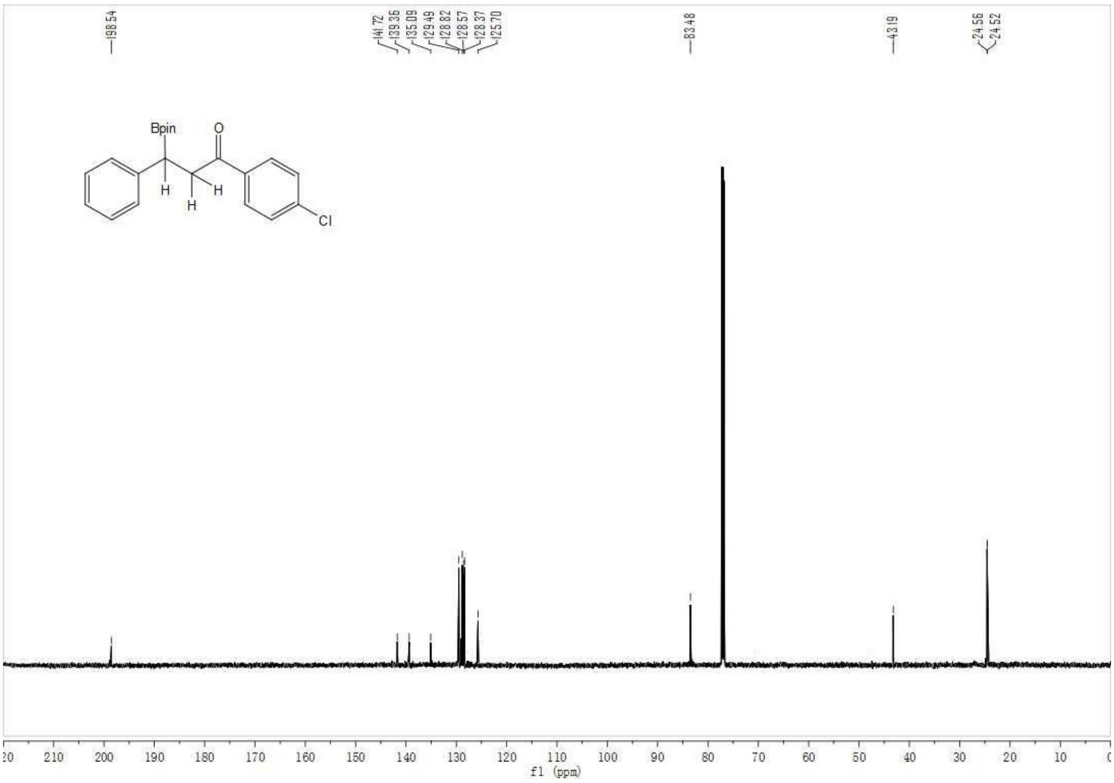

^{11}B NMR (160 MHz) Spectrum of **3al** in $CDCl_3$

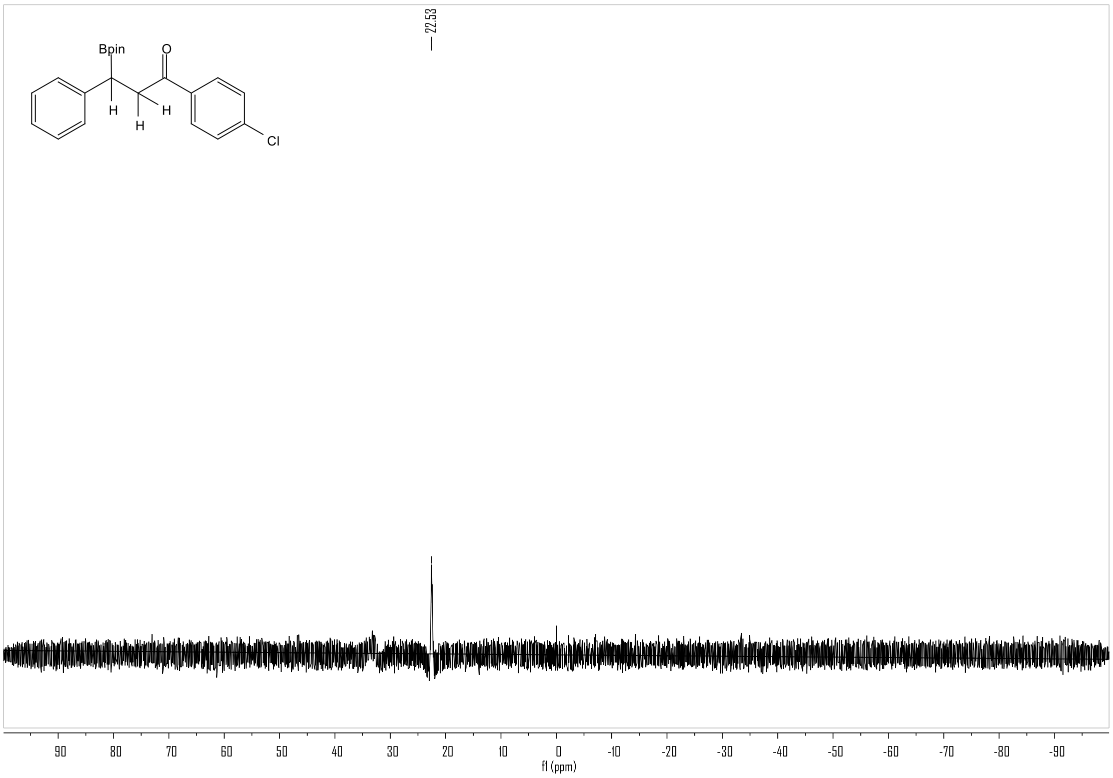

^{1}H NMR (600 MHz) Spectrum of **3am** in $CDCl_3$

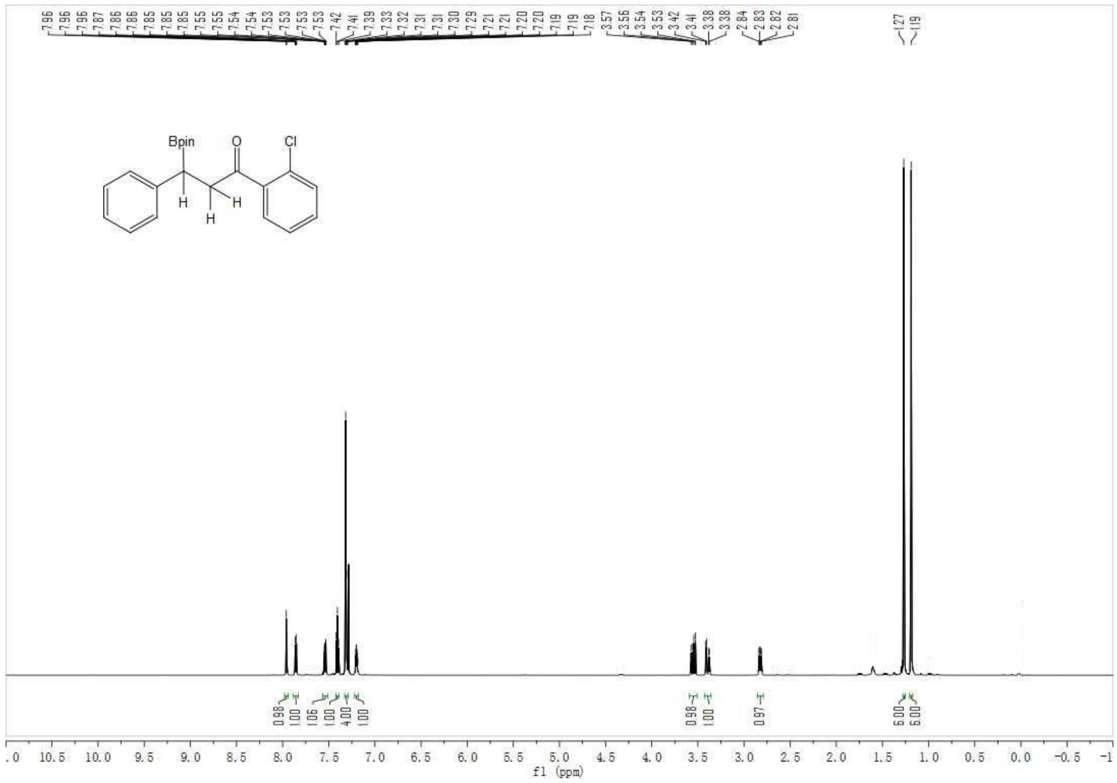

$^{13}C\{^{1}H\}$ NMR (151 MHz) Spectrum of **3am** in $CDCl_3$

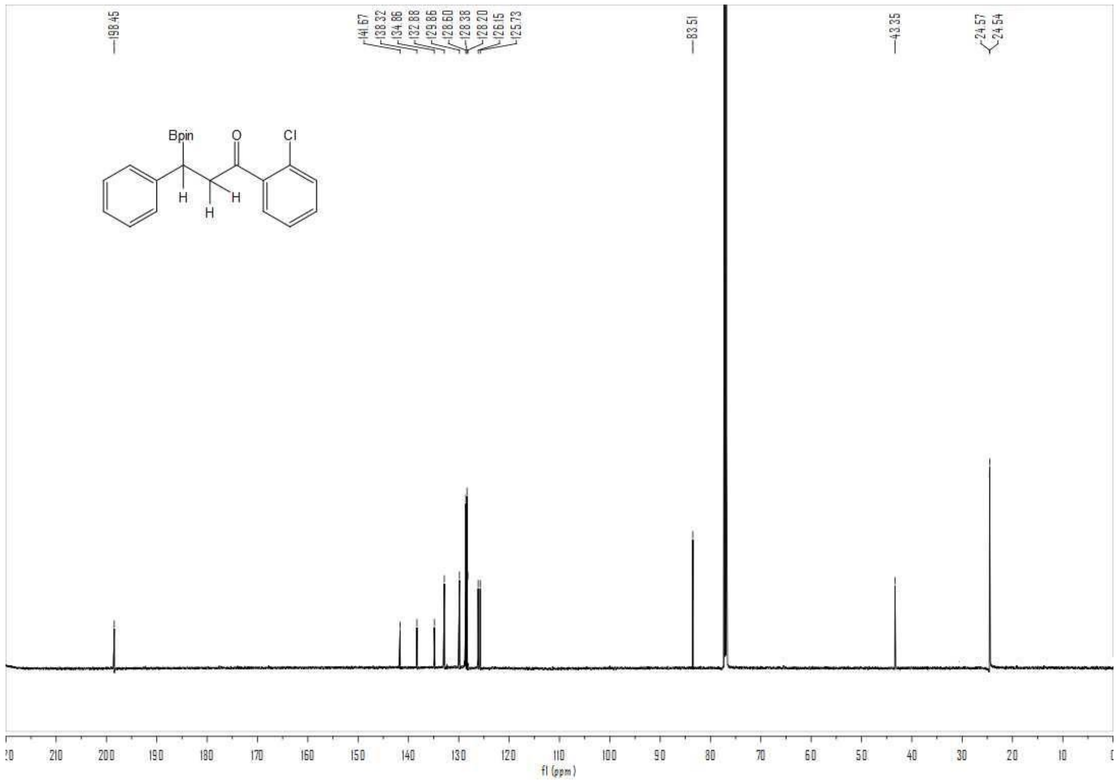

^{11}B NMR (160 MHz) Spectrum of **3am** in $CDCl_3$

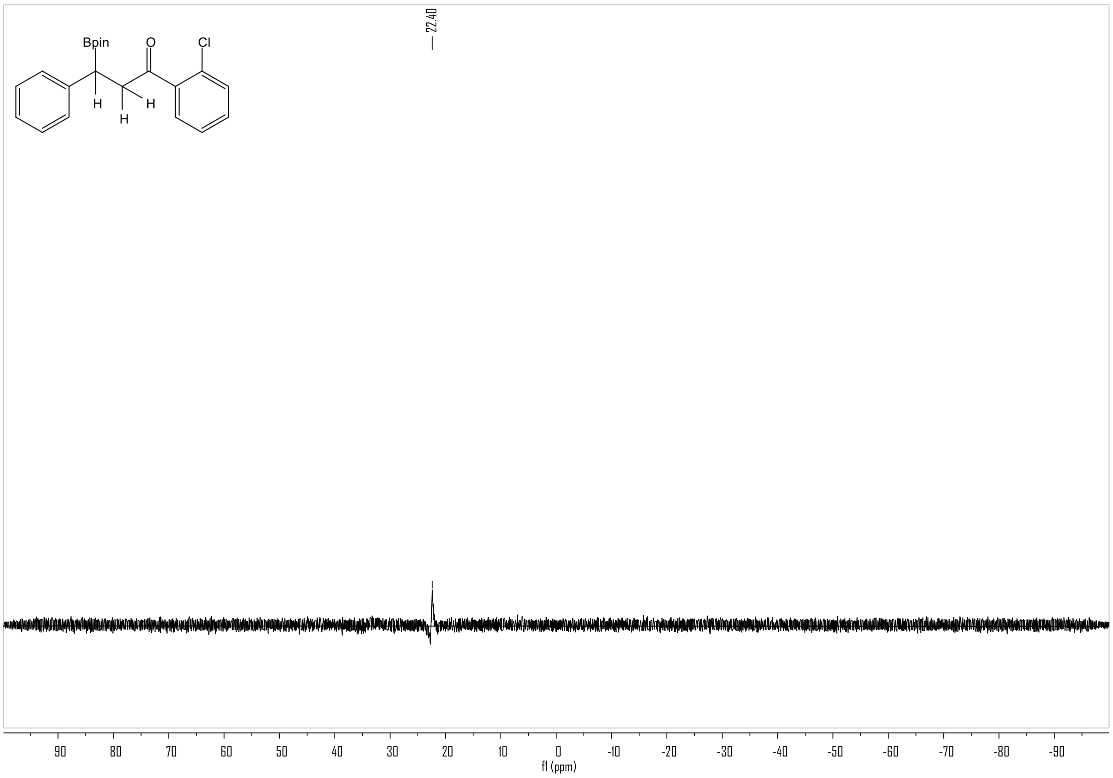

^{1}H NMR (600 MHz) Spectrum of **3an** in $CDCl_3$

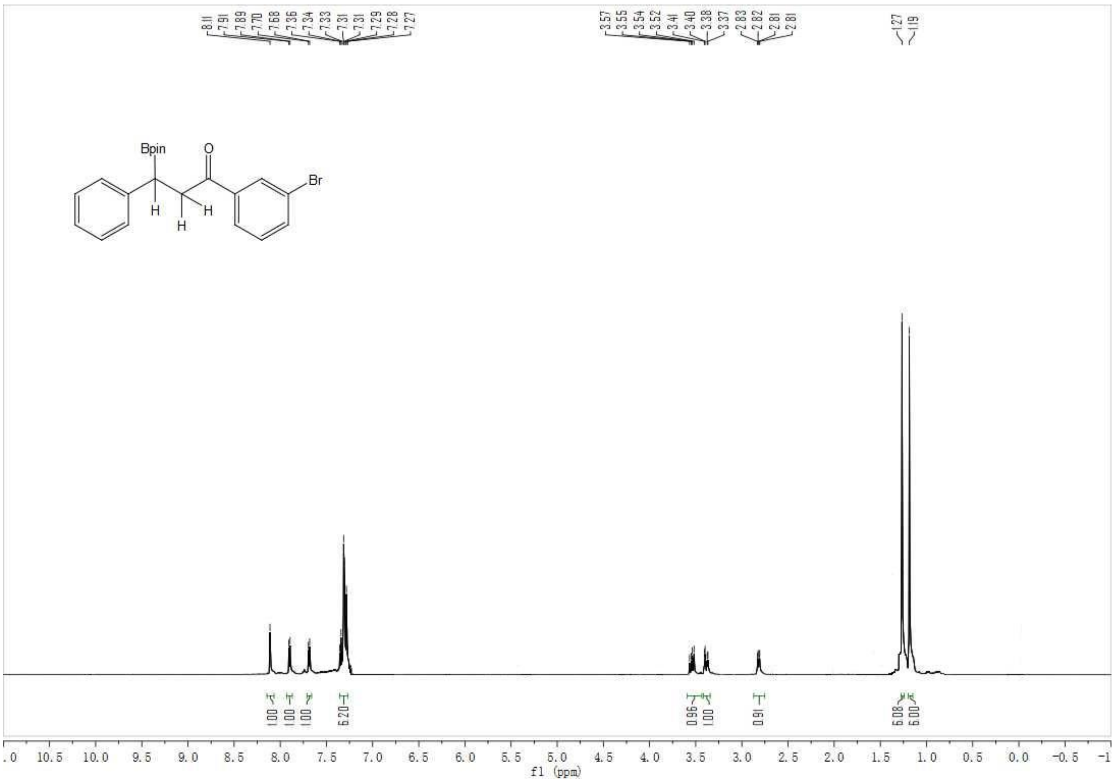

$^{13}C\{^{1}H\}$ NMR (151 MHz) Spectrum of **3an** in $CDCl_3$

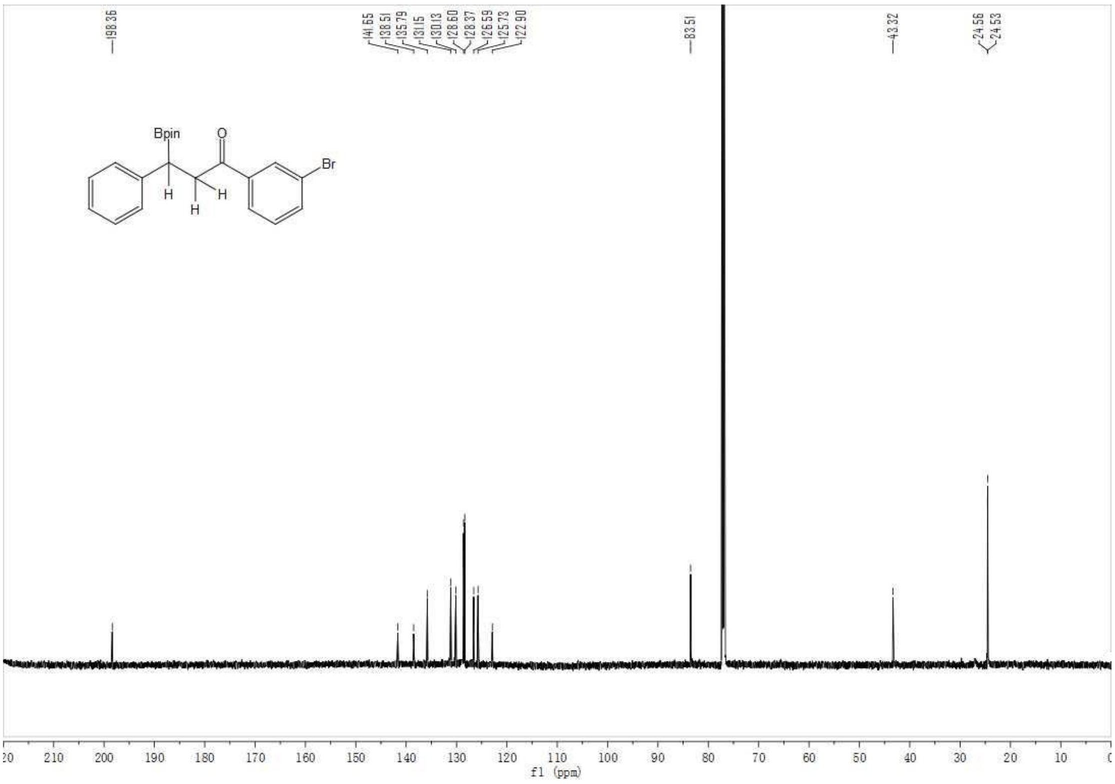

^{11}B NMR (160 MHz) Spectrum of **3an** in $CDCl_3$

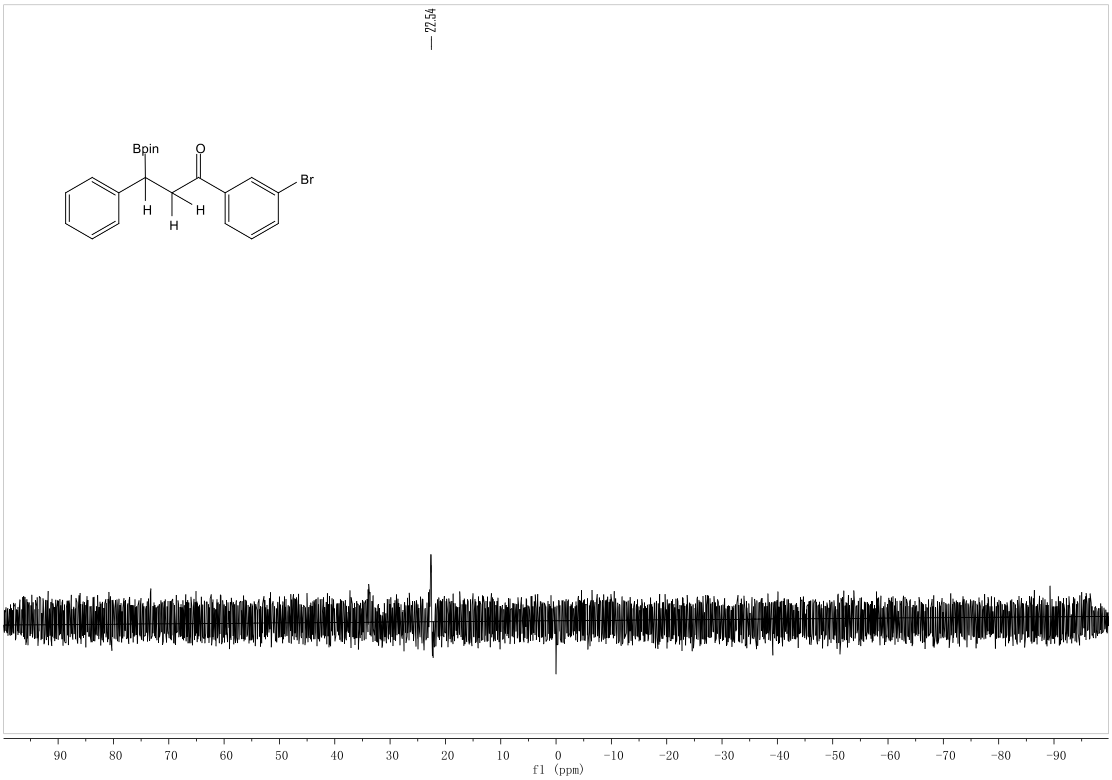

^{1}H NMR (600 MHz) Spectrum of **3aa-D** in $CDCl_3$

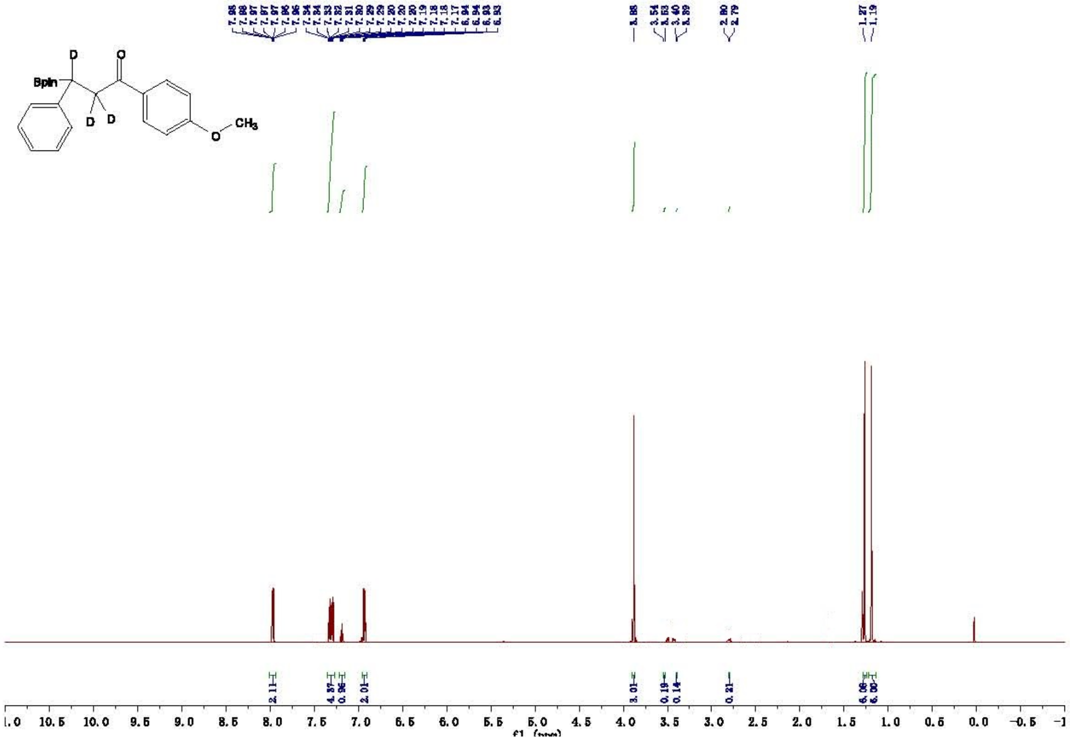

Supplement: SC-013-D2SC04867A-s001 [file SC-013-D2SC04867A-s001.pdf]
